# Effect of the 5:2 Diet on Weight Loss and Cardiovascular Disease Risk Factors in Overweight and/or Obesity: A Systematic Review and Meta-Analysis

**DOI:** 10.1155/ije/6658512

**Published:** 2025-02-24

**Authors:** Cui Wu, Binqin Chen, Jing Yu, Qi Zhang, Chunli Piao

**Affiliations:** ^1^College of Traditional Chinese Medicine, Changchun University of Chinese Medicine, Changchun, Jilin, China; ^2^Applicants with Equivalent Academic Qualifications for Master Degree, Guangzhou University of Chinese Medicine, Guangzhou, Guangdong, China; ^3^Department of Endocrinology, Shenzhen Hospital (Fu Tian) of Guangzhou University of Chinese Medicine, Shenzhen, Guangdong, China; ^4^Department of Endocrinology, Affiliated Hospital to Changchun University of Traditional Chinese Medicine, Changchun, Jilin, China

**Keywords:** 5:2 diet, cardiovascular risk factors, obesity, overweight, weight loss

## Abstract

**Introduction:** We conducted a systematic review and meta-analysis to evaluate the effects of the 5:2 diet on weight loss and cardiovascular disease (CVD) risk factors in overweight and obese individuals.

**Methods:** Databases (PubMed, The Cochrane Library, Web of Science, Embase) were searched for randomized controlled trials of the intervention effects of the 5:2 diet in overweight and obese individuals. The search period was from database establishment to April 2024. RevMan 5.3 and Stata 14.0 were used for the meta-analysis.

**Results:** A total of 20 articles with 1393 participants were finally included. There were 689 participants in the treatment groups and 704 in the control groups. The meta-analysis showed that the 5:2 diet significantly reduces body weight, body mass index, waist circumference, body fat percentage, hip circumference, fat mass, fat-free mass, low-density lipoprotein, systolic blood pressure, and homeostasis model assessment-insulin resistance levels relative to the control group (*p* < 0.05). However, there was no significant difference in levels of visceral fat, total cholesterol, triglycerides, high-density lipoprotein, diastolic blood pressure, insulin, fasting blood glucose, glycated hemoglobin A1c, and heart rate. Although there were no serious adverse events in the 5:2 diet group, participants in this group experienced mild physical and psychological side effects during the fasting period, which resolved spontaneously after fasting.

**Conclusion:** The 5:2 diet is effective for weight reduction and amelioration of CVD risk factors in overweight/obesity and is safe and feasible. However, the patient's physical condition during the fasting period should be monitored and timely adjustments should be made accordingly.

## 1. Introduction

In recent years, the incidence rate of overweight, obesity, and severe obesity has risen sharply with the spread of unhealthy diets and lifestyles and emerged as a global public health problem [1]. Since the end of the last century, the prevalence of obesity, often defined by body mass index (BMI), has doubled in more than 70 countries and continues to rise [2]. For example, the prevalence of adult obesity in the United States increased from 35% in 2010 to 42.4% in 2018 [3]. Obesity can lead to pathological changes in multiple organ systems and cause a variety of systemic metabolic disorder, such as hypertension, hyperlipidemia, and type 2 diabetes (T2DM), which are also risk factors for cardiovascular disease (CVD) [4, 5]. According to reports from 2017, obesity causes approximately 4 million deaths worldwide, with more than two-thirds of these deaths attributed to obesity-related CVD [6]. Therefore, it is critical to develop a global health strategy to control obesity in order to prevent and treat overweight and obesity as well as related CVD.

Dietary intervention therapy is essential for the maintenance of weight loss and sustained glycemic control in overweight or obese individuals [7]. Intermittent fasting—a dietary intervention that refers to no or very low calorie intake for a period of time, without restrictions on eating the rest of the time—has become the focus of research [8, 9]. However, interventions involving intermittent fasting are not standardized. The three most commonly used ones are the 5:2 diet, alternate-day fasting, and time-restricted eating [10]. The 5:2 diet involves fasting for 2 days each week, which may be consecutive or non-consecutive, while allowing unrestricted food consumption on the remaining 5 days. In contrast, alternate-day fasting involves alternating between fasting days and eating days. Time-restricted eating typically requires individuals to fast for 14–16 h daily, with food consumption permitted during a 4- to 12-h window [11]. Compared with these latter two fasting protocols, the 5:2 diet offers greater flexibility regarding fasting duration, enabling individuals to consume food without restriction for 5 days each week, thereby enhancing adherence. Consequently, the 5:2 diet is characterized by enhanced acceptability and improved adherence among individuals practicing fasting. Furthermore, its simplicity and lower demands make it particularly beneficial for vulnerable populations facing high levels of stress, frequent unpredictable events, and limited resources [12]. Due to its straightforwardness and practicality, the 5:2 diet remains a subject of considerable interest and is widely adopted by individuals globally. A randomized, open-label, active parallel-controlled clinical trial involving 405 participants demonstrated that the 5:2 diet resulted in significant improvements in glycemic control and weight reduction among overweight or obese adults with T2DM in the short term, in comparison to treatments with metformin or empagliflozin [13]. Moreover, empirical evidence indicates that the 5:2 diet effectively prevents the onset of nonalcoholic steatohepatitis and ameliorates existing cases of non-alcoholic steatohepatitis and liver fibrosis [14]. In addition, research has demonstrated that the 5:2 diet exerts positive effects on cardiovascular and cerebrovascular diseases, neurodegenerative disorders, cancer, and various other health conditions [15, 16].

However, despite the increasing emphasis on the 5:2 diet, research on weight loss and CVD risk factors in overweight and obesity is limited. In addition, the 5:2 diet can cause a range of reactions in patients, including dizziness, fatigue, mood swings, and constipation [17]. Therefore, there is a need for systematic assessment of the effects of the 5:2 diet in overweight and obesity. This study aims to evaluate the clinical effects of the 5:2 diet on weight loss and CVD risk factors in overweight and obese patients and to provide an evidence-based basis for the development of dietary intervention programs.

## 2. Materials and Methods

The protocol of this study was registered in PROSPERO (https://www.crd.york.ac.uk/prospero/; CRD42024574658) [18]. The study was performed in accordance with Preferred Reporting Program of systematic review and meta-analysis (PRISMA) guidelines (Supporting Table 1).

### 2.1. Data Sources and Search Strategy

We searched PubMed, Web of Science, Cochrane Library, and Embase databases to identify relevant trials published in any language up to 16 April 2024. A combination of subject terms and free words were used, with the main search terms being overweight, obesity, intermittent fasting, and randomized controlled trial (RCT). The search terms and strategy are presented in the Supporting file (Supporting Table 2).

### 2.2. Inclusion Criteria

For inclusions, trials were required to meet the following inclusion criteria (in PICO format): (1) participants: adult patients whose BMI indicated a diagnosis of overweight and/or obesity, stable weight in the last 3 months at the time of study inclusion, no major medical conditions, regardless of gender, disease duration, nationality, or other factors; (2) intervention: implementation of a 5:2 diet (2 consecutive or nonconsecutive days per week, either restricting daily calorie intake by 75%, or restricting the duration of the diet, or low-calorie meal replacements); (3) control: daily energy-restricted diet or routine diet; (4) outcomes: primary outcomes were body weight, BMI, waist circumference, body fat percentage and secondary outcomes were hip circumference, visceral fat, fat mass, fat-free mass, blood lipids, blood pressure, blood glucose, insulin, homeostasis model assessment-insulin resistance (HOMA-IR), and heart rate; and (5) study type: RCTs. Nonrandomized controlled trials, as well as cohort studies and case-control studies, were excluded.

### 2.3. Data Extraction

Independent reviewers assessed the title and abstract of each study using Endnote management software and then subjected the selected papers to full-text screening [19]. Disputes about the inclusion of a study were resolved by discussion and, if necessary, arbitration by a third reviewer. For each included study, two reviewers separately extracted basic data (authors, year, and country); participant characteristics (sample size, age, and gender); intervention characteristics (therapy duration and method of intervention); and outcomes (such as body weight, BMI, and waist circumference, at the baseline and after the trial).

### 2.4. Risk of Bias Assessment

Using the Cochrane Risk of Bias Assessment Tool, two reviewers independently evaluated the risk of bias in the trials and categorized the following items as “high”, “low”, or “unclear”: Random sequence generation; allocation concealment; blinding of participants and personnel; blinding of outcome assessment; selective reporting; and other sources of bias [20].

### 2.5. Data Analysis and Rating Quality of Evidence

Outcome indicators were expressed as post-intervention mean and standard deviation (SD). When the SD could not be directly extracted, it was estimated according to the Cochrane Handbook [21]. Correlation coefficients were used to calculate postintervention SDs if the study only provided baseline and change SDs. The screening and exclusion process is presented in a flowchart, and the characteristics of the included studies were tabulated using appropriate methods and scales. For continuous variables, the weighted mean difference (WMD), 95% confidence interval (CI), was used when the units of measurement were the same; the standardized mean difference (SMD), 95% CI was used when the units of measurement were different. *I*^2^ was used to assess the heterogeneity between studies. If *p* ≥ 0.1 and *I*^2^ ≤ 50, a fixed-effects model was selected for the meta-analysis; otherwise, a random-effects model was used. For literature with clinical or methodological heterogeneity, sensitivity analyses or qualitative descriptions were provided as appropriate. Meta-analyses were performed using RevMan 5.3 software [22]. Funnel plots and Egger's test were used to jointly evaluate publication bias using Stata 14.0 software [23]. Evidence intensity was categorized as high, moderate, low, or very low using the grading of recommendations assessment, development and evaluation (GRADE) approach to grade the quality of evidence for each outcome.

## 3. Results

### 3.1. Basic Characteristics of Included Literature

**Figure 1:**
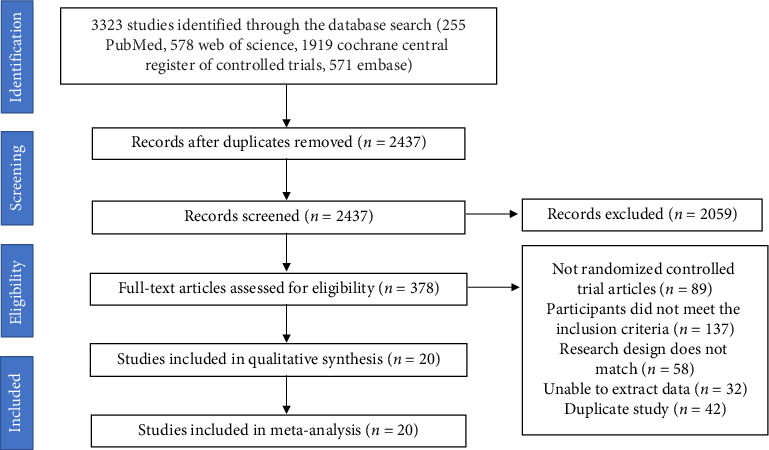
Systematic review flowchart.

An overview of the study selection process is shown in Figure 1. A total of 3323 records were identified using the search strategy. Screening identified 20 RCTs that met the inclusion criteria. The selected studies were published between 2011 and 2022. The treatment duration of the trials ranged from 1 month to 12 months, and the sample size varied from 23 to 173. A total of 1393 patients were included—689 in the treatment group and 704 in the control group—distributed across Asia, North America, Europe, and Oceania. The mean age of the participants ranged from 19 to 68 years, as shown in Table 1. All studies were two-arm trials. The mean baseline of body weight was between 74.1 and 108.6 kg; the mean baseline BMI was between 26.4 and 37.0 kg/m^2^; the mean baseline waist circumference was between 86.3 and 122.5 cm; and the mean baseline body fat percentage was between 29.5% and 44.0%. The baseline data in the literature were consistent.

### 3.2. Risk of Bias Assessment

**Figure 2:**
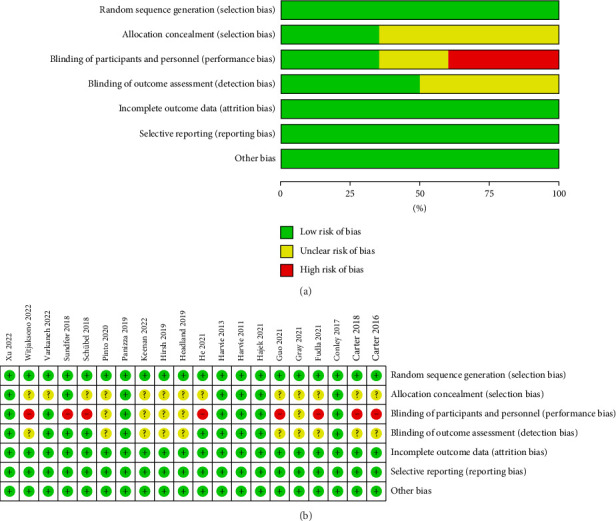
Risk of bias assessment for included studies. (a) Risk of bias graph and (b) risk of bias summary.

Figures 2(a) and 2(b) show the assessment of risk of bias. All included studies used random allocation methods; 7 reported details of allocation concealment methods and the remaining 13 did not report details of allocation concealment and outcome assessment. In this review, 6 studies used a double-blind approach and were assessed as low risk. Because this was a dietary intervention, the practicalities of blinding participants were challenging. Therefore, 8 trials were not blinded to participants and interveners, 5 trials were unclear about whether they were blinded, and 3 trials were single blinded to the staff conducting the outcome assessments to improve the quality of the literature. In addition, all trials described the reasons for those who withdrew from the trial and published complete outcome data. Overall, the literature included in this review was of high quality.

### 3.3. Results of Meta-Analysis of Primary Indicators

#### 3.3.1. Body Weight

**Figure 3:**
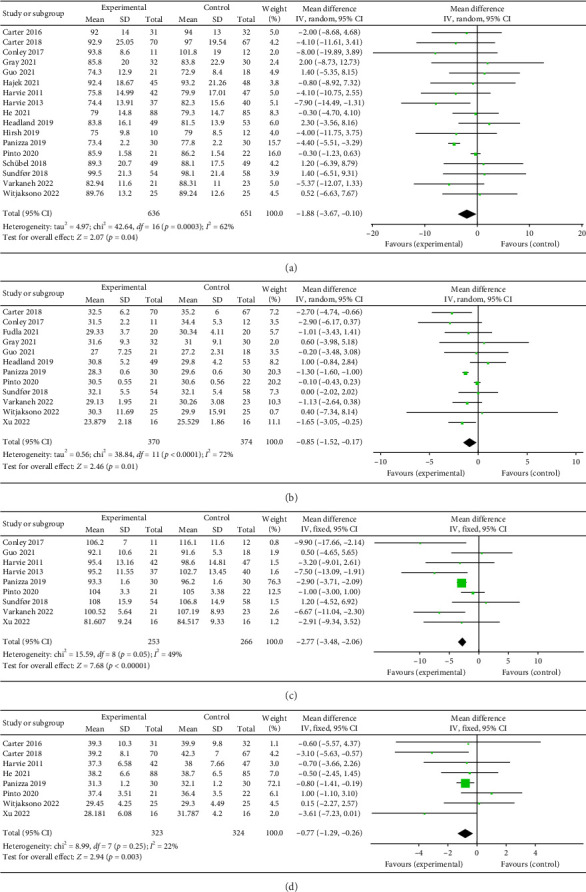
Forest plots of the primary indicators showed that the 5:2 diet could significantly decrease body weight, body mass index (BMI), waist circumference, and body fat percentage in overweight and obese individuals relative to the control group. The green squares denote individual study outcomes, and the black diamonds indicate the aggregated effect sizes. (a) Body weight; (b) BMI; (c) waist circumference; and (d) body fat percentage.

**Figure 4:**
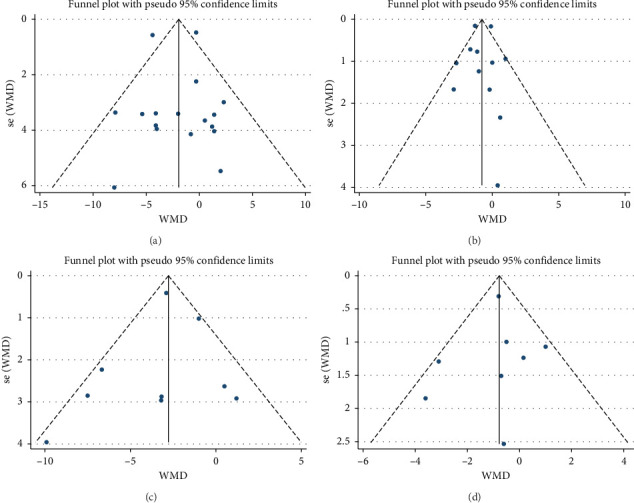
Funnel plots of primary indicators. (a) Body weight; (b) body mass index (BMI); (c) waist circumference; and (d) body fat percentage. The blue dots represent each study included.

**Figure 5:**
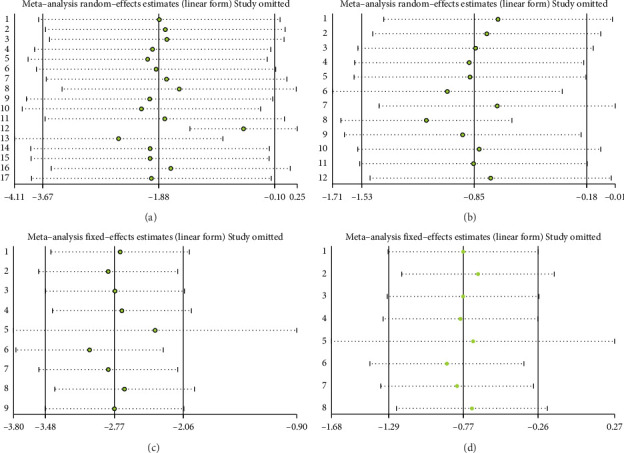
Sensitivity plots of primary indicators. (a) Body weight; (b) body mass index (BMI); (c) waist circumference; and (d) body fat percentage. The green dots represent each study included.

A total of 17 studies [24–40] were included to evaluate the body weight intervention effect of 5:2 diet on overweight and obesity. These studies included 1287 subjects, with 636 in the 5:2 diet group and 651 in the control group. After testing heterogeneity (*I*^2^ = 62%, *p*=0.0003), random effects were selected for meta-analysis. As shown in Figure 3(a), the effect size after meta merge was −1.88 (−3.67, −0.10), and the effect size was significant (*Z* = 2.07 and *p*=0.04). This indicates that the body weights of participants in the 5:2 diet group after treatment were significantly lower than those of the control group by 1.88, and the intervention effect was significant. To examine the publication bias of the current 17 papers, a funnel plot was drawn. As can be clearly seen in Figure 4(a), the funnel plot of this study is essentially symmetrical. Furthermore, Egger's bias test was performed, which yielded *p*=0.999, indicating that there is no publication bias in the literature included in the current study. Sensitivity analysis was performed on the 17 included studies, and the results, as shown in Figure 5(a), indicated that the sensitivity analysis was relatively stable, confirming the validity of the method.

#### 3.3.2. BMI

Twelve studies [24, 25, 27, 28, 33, 35–37, 39–42] were included to evaluate the intervention effect of the 5:2 diet on BMI in overweight and obesity. These studies included 744 subjects, with 374 in the 5:2 diet group and 370 in the control group. There were statistically significant differences in heterogeneity between the studies (*I*^2^ = 72%, *p* < 0.0001); therefore, random effects were selected for meta-analysis. As shown in Figure 3(b), the effect size after meta merging was −0.85 (−1.52, −0.17), and the effect size was significant (*Z* = 2.46, *p*=0.01). This indicates that the BMI of the 5:2 diet group after treatment was significantly lower than that of the control group by 0.85, and the intervention effect was significant. To examine the publication bias of the 12 articles in this study, a funnel plot was constructed. As shown in Figure 4(b), the funnel plot was essentially symmetrical. Furthermore, Egger's bias test was conducted, yielding *p*=0.923. Therefore, it can be concluded that there was no publication bias in the literature included in this study. Sensitivity analysis was conducted, and the results are shown in Figure 5(b). No single study was found to have a strong impact on the research results.

#### 3.3.3. Waist Circumference

A total of 9 studies [26, 28, 30, 31, 35–37, 39, 42] were included to evaluate the intervention effect of the 5:2 diet on waist circumference in overweight and obese individuals, including 519 subjects, with 253 in the 5:2 diet group and 266 in the control group. After heterogeneity testing (*I*^2^ = 49%, *p*=0.05), fixed effects were selected for meta-analysis. As shown in Figure 3(c), the effect size after metamerge was −2.77 (−3.48, −2.06). The effect size was significant (*Z* = 7.68, *p* < 0.00001). This indicates that the waist circumference of the 5:2 diet group after treatment was significantly lower than that of the control group by 2.77, indicating a significant intervention effect. A funnel plot was constructed even though the number of papers was less than 10. As shown in Figure 4(c), the funnel plot of this study was essentially symmetrical. Egger's bias test yielded *p*=0.660, indicating that there is no publication bias in the included studies. Sensitivity analysis was conducted; the results are shown in Figure 5(c). Results of the sensitivity analysis were stable and there was no difference, confirming the effectiveness of this method.

#### 3.3.4. Body Fat Percentage

Eight studies [24, 25, 31, 32, 36, 37, 40, 42] evaluated the effect of the 5:2 diet intervention on body fat percentage in overweight and obese individuals. These studies collectively comprised 647 subjects, including 323 in the 5:2 diet group and 324 in the control group. After heterogeneity testing (*I*^2^ = 22%, *p*=0.25), fixed effects were selected for meta-analysis. The results, as shown in Figure 3(d), showed that the effect size after metamerging was −0.77 (−1.29, −0.26) and the effect size was significant (*Z* = 2.94, *p*=0.003). This indicates a significant reduction in body fat percentage of 0.77 after treatment in the 5:2 diet group relative to the control group, revealing that the effect of the intervention is significant. A funnel plot was constructed even though the number of papers was less than 10. As shown in Figure 4(d), the funnel plot of this study indicates slight deviation, and Egger's bias test yielded *p*=0.785, indicating that there is no publication bias in included studies. Sensitivity analyses were performed on the current 8 papers and the results, as shown in Figure 5(d), showed that the sensitivity analyses were stable and nondifferential, confirming the validity of the methodology.

### 3.4. Results of Meta-Analysis of Blood Lipids

#### 3.4.1. Total Cholesterol (TC)

**Figure 6:**
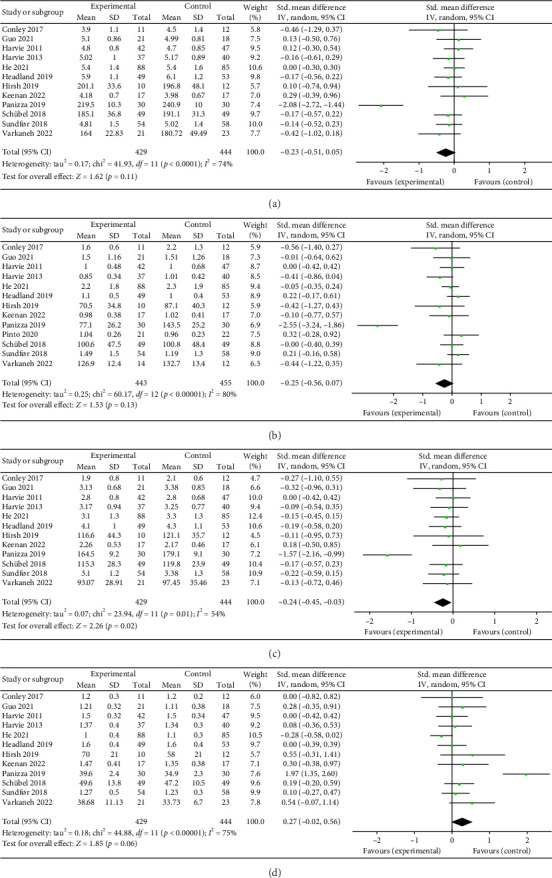
Forest plots of blood lipids revealed that the 5:2 diet resulted in a statistically significant reduction in low-density lipoprotein (LDL) among overweight and obese individuals compared with the control group. However, no significant differences were observed in total cholesterol (TC), triglyceride (TG), or high-density lipoprotein (HDL) levels. The green squares denote individual study outcomes, and the black diamonds indicate the aggregated effect sizes. (a) Total cholesterol (TC); (b) triglyceride (TG); (c) low-density lipoprotein (LDL); and (d) high-density lipoprotein (HDL).

Twelve studies [26, 28, 30–36, 38, 39, 43] were included to evaluate the intervention effect of the 5:2 diet on TC in overweight and obese individuals; these included 873 subjects in total, with 429 in the 5:2 diet group and 444 in the control group. After heterogeneity testing (*I*^2^ = 74%, *p* < 0.0001), random effects were selected for meta-analysis. As shown in Figure 6(a), the effect size after meta merge was −0.23 (−0.51, 0.05); however, the effect size was not significant (*Z* = 1.62, *p*=0.11). This suggests that the 5:2 diet group did not have a significantly lower TC after treatment than the control group.

#### 3.4.2. Triglycerides (TG)

Thirteen studies [26, 28, 30–39, 43] were included to evaluate the intervention effect of the 5:2 diet on TG in overweight and obese individuals. A total of 916 subjects were included, with 443 in the 5:2 diet group and 455 in the control group. After heterogeneity testing (*I*^2^ = 80%, *p* < 0.00001), random effects were selected for meta-analysis. As shown in Figure 6(b), the effect size after meta merging was −0.25 (−0.56, 0.07), but the effect size was not significant (*Z* = 1.53, *p*=0.13). This suggests that the 5:2 diet group did not have a significantly lower TG after treatment than the control group.

#### 3.4.3. Low-Density Lipoprotein (LDL)

Twelve studies [26, 28, 30–36, 38, 39, 43] were included to evaluate the intervention effect of the 5:2 diet on LDL in overweight and obese individuals, including 873 subjects, with 429 in the 5:2 diet group and 444 in the control group. After heterogeneity testing (*I*^2^ = 54%, *p*=0.01), random effects were selected for meta-analysis. As shown in Figure 6(c), the effect size after meta merging was −0.24 (−0.45, −0.03), and the effect size was significant (*Z* = 2.26, *p*=0.02). The results showed that the LDL levels in the 5:2 diet group were significantly lower, by 0.24, than those in the control group after treatment, indicating a significant intervention effect.

#### 3.4.4. High-Density Lipoprotein (HDL)

Twelve studies [26, 28, 30–36, 38, 39, 43] were included to evaluate the intervention effect of 5:2 diet on HDL in overweight and obese individuals. These studies collectively included 873 subjects, with 429 in the 5:2 diet group and 444 in the control group. After heterogeneity testing (*I*^2^ = 75%, *p* < 0.00001), random effects were selected for meta-analysis. As shown in Figure 6(d), the effect size after metamerging was 0.27 (−0.02, 0.56), although this was not significant (*Z* = 1.85, *p*=0.06). This suggests that the 5:2 diet group did not have a significantly higher HDL after treatment than the control group.

To test for publication bias in the literature, funnel plots were constructed. As shown in Supporting Figure 1, the funnel plot of this study appears to indicate slight bias. Furthermore, Egger's bias test indicated that there was no significant publication bias in the included literature. Sensitivity analysis was conducted on the included literature, and the results are shown in Supporting Figure 2. The sensitivity analysis results for this study are relatively stable, indicating that the results are acceptable.

### 3.5. Results of Meta-Analysis of Blood Pressure and Heart Rate

#### 3.5.1. Systolic Blood Pressure (SBP)

**Figure 7:**
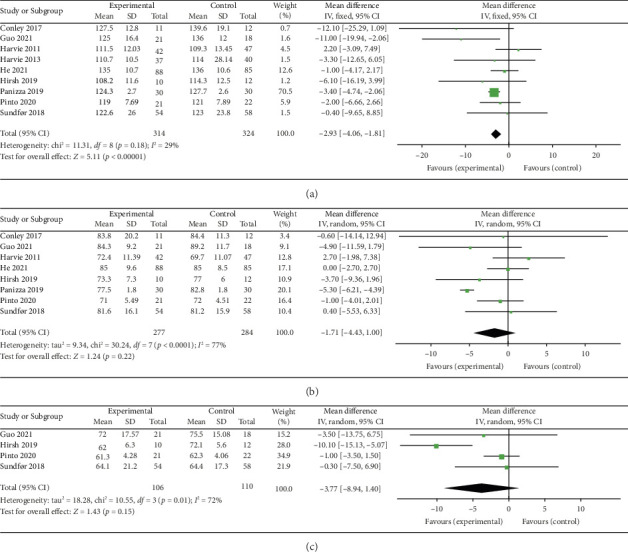
Forest plots of blood pressure and heart rate showed that the 5:2 diet resulted in a statistically significant reduction in systolic blood pressure (SBP) among overweight and obese individuals compared to the control group. However, no significant differences were observed in diastolic blood pressure (DBP) or heart rate. The green squares denote individual study outcomes, and the black diamonds indicate the aggregated effect sizes. (a) Systolic blood pressure (SBP); (b) diastolic blood pressure (DBP); and (c) heart rate.

Nine studies [26, 28, 30–32, 34, 36, 37, 39] were included to evaluate the intervention effect of the 5:2 diet on SBP in overweight and obese individuals. The studies included 638 subjects, with 314 in the 5:2 diet group and 324 in the control group. After heterogeneity testing (*I*^2^ = 29%, *p*=0.18), fixed effects were selected for meta-analysis. As shown in Figure 7(a), the effect size after metamerging was −2.93 (−4.06, −1.81), and the effect size was significant (*Z* = 5.11, *p* < 0.00001). The results showed that the SBP levels in the 5:2 diet group were significantly lower, by 2.93, than those of the control group after treatment, indicating a significant intervention effect. The funnel plot was observed even though the number of papers was less than 10. The funnel plot for this study is essentially symmetrical (Supporting Figure 3A); furthermore, Egger's bias test yielded *p*=0.858, indicating that there is no publication bias in the literature of this study. Sensitivity analysis results were relatively stable (Supporting Figure 4A), indicating that the results are acceptable.

#### 3.5.2. Diastolic Blood Pressure (DBP)

Eight studies [26, 28, 30, 32, 34, 36, 37, 39] were included to evaluate the intervention effect of the 5:2 diet on DBP in overweight and obesity; these included 561 subjects, with 277 in the 5:2 diet group and 284 in the control group. After heterogeneity testing (*I*^2^ = 77%, *p* < 0.0001), random effects were selected for meta-analysis. As shown in Figure 7(b), the effect size after meta merging was −1.71 (−4.43, 1.00), but the effect size was not significant (*Z* = 1.24, *p*=0.22). This suggests that the 5:2 diet group did not have a significantly lower DBP after treatment than the control group. The funnel plot had slight bias (Supporting Figure 3B), and Egger's bias test yielded *p*=0.056, indicating that there was no significant publication bias in the included literature. The sensitivity analysis is relatively stable, indicating that the results are acceptable (Supporting Figure 4B).

#### 3.5.3. Heart Rate

Four studies [28, 34, 37, 39] evaluated the effect of the 5:2 diet intervention on heart rate in overweight and obese individuals. These studies comprised 216 subjects, including 106 in the 5:2 diet group and 110 in the control group. After heterogeneity testing (*I*^2^ = 72%, *p*=0.01), random effects were selected for meta-analysis. The results, as shown in Figure 7(c), showed that the effect size after meta-merging was −3.77 (−8.94, 1.40) and not significant (*Z* = 1.43, *p*=0.15). This shows the insignificant effect of reduction of heart rate after treatment in the 5:2 diet group compared with that in the control group. The funnel plot indicated slight bias (Supporting Figure 3C), and Egger's bias test yielded *p*=0.625, confirming that there was no significant publication bias in the included literature. Sensitivity analyses (Supporting Figure 4C) were stable and nondifferential.

### 3.6. Results of Meta-Analysis of Glucose Metabolism

#### 3.6.1. HOMA-IR

**Figure 8:**
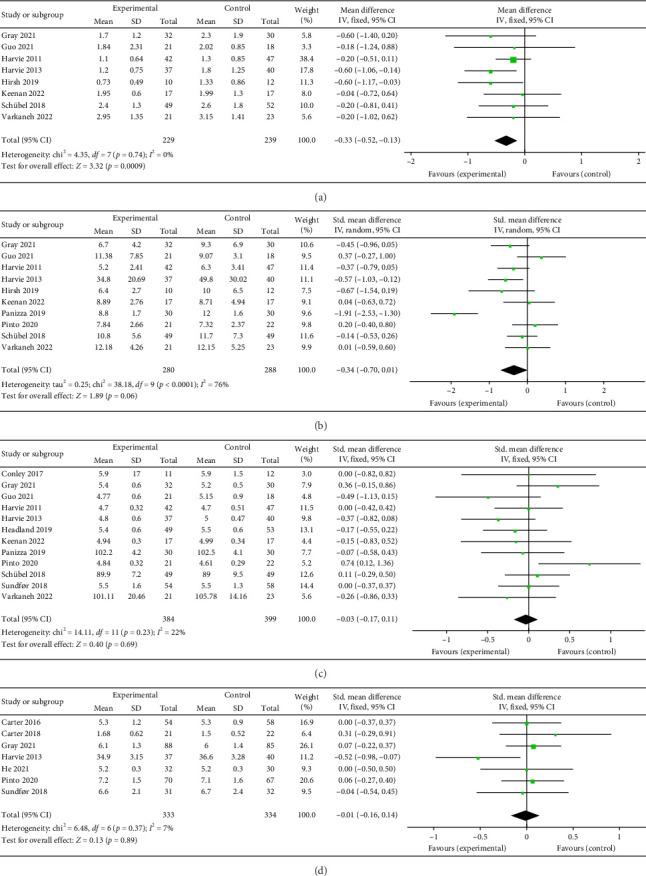
Forest plots of glucose metabolism showed that the 5:2 diet resulted in a statistically significant reduction in HOMA-IR among overweight and obese individuals compared to the control group. However, no significant differences were observed in insulin, fasting blood glucose (FBG), or HbA1c. The green squares denote individual study outcomes, and the black diamonds indicate the aggregated effect sizes. (a) HOMA-IR; (b) insulin; (c) fasting blood glucose (FBG); and (d) HbA1c.

Eight papers [27, 28, 30, 31, 34, 35, 38, 43] evaluating the effect of the 5:2 diet on the HOMA-IR intervention in overweight and obesity contained 468 subjects, with 229 in the 5:2 diet group and 239 in the control group. After heterogeneity testing (*I*^2^ = 0%, *p*=0.74), fixed effects were selected for meta-analysis. The results, as shown in Figure 8(a), indicated that the effect size after meta-merging was −0.33 (−0.52, −0.13) and the effect size was significant (*Z* = 3.32, *p*=0.0009). This illustrates that the intervention effect of the 5:2 diet was significant, with the intervention group having a significantly lower HOMA-IR of 0.33 after treatment compared with the control group. The funnel plot was essentially symmetrical (Supporting Figure 5A), and Egger's bias test yielded *p*=0.838, indicating that there was no significant publication bias in the included literature. Sensitivity analyses (Supporting Figure 6A) results were stable and nondifferentiated, confirming the validity of the methodology.

#### 3.6.2. Insulin

A total of 10 papers [27, 28, 30, 31, 34–38, 43] evaluating the effect of the 5:2 diet on insulin intervention in overweight and obesity contained 568 subjects, with 280 in the 5:2 diet group and 288 in the control group. After heterogeneity testing (*I*^2^ = 76%, *p* < 0.0001), random effects were selected for meta-analysis. The results, as shown in Figure 8(b), indicated that the effect size after metamerging was −0.34 (−0.70, 0.01) and the effect size was not significant (*Z* = 1.89, *p*=0.06). This suggests that the 5:2 diet group did not have significantly lower insulin levels after treatment than the control group. The funnel plot is basically symmetrical (Supporting Figure 5B), and Egger's bias test, *p*=0.892, indicated that there was no significant publication bias in the included literature. Sensitivity analyses showed that the sensitivity analysis results were stable and nondifferentiated (Supporting Figure 6B).

#### 3.6.3. Fasting Blood Glucose (FBG)

A total of 12 papers [26–28, 30, 31, 33, 35–39, 43] evaluating the effect of 5:2 diet on FBG intervention in overweight and obesity contained 783 subjects, with 384 subjects in the 5:2 diet group and 399 individuals in the control group. After heterogeneity testing (*I*^2^ = 22%, *p*=0.23), fixed effects were selected for meta-analysis. The results, as shown in Figure 8(c), indicated that the effect size after meta-merging was −0.03 (−0.17, 0.11) and significant (*Z* = 0.40, *p*=0.69). This suggests that the 5:2 diet group did not have a significantly lower FBG after treatment than the control group. The funnel plot was essentially symmetrical (Supporting Figure 5C), and Egger's bias test yielded *p*=0.939, indicating that there was no significant publication bias in the included literature. Results of sensitivity analyses were stable and nondifferentiated (Supporting Figure 6C).

#### 3.6.4. Glycated Hemoglobin A1c (HbA1c)

Seven papers [24, 25, 27, 29, 32, 37, 39] evaluating the effect of the 5:2 diet on HbA1c levels in overweight and obese individuals contained 667 subjects, including 333 in the 5:2 diet group and 334 in the control group. After heterogeneity testing (*I*^2^ = 7%, *p*=0.37), fixed effects were selected for meta-analysis. The results, as shown in Figure 8(d), indicated that the effect size after metamerging was −0.01 (−0.16, 0.14) and the effect size was not significant (*Z* = 0.13, *p*=0.89). This suggests that the 5:2 diet group did not have a significantly lower HbA1c after treatment than the control group. The funnel plot indicated slight bias (Supporting Figure 5D) and Egger's bias test yielded *p*=0.771, indicating that there was no significant publication bias in the included literature. Results of sensitivity analyses were stable and nondifferentiated (Supporting Figure 6D).

### 3.7. Results of Meta-Analysis of Other Secondary Indicators

#### 3.7.1. Hip Circumference

**Figure 9:**
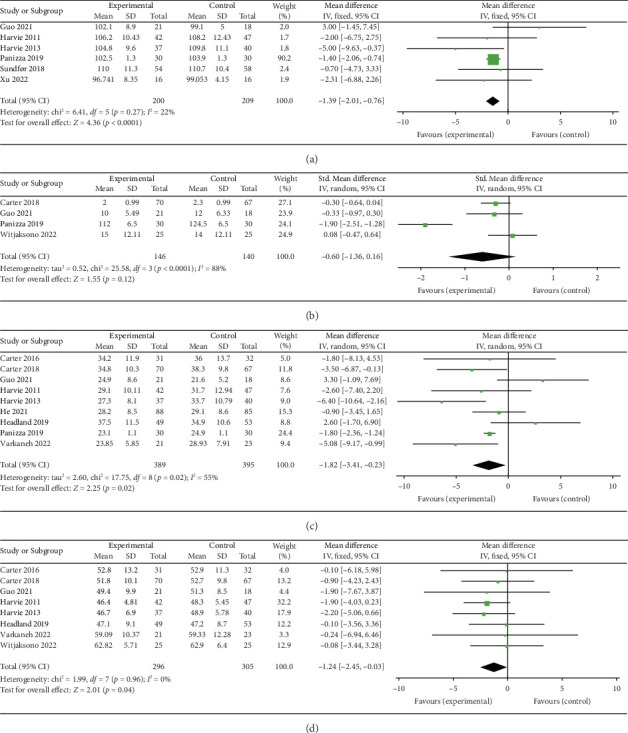
Forest plots of other secondary indicators showed that the 5:2 diet could significantly decrease hip circumference, fat mass, and fat-free mass in overweight and obese individuals relative to the control group. However, no significant differences were observed in visceral fat. The green squares denote individual study outcomes, and the black diamonds indicate the aggregated effect sizes. (a) Hip circumference; (b) visceral fat; (c) fat mass; (d) fat-free mass.

Six studies [28, 30, 31, 36, 39, 42] evaluated the effect of 5:2 diet on hip circumference intervention in overweight and obese individuals, comprising 409 subjects, with 200 in the 5:2 diet group and 209 in the control group. After heterogeneity testing (*I*^2^ = 22%, *p*=0.27), fixed effects were selected for meta-analysis. The results showed (Figure 9(a)) that the effect size after metamerging was −1.39 (−2.01, −0.76) and the effect size was significant (*Z* = 4.36, *p* < 0.0001). This illustrates the significant intervention effect of a 1.39 reduction in hip circumference after treatment in the 5:2 diet group relative to the control group. The funnel plot was symmetrical (Supporting Figure 7A), and Egger's bias test yielded *p*=0.980, indicating that there was no publication bias in the included literature. Sensitivity analysis was performed on the present six papers and the results, as shown in Supporting Figure 8A, were stable and nondifferential, confirming the validity of the methodology.

#### 3.7.2. Visceral Fat

Four studies [25, 28, 36, 40] evaluated the effect of the 5:2 diet intervention on visceral fat in overweight and obese individuals. The studies comprised 286 subjects, with 146 in the 5:2 diet group and 140 in the control group. After heterogeneity testing (*I*^2^ = 88%, *p* < 0.0001), random effects were selected for meta-analysis. The results showed (Figure 9(b)) that the effect size after metamerging was −0.60 (−1.36, 0.16) but the effect size was not significant (*Z* = 1.55, *p*=0.12). This indicates that the reduction of visceral fat after treatment in the 5:2 diet group was not significant relative to the control group. The funnel plot revealed slight bias (Supporting Figure 7B) and Egger's bias test yielded *p*=0.618, indicating that there was no significant publication bias in the included literature. Sensitivity analyses performed on the current 4 papers (Supporting Figure 8B) were stable and nondifferential.

#### 3.7.3. Fat Mass

Nine studies [24, 25, 28, 30–33, 35, 36] evaluating the effect of 5:2 diet on fat mass intervention in overweight and obese individuals comprised 784 subjects, including 389 in the 5:2 diet group and 395 in the control group. After heterogeneity testing (*I*^2^ = 55%, *p*=0.02), random effects were selected for meta-analysis. The results, as shown in Figure 9(c), indicated that the effect size after metamerging was −1.82 (−3.41, −0.23) and significant (*Z* = 2.25, *p*=0.02). This illustrates the significant effect of the intervention in terms of reducing the fat mass significantly by 1.82 after treatment in the 5:2 diet group relative to the control group. The funnel plot was essentially symmetrical (Supporting Figure 7C), and Egger's bias test yielded *p*=0.989, indicating that there was no publication bias in the included literature. Sensitivity analysis was performed and the results, as shown in Supporting Figure 8C, were stable and nondifferential, confirming the validity of the methodology.

#### 3.7.4. Fat-Free Mass

Nine studies [24, 25, 28, 30, 31, 33, 35, 36, 40] evaluated the effect of 5:2 diet intervention on fat-free mass in overweight and obesity. These studies comprised 661 subjects, including 326 in the 5:2 diet group and 335 in the control group. After heterogeneity testing (*I*^2^ = 0%, *p*=0.70), fixed effects were selected for meta-analysis. The result of the meta-analysis was −2.17 (−2.90, −1.44) and the effect size was significant (*Z* = 5.85, *p* < 0.00001). However, the results of the sensitivity analyses showed that one trial (Panizza et al.) [36] stood out, and the Egger's bias test yielded *p*=0.005. As a result, this trial was excluded. The results showed that the effect size after metamerging was −1.24 (−2.45, −0.03) and significant (*Z* = 2.01, *p*=0.04) (Figure 9(d)). This illustrates the significant effect of the intervention, given the fat-free mass after treatment in the 5:2 diet group was significantly lower than that of the control group, by 1.24. The funnel plot was essentially symmetrical (Supporting Figure 7D), and Egger's bias test yielded *p*=0.203. Sensitivity analysis was performed and the results, as shown in Supporting Figure 8D, were stable and nondifferential, confirming the validity of the methodology.

### 3.8. Results of Subgroup and Regression Analysis

Subgroup and regression analyses were performed to investigate the potential causes of between-study heterogeneity, focusing on the impact of study characteristics on outcome variables and the underlying reasons for heterogeneity. The subgroups analyzed included geographical region (Asia, Europe, North America, and Oceania), treatment duration (≤ 3 months, 4–6 months, and > 6 months), sample size (≤ 50, 51–100, and > 100), age (≤ 44, 45–59, and > 59), and gender (female and male). The results are summarized in Table 2 and the forest plots for the subgroup analyses can be seen in Supporting Figures 9–53. The regression analyses did not reveal any statistically significant effects for these factors (*p* > 0.05). The subgroup analyses revealed statistically significant heterogeneity in body weight within the subgroups defined by treatment duration (≤ 3 months), age (45–59 years), and gender (female and male) (*I*^2^ > 50%, *p* < 0.05). In contrast, the remaining subgroups did not exhibit significant heterogeneity (*I*^2^ < 50%, *p* > 0.05). Similarly, BMI demonstrated statistically significant heterogeneity in the subgroups based on geographical area (Oceania), treatment duration (≤ 3 months and > 6 months), sample size (> 100), age (45–59 years), and gender (female and male) (*I*^2^ > 50%, *p* < 0.05). The other subgroups did not show significant heterogeneity (*I*^2^ < 50%, *p* > 0.05). Heterogeneity was observed in the subgroups based on TC, TG, LDL, and HDL when categorized by region (North America), treatment duration (≤ 3 months), sample size (51–100), age (45–59), and gender (both female and male), as indicated by *I*^2^ > 50% and *p* > 0.05. Conversely, these subgroups did not exhibit heterogeneity in other categories, as evidenced by *I*^2^ > 50% and *p* < 0.05. In addition, HDL displayed statistical heterogeneity within the Asia region subgroup (*I*^2^ > 50%, *p* < 0.05). DBP was statistically heterogeneous in the subgroups defined by sample size (51–100), age (45–59), and gender (female and male), with *I*^2^ > 50% and *p* < 0.05, but did not show statistical heterogeneity in other subgroups (*I*^2^ < 50%, *p* > 0.05). The subgroups based on region (North America), intervention time, sample size (51–100), age (45–59), and gender (both female and male) exhibited statistical heterogeneity with respect to insulin (*I*^2^ > 50%, *p* < 0.05), whereas the remaining subgroups did not demonstrate statistical heterogeneity (*I*^2^ < 50%, *p* > 0.05). Similarly, fat mass showed statistical heterogeneity in the subgroups defined by intervention time (≤ 3 months, > 6 months), sample size (≤ 50), age (45–59), and gender (both female and male) (*I*^2^ > 50%, *p* < 0.05), while other subgroups did not display statistical heterogeneity (*I*^2^ < 50%, *p* > 0.05).

### 3.9. Safety of the 5:2 Diet

All 20 papers included in this study were reviewed for adverse effects. None of the papers reported major adverse events caused by the 5:2 diet. Of note, 7 papers [26, 30–32, 34, 38, 39] documented that the 5:2 diet caused some minor physical and psychological symptoms in participants during fasting. The most common mild adverse reaction was hunger from fasting, but this was usually relieved at the end of the fasting period. Other commonly reported minor physical symptoms were fatigue, feeling cold, headache, dizziness, and constipation. Common mild psychological symptoms included lack of concentration, bad temper, sleep disturbances, and preoccupation with food. However, these symptoms do not require any treatment and can resolve on their own at the end of the trial. Individuals who continue the 5:2 diet on their own were advised to closely monitor their physical and psychological condition and to seek timely adjustments or medical attention if any discomfort arises. The studies also reported positive side effects of the 5:2 diet. Harvie et al. [31] reported that 6% of the participants in the 5:2 diet group would had increased energy and improved health and 32% reported increased self-confidence and positive moods. Thus, based on these results, the 5:2 diet is generally safe and well tolerated.

### 3.10. GRADE Evaluation of Outcome Indicators

The GRADE system evaluates the quality of evidence via the assessment of 5 factors: study limitations, inconsistency, nondirectness, imprecision, and publication bias [44, 45]. The RCTs were initially presented as high-quality evidence; if downgraded one level, as moderate; if downgraded two levels, as low; and if downgraded three levels, as very low. Details of the ratings for each outcome indicator are shown in Supporting Table 3. The results of the ratings for all indicators were presented as moderate- or low-quality evidence.

## 4. Discussion

This review aimed to systematically identify RCTs of 5:2 diet therapy for overweight/obesity and to assess the associated health outcomes in relation to obesity and CVD risk factors. This systematic review and metastudy assessed the effects of the 5:2 diet on body morphology, blood lipids, blood pressure, blood glucose, and heart rate in overweight/obese participants as well as evaluate adverse effects. The results of meta-analysis showed that the 5: 2 diet significantly improved body weight, BMI, waist circumference, body fat percentage, hip circumference, fat mass, fat-free mass, LDL, SBP, and HOMA-IR levels in overweight/obese individuals relative to the control group. However, reductions in visceral fat, TC, TG, HDL, DBP, insulin, FBG, HbA1c, and heart rate were not statistically significant. It is clearly evident that the 5:2 diet caused a significant change in weight and obese appearance of the subjects, as well as lowering LDL and SBP and improving cardiometabolic health. However, except for HOMA-IR, the 5:2 diet did not elicit significant improvements in other glucose metabolism indicators among overweight/obese patients. This may be related to the absence of abnormalities in the baseline glucose metabolism of the study population. Although fasting may cause mild symptoms, none of the articles reported serious adverse effects of the 5:2 diet, suggesting that this diet is safe and feasible. The sensitivity results for this meta-analysis were generally good, indicating good robustness of the conclusions.

The definition of overweight/obesity is geographically and discursively specific. The World Health Organization (WHO) defines overweight as 25 ≤ BMI < 30 kg/m^2^ and obesity as BMI ≥ 30 kg/m^2^ [46]. As Asian populations have higher rates of central obesity and generally higher health risks, the WTO has proposed the following BMI standard for Asian populations: overweight as 23 ≤ BMI < 27.5 kg/m^2^ and obese as BMI ≥ 27.5 kg/m^2^ [47]. However, even in Asia, there are still differences in obesity thresholds between different countries or races. The WHO, therefore, encourages countries to develop their own definitions of obesity. For example, obesity in China is defined as BMI ≥ 27.5 kg/m^2^, or waist circumference > 90 cm for men and > 85 cm for women, while obesity in India is defined as BMI ≥ 25 kg/m^2^ [48, 49]. The European Association for the Study of Obesity proposed a new framework for the diagnosis, staging, and management of obesity in adults in Nature Medicine in July 2024, arguing that BMI alone is not sufficient as a diagnostic criterion for obesity and that the distribution of body fat has a significant impact on health, with the accumulation of abdominal fat also being an important factor [50]. The participants in the study were included based on the different criteria for overweight/obesity in adults from different countries, and all of them had a BMI greater than 24 kg/m^2^. The results of this meta-analysis showed that the 5:2 diet significantly reduced the body weight and BMI in overweight and obese people, as well as reduced the body fat percentage, being especially effective in reducing the accumulation of fat in the abdominal area and buttocks. In addition, the results of this study showed that the 5:2 diet not only reduced fat weight but also had a beneficial effect on fat-free body weight. Therefore, this study proves that the 5:2 diet is a universal, feasible, and effective weight loss method that is applicable internationally.

Fat-free mass is the weight of all body components except fat, with muscle being the main component. Normally, muscle mass should be maintained during weight loss interventions. However, in this study, fat-free mass was significantly reduced after the intervention. This may be due to muscle loss through mechanisms such as energy deficit, metabolic adaptation, inadequate protein intake, or reduced physical activity. The 5:2 diet necessitates that individuals consume a substantially reduced caloric intake on 2 days each week, resulting in an energy deficit due to the greater caloric expenditure compared with intake on fasting days. In instances of persistent or severe energy deficit, the body may catabolize muscle proteins to synthesize glucose, thereby fulfilling the glucose requirements of critical organs such as the brain and erythrocytes [51]. Moreover, adherence to a prolonged 5:2 dietary regimen might induce metabolic adaptations aimed at conserving energy, consequently diminishing the preservation of muscle mass [52]. In addition, the fasting periods associated with this diet could lead to insufficient protein intake, thereby impairing the body's capacity for muscle repair and maintenance. There may also be a reason because some dieters are less active during the intervention due to lack of stamina or reduced exercise capacity. This reduction in activity could subsequently impair the stimulation and maintenance of muscle mass.

In this study, we found that the 5:2 diet led to a significant reduction in body fat percentage; however, it did not result in a substantial improvement in visceral fat. This outcome may be attributed to factors such as fat distribution, individual variability, and the constraints of fasting. Visceral fat is generally more stable than subcutaneous fat and may necessitate a greater and more prolonged energy deficit to achieve a significant reduction. In addition, some individuals exhibit a preferential distribution of fat to the visceral region due to inherent differences [53]. Therefore, it may be difficult to reduce visceral fat content by fasting alone. Furthermore, fasting periods may lead to a reduction in the body's metabolic rate, thereby potentially limiting fat loss. Intermittent fasting could also induce stress-related alterations in corticosteroid hormones, which influence visceral lipolysis [54].

The heterogeneity results indicate that adherence to a 5:2 dietary regimen for a brief duration (≤ 3 months) leads to significant reductions in body weight, BMI, and fat mass, all of which are closely associated with energy expenditure. Moreover, the middle-aged cohort (45–59 years) demonstrated a statistically significant higher success rate in achieving weight and fat loss compared with both younger and older age groups. This phenomenon may be attributed to the transitional phase of insulin resistance commonly experienced by middle-aged individuals, which potentially enhances lipolysis during periods of fasting. Moreover, middle-aged people have a higher willingness to lose weight and are better able to implement a fasting programme, and their short-term compliance will be higher.

The mechanisms of intermittent fasting in weight loss and cardiovascular metabolic health are complex and have now been found to be related to pathways of nutrient perception, oxidative stress, cellular autophagy, circadian rhythms, aging, and inflammation. Humans have evolved adaptations that enable them to survive in a state of food deprivation, leaving glucose and fatty acids in the body in a state of competitive oxidation [55]. In the fed state, glucose is the main source of energy and fat is stored in adipose tissue as TG [56]. In contrast, a negative energy balance occurs in the fasting state, usually 12 h after the cessation of feeding. TG in adipose tissue is converted to fatty acids and glycerol, which are then metabolized for energy. Hepatic glycogen stores are depleted, and the liver converts fatty acids to ketone bodies, which become the main source of energy for many tissues, particularly the brain [57–59]. Obesity has been shown to be associated with inflammation in several organ systems; in this context, the 5:2 diet reduces markers of oxidative stress and increases the levels of antioxidant enzymes that may inhibit inflammation [60, 61]. In addition, the 5:2 diet promotes the rebuilding of the gut microbiota, which is significantly associated with weight loss [62]. In addition, the 5:2 diet regulates the balance between appetite and energy expenditure by modulating central and peripheral clock genes [63, 64]. By constantly switching between dieting and eating, the 5:2 diet allows cells and organs to adapt to this bioenergetic challenge through a variety of pathways, which improves health, increases resistance to disease and helps to slow aging and prolong lifespan [65].

However, the 5:2 diet is not suitable for all individuals and must be implemented under professional guidance. The process of intermittent fasting can produce adverse reactions such as fatigue, dizziness, and weakness. Although the adverse effects are mild and the present study demonstrated no serious adverse effects of the 5:2 diet, fasting can be harmful to specific groups of people such as the elderly, frail, or children. For patients who need long-term medication such as for diabetes or hypertension, intermittent fasting should be performed after assessment by a medical professional, close monitoring of blood glucose and blood pressure, and timely adjustment of hypoglycemic and antihypertensive medication to avoid hypoglycemia or hypotension.

The 5:2 diet is now widely implemented for weight loss in the obese population. Clinical trials have confirmed the reliability of this diet, while several systematic reviews and meta-analyses have been published on the benefits of intermittent fasting to reduce body weight and improve cardiometabolic parameters [66, 67]. However, these meta-analyses summarize all types of intermittent fasting. There is currently a lack of published literature that focuses on the 5:2 diet and comprehensively evaluates weight loss and CVD risk factors in overweight/obese individuals. In this study, we included the most recent clinical study data, conducted an updated review, and presented the most recent findings. Our results were consistent with most of the studies. Second, we performed Egger's test and sensitivity analyses to provide a more comprehensive categorization of the level of evidence, and the results of this study were found to be robust and without significant publication bias. In addition, the Cochrane risk of bias assessment of the RCTs of this study was not of low quality. Finally, GRADE ratings assessed the quality of evidence of this study as moderate- or low-quality evidence.

This systematic review and meta-analysis has some limitations. There was heterogeneity in some of the indicators in this meta-analysis, which might be related to the timing of the intervention, the location of the intervention, and the subjects' own characteristics (e.g., age, education level, and economic status). Moreover, some studies did not detail allocation concealment and blinding. In addition, the present study included trials in which—although the interventions ranging from 1 to 12 months—the adherence of fasting individuals was not considered, thus failing to evaluate long-term adherence to the 5:2 diet. Finally, although it is known that weight is prone to rebound, most of the current studies are short- to medium-term studies with a lack of long-term follow-up data. Future studies should, therefore, focus on long-term interventions.

## 5. Conclusions

In conclusion, this study confirmed that the 5:2 diet facilitates weight loss in overweight/obese individuals and indicated that it has positive effects on blood lipids and blood pressure, which can mitigate the risk factors for CVD. More high-quality and long-term studies are anticipated to confirm the results of this meta-analysis, with a view to providing references for the clinical selection of intermittent fasting patterns.

## Figures and Tables

**Table 1:** General characteristics of included studies.

First author, year	Country	Participant	Intervention diet and control diet	Treatment duration (month) (m)	Sample size (C/T)	Age (year)	Gender (female/male)	Outcomes
Carter 2016	Australia	Overweight or obese participants with type 2 diabetes	T: A 1670–2500 kJ/day for 2 days each week, with the remaining 5 days as habitual eating C: A 7-day continuous energy restriction diet of 5000–6500 kJ/day	3	32/31	C:62 ± 9.1 T:61 ± 7.5	C:16/16 T:17/14	①②⑤⑦⑧⑱

Carter 2018	Australia	Adults (> 18 years of age) with type 2 diabetes who were overweight or obese (BMI > 27 kg/m^2^)	T: A diet of 500–600 kcal/d for 2 days of the week and followed their usual diet for the other 5 days C: A diet of 1200–1500 kcal/day	12	67/70	C:61 ± 9.2 T:61 ± 9.0	C:38/29 T:39/31	①②⑤⑥⑦⑧⑱

Conley 2017	Australia	Males aged 55–75 years with BMI ≥ 30 kg/m^2^ and weight stable	T: Two nonconsecutive days of 2460 kJ (600 calories) and 5 days of ad libitum eating per week	3	12/11	C:67.1 ± 3.9 T:68 ± 2.7	C:0/12 T:0/11	①③⑨⑩⑪⑫⑬⑭⑰⑳

Fudla 2021	Indonesia	Obese male students aged 18–25	T: Fasted 2 days per week C: Standard energy-reduced diet	1	20/20	C:19.01 ± 0.93 T:18.95 ± 0.68	C:0/20 T:0/20	②

Gray 2021	Australia	Overweight females ≥ 18 years	T: 2 nonconsecutive days each week follow a very low–energy diet of 500 kcal (2100 kJ) per day C: Follow a diet of 1500 kcal (6000 kJ) per day	12	30/32	C:40.2 ± 9.2 T:39.3 ± 8.9	C:30/0 T:32/0	①②⑮⑯⑰⑱

Guo 2021	China	30–50 years with central obesity circumference ≥ 90 cm in males or ≥ 80 cm in females	T: A 75% of energy restriction for two nonconsecutive days a week, and ad libitum diet the other 5 days C: A routine diet without dietary instructions	2	18/21	C:42.7 ± 4.1 T:40.2 ± 5.7	C:7/11 T:11/10	①②③④⑥⑦⑧⑨⑩⑪⑫⑬⑭⑮⑯⑰⑲

Hajek 2021	UK	Adults with obesity BMI ≥ 30 kg/m^2^ (or 28 ≥ kg/m^2^, with co-morbidities)	T: Restricting caloric intake to 500–600 kcal on two non-consecutive days a week, no formal energy restrictions on the remaining days C: Standard energy-reduced diet	12	48/45	C:47 ± 13 T:47 ± 13	C:31/17 T:30/15	①

Harvie 2011	UK	Overweight or obese premenopausal women aged 30–45 years	T: ∼2266 kJ/day for 2 days/week C: ∼6276 kJ/day for 7 days/week	6	47/42	C:40.0 ± 3.9 T:40.1 ± 4.1	C:47/0 T:42/0	①③④⑤⑦⑧⑨⑩⑪⑫⑬⑭⑮⑯⑰⑲⑳

Harvie 2013	UK	Women BMI was 24–45 kg/m^2^ and/or body fat was > 30% of total weight between 20 and 69 years of age	T: 70% energy restriction on two consecutive days and consume a euenergetic Mediterranean-type diet on the remaining 5 days C: A daily energy-restricted Mediterranean-type diet	3	40/37	C: 47.9 ± 7.7 T: 45.6 ± 8.3	C:40/0 T:37/0	①③④⑦⑧⑨⑩⑪⑫⑬⑮⑯⑰⑱⑲⑳

He 2021	China	BMI ranging from 24 to 40 kg/m^2^ aged 18–70 years	T: A very-low-energy diet of 500–600 kcal for 2 days of the week along with their usual diet for the other 5 days C: A daily 25% restriction based on a Mediterranean-type diet	6	85/88	C:50.7 ± 8.7 T:50.2 ± 8.9	C:50/35 T:50/38	①⑤⑦⑨⑩⑪⑫⑬⑭⑱⑲⑳

Headland 2019	Australia	Overweight and obese adults, ages 18–72 years	T: 5 days of habitual intake and 2 very low energy diet days each week C: 4200 kJ/day for women and 5040 kJ/day for men	12	53/49	C:51.7 ± 13.0 T:47.5 ± 14.5	C:43/10 T:41/8	①②⑦⑧⑨⑩⑪⑫⑰

Hirsh 2019	USA	Overweight males and females, aged 21–65 years with a BMI between 25 and 29.9 kg/m^2^	T: Two consecutive days of each week with 730 kcal/d, 3050 kJ/d on the remaining 5 days no specific dietary recommendations C: Follow habitual diet without any restriction	3	12/10	C:39 ± 10.7 T:43.4 ± 13.0	C:7/5 T:8/2	①⑨⑩⑪⑫⑬⑭⑮⑯⑲⑳

Keenan 2022	Australia	Individuals with a BMI of 22–35 kg/m^2^ aged between 18 and 35 years	T: ∼20% of energy requirements twice weekly, 100% energy requirements on the remaining 5 days C: Consume 80% of energy requirements daily	3	17/17	C:23.2 ± 3.9 T:24.7 ± 4.8	C:8/9 T:9/8	⑨⑩⑪⑫⑮⑯⑰

Panizza 2019	USA	Aged 35–55, BMI 25–40 kg/m^2^, VAT ≥ 90 cm for men and 80 ≥ cm for women	T: Two consecutive days with 70% energy restriction and 5 days euenergetic Mediterranean-type diet C: Euenergetic dietary approaches to stop hypertension diet	3	30/30	C:46.2 ± 5.4 T:48.4 ± 4.7	C:21/9 T:21/9	①②③④⑤⑥⑦⑧⑨⑩⑪⑫⑬⑭⑯⑰

Pinto 2020	UK	Adults with a waist circumference > 90 cm and > 80 cm for men and women respectively	T: 48 h 600 kcal/d followed by 5-day healthy eating advice C: Consume a nutritionally balanced Mediterranean diet incorporating a daily 500 kcal	1	22/21	C:56 ± 8 T:50 ± 12	C:16/6 T:15/6	①②③⑤⑩⑬⑭⑯⑰⑱⑲

Schübel 2018	Germany	Overweight and obese nonsmokers 25 ≤ BMI < 40 kg/m^2^	T: 5 d without energy restriction and 2 d with 75% energy deficit C: Daily energy deficit ∼20%	3	49/49	C:50.5 ± 8.0 T:49.4 ± 9.0	C:24/25 T:24/25	①⑨⑩⑪⑫⑮⑯⑰⑲⑳

Sundfør 2018	Norway	Aged 21–70 years with BMI 30–45 kg/m^2^	T: Consume 400/600 kcal on two non-consecutive days and consume as usual on the remaining 5 days a week C: Reduce energy intake evenly 7 days a week	6	58/54	C:47.5 ± 11.6 T:49.9 ± 10.1	C:30/28 T:26/28	①②③④⑨⑩⑪⑫⑬⑭⑰⑱⑲⑳

Varkaneh 2022	Romania	BMI 25–40 kg/m^2^, age between 18 and 50 years	T: 5 days a week of normal food intake and 2 consecutive fasting days a week C: Standard energy-reduced diet	3	23/21	C:44.17 ± 4.9 T:46.42 ± 13.35	C:8/15 T:9/12	①②③⑦⑧⑨⑩⑪⑫⑮⑯⑰

Witjaksono 2022	Indonesia	Men aged 19–59 years, with a BMI ≥ 25 kg/m^2^, a waist circumference ≥ 90 cm	T: Two nonconsecutive days of fasting for ±14 h, and 5 days of ad libitum eating per week C: Standard energy-reduced diet	2	25/25	C:30 ± 8.0 T:32 ± 8.3	C:0/25 T:0/25	①②⑤⑥⑧

Xu 2022	China	Overweight (BMI ≥ 24 kg/m^2^) or obese (BMI ≥ 28 kg/m^2^) aged 18–30 years	T: Consumed 30% of daily recommended energy intake on 2 nonconsecutive days and consumed food ad libitum for the other 5 days C: Consume a daily hyperenergetic diet of 70% of their estimated energy requirements	1	16/16	—	C:9/7 T:10/6	②③④⑤

*Note:* C = control group, T = treatment group, ① = body weight, ② = body mass index, ③ = waist circumference, ④ = hip circumference, ⑤ = body fat percentage, ⑥ = visceral fat, ⑦ = fat mass, ⑧ = fat free mass, ⑨ = total cholesterol, ⑩ = triglyceride, ⑪ = low density lipoprotein, ⑫ = high density lipoprotein, ⑬ = systolic blood pressure, ⑭ = diastolic blood pressure, ⑮ = HOMA-IR, ⑯ = insulin, ⑰ = fasting blood glucose, ⑱ = HbA1c, ⑲ = heart rate, ⑳ = adverse effect.

**Table 2:** Subgroup analyses and regression analyses.

Indicator/Subgroup	Classification	Effect size	*p*	Heterogeneity test result		Regression analysis result	
				*I* ^2^ (%)	*p*	Coefficient	*p*
Body weight		−1.88	0.04	62	0.0003		
Area	Asia	0.27	0.87	0	0.92	−0.78	0.36
	Europe	−1.78	0.15	30	0.20		
	North America	−4.39	< 0.00001	0	0.92		
	Oceania	−1.06	0.55	0	0.46		
Treatment duration	≤ 3 months	−2.56	0.03	76	< 0.0001	0.01	0.51
	4–6 months	−0.95	0.58	0	0.53		
	> 6 months	−0.01	1.00	0	0.59		
Sample size	≤ 50	−0.44	0.34	0	0.43	0.16	0.51
	51–100	−4.20	< 0.00001	0	0.46		
	> 100	0.01	1.00	0	0.60		
Age	≤ 44	−1.13	0.51	0	0.67	−1.22	0.45
	45–59	−1.77	0.14	79	< 0.00001		
	> 59	−3.69	0.12	0	0.68		
Gender	Female	−4.56	0.06	18	0.30	1.40	0.28
	Male	−2.36	0.56	31	0.23		
	Female and male	−1.45	0.16	70	0.0001		

BMI		−0.85	0.01	72	< 0.0001		
Area	Asia	−1.32	0.02	0	0.85	−0.07	0.85
	Europe	−0.14	0.38	0	0.42		
	North America	−1.30	< 0.00001	—	—		
	Oceania	−1.05	0.37	66	0.03		
Treatment duration	≤ 3 months	−1.00	0.01	77	< 0.0001	0.03	0.73
	4–6 months	0.00	1.00	—	—		
	> 6 months	−0.52	0.72	72	0.03		
Sample size	≤ 50	−0.78	0.05	32	0.18	0.00	0.85
	51–100	−1.29	< 0.00001	0	0.42		
	> 100	−0.54	0.63	72	0.03		
Age	≤ 44	−0.50	0.58	0	0.93	−1.14	0.14
	45–59	−0.64	0.11	85	< 0.00001		
	> 59	−2.76	0.002	0	0.92		
Gender	Female	0.60	0.80	—	—	0.14	0.87
	Male	−1.64	0.10	0	0.63		
	Female and male	−0.80	0.04	81	< 0.00001		

Total cholesterol		−0.23	0.11	74	< 0.0001		
Area	Asia	0.02	0.86	0	0.72	−0.11	0.55
	Europe	−0.13	0.20	0	0.67		
	North America	−1.01	0.35	94	< 0.0001		
	Oceania	−0.11	0.49	4	0.35		
Treatment duration	≤ 3 months	−0.35	0.16	81	< 0.00001	0.03	0.67
	4–6 months	−0.01	0.89	0	0.65		
	> 6 months	−0.17	0.39	—	—		
Sample size	≤ 50	−0.07	0.65	0	0.44	0.00	0.71
	51–100	−0.54	0.18	91	< 0.00001		
	> 100	−0.09	0.39	0	0.74		
Age	≤ 44	0.15	0.31	0	0.98	−0.44	0.17
	45–59	−0.40	0.04	83	< 0.00001		
	> 59	−0.46	0.28	—	—		
Gender	Female	−0.01	0.96	0	0.37	−0.11	0.66
	Male	−0.46	0.28	—	—		
	Female and male	−0.27	0.14	80	< 0.00001		

Triglycerides		−0.25	0.13	80	< 0.00001		
Area	Asia	−0.05	0.74	0	0.90	−0.15	0.49
	Europe	−0.02	0.86	26	0.24		
	North America	−1.50	0.16	93	0.0001		
	Oceania	−0.02	0.91	33	0.23		
Treatment duration	≤ 3 months	−0.45	0.08	84	< 0.00001	0.07	0.39
	4–6 months	0.04	0.71	0	0.54		
	> 6 months	0.22	0.27	—	—		
Sample size	≤ 50	−0.12	0.41	0	0.50	0.00	0.43
	51–100	−0.70	0.13	93	< 0.00001		
	> 100	0.10	0.35	0	0.42		
Age	≤ 44	−0.07	0.64	0	0.85	−0.21	0.59
	45–59	−0.29	0.21	88	< 0.00001		
	> 59	−0.56	0.19	—	—		
Gender	Female	−0.20	0.34	42	0.19	−0.00	1.00
	Male	−0.56	0.19	—	—		
	Female and male	−0.24	0.24	84	< 0.00001		

Low-density lipoprotein		−0.24	0.02	54	0.01		
Area	Asia	−0.18	0.18	0	0.64	−0.03	0.78
	Europe	−0.13	0.18	0	0.95		
	North America	−0.87	0.23	87	0.005		
	Oceania	−0.12	0.44	0	0.61		
Treatment duration	≤ 3 months	−0.32	0.09	68	0.003	0.02	0.64
	4–6 months	−0.14	0.19	0	0.73		
	> 6 months	−0.19	0.34	—	—		
Sample size	≤ 50	−0.13	0.41	0	0.87	0.00	0.74
	51–100	−0.43	0.16	86	< 0.0001		
	> 100	−0.18	0.07	0	0.96		
Age	≤ 44	−0.05	0.75	0	0.75	−0.20	0.42
	45–59	−0.32	0.03	71	0.002		
	> 59	−0.27	0.51	—	—		
Gender	Female	−0.04	0.78	0	0.77	−0.12	0.46
	Male	−0.27	0.51	—	—		
	Female and male	−0.29	0.03	64	0.004		

High-density lipoprotein		0.27	0.06	75	< 0.00001		
Area	Asia	−0.07	0.79	60	0.12	0.11	0.51
	Europe	0.14	0.15	0	0.69		
	North America	1.29	0.07	86	0.009		
	Oceania	0.06	0.69	0	0.75		
Treatment duration	≤ 3 months	0.48	0.03	76	0.0002	−0.07	0.25
	4–6 months	−0.09	0.47	26	0.26		
	> 6 months	0.00	1.00	—	—		
Sample size	≤ 50	0.35	0.03	0	0.85	−0.01	0.15
	51–100	0.53	0.16	90	< 0.00001		
	> 100	−0.09	0.47	27	0.25		
Age	≤ 44	0.18	0.23	0	0.65	−0.04	0.91
	45–59	0.33	0.14	86	< 0.00001		
	> 59	0.00	1.00	—	—		
Gender	Female	0.04	0.80	0	0.79	0.18	0.43
	Male	0.00	1.00	—	—		
	Female and male	0.36	0.06	82	< 0.00001		

Diastolic blood pressure		−1.71	0.22	77	< 0.0001		
Area	Asia	−1.46	0.52	44	0.18	−1.78	0.19
	Europe	0.13	0.91	0	0.43		
	North America	−5.26	< 0.00001	0	0.58		
	Oceania	−0.60	0.93	—	—		
Treatment duration	≤ 3 months	−3.71	0.003	48	0.10	0.85	0.20
	4–6 months	0.64	0.57	0	0.62		
Sample size	≤ 50	−2.00	0.11	0	0.67	0.03	0.26
	51–100	−1.64	0.68	91	0.001		
	> 100	0.07	0.96	0	0.90		
Age	≤ 44	−1.57	0.53	56	0.10	−0.22	0.93
	45–59	−1.84	0.30	86	< 0.0001		
	> 59	−0.60	0.93	—	—		
Gender	Female	2.70	0.26	—	—	−2.58	0.18
	Male	−0.60	0.93	—	—		
	Female and male	−2.44	0.08	77	0.0007		

Insulin		−0.34	0.06	76	< 0.0001		
Area	Asia	0.37	0.26	—	—	−0.24	0.31
	Europe	−0.22	0.09	26	0.25		
	North America	−1.33	0.03	81	0.02		
	Oceania	−0.26	0.29	25	0.25		
Treatment duration	≤ 3 months	−0.33	0.17	82	< 0.00001	−0.03	0.67
	4–6 months	−0.37	0.09	—	—		
	> 6 months	−0.45	0.08	—	—		
Sample size	≤ 50	0.06	0.69	0	0.42	−0.00	0.64
	51–100	−0.65	0.01	83	< 0.0001		
Age	≤ 44	−0.22	0.19	35	0.19	−0.26	0.56
	45–59	−0.47	0.15	87	< 0.00001		
Gender	Female	−0.46	0.0007	0	0.81	0.09	0.72
	Female and male	−0.29	0.31	84	< 0.00001		

Fat mass		−1.82	0.02	55	0.02		
Area	Asia	0.80	0.70	62	0.11	−0.11	0.91
	Europe	−4.86	0.0001	0	0.50		
	North America	−1.80	< 0.00001	—	—		
	Oceania	−0.97	0.64	59	0.09		
Treatment duration	≤ 3 months	−2.36	0.09	67	0.02	0.14	0.66
	4–6 months	−1.27	0.27	0	0.54		
	> 6 months	−0.60	0.84	79	0.03		
Sample size	≤ 50	−0.93	0.82	87	0.006	0.00	0.96
	51–100	−2.67	0.009	34	0.21		
	> 100	−0.85	0.58	58	0.09		
Age	≤ 44	0.43	0.88	68	0.08	−1.72	0.33
	45–59	−2.12	0.05	65	0.02		
	> 59	−3.13	0.04	0	0.64		
Gender	Female	−4.67	0.01	—	—	1.71	0.20
	Female and male	−1.27	0.14	54	0.04		

## Data Availability

The original contributions presented in the study are included in the article/Supporting Information, and further inquiries can be directed to the corresponding author.

## Supporting Information

Supporting Figure 1: Funnel plots of blood lipids. (A) TC; (B) TG; (C) LDL; and (D) HDL.

Supporting Figure 2: Sensitivity plots of blood lipids. (A) TC; (B) TG; (C) LDL; and (D) HDL.

Supporting Figure 3: Funnel plots of blood pressure. (A) SBP; (B) DBP; and (C) Heart rate.

Supporting Figure 4: Sensitivity plots of blood pressure. (A) SBP; (B) DBP; and (C) Heart rate.

Supporting Figure 5: Funnel plots of glucose metabolism. (A) HOMA-IR; (B) Insulin; (C) FBG; and (D) HbA1c.

Supporting Figure 6: Sensitivity plots of glucose metabolism. (A) HOMA-IR; (B) Insulin; (C) FBG; and (D) HbA1c.

Supporting Figure 7: Funnel plots of other secondary indicators. (A) Hip circumference; (B) Visceral fat; (C) Fat mass; and (D) Fat-free mass.

Supporting Figure 8: Sensitivity plots of other secondary indicators. (A) Hip circumference; (B) Visceral fat; (C) Fat mass; and (D) Fat-free mass.

Supporting Figure 9: Forest map of body weight analyzed in subgroups by geographic location.

Supporting Figure 10: Forest map of body weight analyzed in subgroups by treatment duration.

Supporting Figure 11: Forest map of body weight analyzed in subgroups by sample size.

Supporting Figure 12: Forest map of body weight analyzed in subgroups by age.

Supporting Figure 13: Forest map of body weight analyzed in subgroups by gender.

Supporting Figure 14: Forest map of BMI analyzed in subgroups by geographic location.

Supporting Figure 15: Forest map of BMI analyzed in subgroups by treatment duration.

Supporting Figure 16: Forest map of BMI analyzed in subgroups by sample size.

Supporting Figure 17: Forest map of BMI analyzed in subgroups by age.

Supporting Figure 18: Forest map of BMI analyzed in subgroups by gender.

Supporting Figure 19: Forest map of TC analyzed in subgroups by geographic location.

Supporting Figure 20: Forest map of TC analyzed in subgroups by treatment duration.

Supporting Figure 21: Forest map of TC analyzed in subgroups by sample size.

Supporting Figure 22: Forest map of TC analyzed in subgroups by age.

Supporting Figure 23: Forest map of TC analyzed in subgroups by gender.

Supporting Figure 24: Forest map of TG analyzed in subgroups by geographic location.

Supporting Figure 25: Forest map of TG analyzed in subgroups by treatment duration.

Supporting Figure 26: Forest map of TG analyzed in subgroups by sample size.

Supporting Figure 27: Forest map of TG analyzed in subgroups by age.

Supporting Figure 28: Forest map of TG analyzed in subgroups by gender.

Supporting Figure 29: Forest map of LDL analyzed in subgroups by geographic location.

Supporting Figure 30: Forest map of LDL analyzed in subgroups by treatment duration.

Supporting Figure 31: Forest map of LDL analyzed in subgroups by sample size.

Supporting Figure 32: Forest map of LDL analyzed in subgroups by age.

Supporting Figure 33: Forest map of LDL analyzed in subgroups by gender.

Supporting Figure 34: Forest map of HDL analyzed in subgroups by geographic location.

Supporting Figure 35: Forest map of HDL analyzed in subgroups by treatment duration.

Supporting Figure 36: Forest map of HDL analyzed in subgroups by sample size.

Supporting Figure 37: Forest map of HDL analyzed in subgroups by age.

Supporting Figure 38: Forest map of HDL analyzed in subgroups by gender.

Supporting Figure 39: Forest map of DBP analyzed in subgroups by geographic location.

Supporting Figure 40: Forest map of DBP analyzed in subgroups by treatment duration.

Supporting Figure 41: Forest map of DBP analyzed in subgroups by sample size.

Supporting Figure 42: Forest map of DBP analyzed in subgroups by age.

Supporting Figure 43: Forest map of DBP analyzed in subgroups by gender.

Supporting Figure 44: Forest map of insulin analyzed in subgroups by geographic location.

Supporting Figure 45: Forest map of insulin analyzed in subgroups by treatment duration.

Supporting Figure 46: Forest map of insulin analyzed in subgroups by sample size.

Supporting Figure 47: Forest map of insulin analyzed in subgroups by age.

Supporting Figure 48: Forest map of insulin analyzed in subgroups by gender.

Supporting Figure 49: Forest map of fat mass analyzed in subgroups by geographic location.

Supporting Figure 50: Forest map of fat mass analyzed in subgroups by treatment duration.

Supporting Figure 51: Forest map of fat mass analyzed in subgroups by sample size.

Supporting Figure 52: Forest map of fat mass analyzed in subgroups by age.

Supporting Figure 53: Forest map of fat mass analyzed in subgroups by gender.

Supporting Table 1: PRISMA checklist.

Supporting Table 2: Search strategy in PubMed.

Supporting Table 3: GRADE quality of evidence evaluation form.

## References

[1] Perdomo C. M., Cohen R. V., Sumithran P., Clément K., Frühbeck G. (2023). Contemporary Medical, Device, and Surgical Therapies for Obesity in Adults. *The Lancet*.

[2] Elagizi A., Kachur S., Carbone S., Lavie C. J., Blair S. N. (2020). A Review of Obesity, Physical Activity, and Cardiovascular Disease. *Current Obesity Reports*.

[3] Fan K., Lv F., Li H., Meng F., Wang T., Zhou Y. (2023). Trends in Obesity and Severe Obesity Prevalence in the United States From 1999 to 2018. *American Journal of Human Biology: The Official Journal of the Human Biology Council*.

[4] Piché M. E., Tchernof A., Després J. P. (2020). Obesity Phenotypes, Diabetes, and Cardiovascular Diseases. *Circulation Research*.

[5] Tutor A. W., Lavie C. J., Kachur S., Milani R. V., Ventura H. O. (2023). Updates on Obesity and the Obesity Paradox in Cardiovascular Diseases. *Progress in Cardiovascular Diseases*.

[6] The Gbd 2015 Obesity Collaborators, Afshin A., Forouzanfar M. H. (2017). Health Effects of Overweight and Obesity in 195 Countries Over 25 Years. *New England Journal of Medicine*.

[7] Forouhi N. G. (2023). Embracing Complexity: Making Sense of Diet, Nutrition, Obesity and Type 2 Diabetes. *Diabetologia*.

[8] Grosso G. (2021). Intermittent Fasting: Promising Premises or Broken Promises?. *International Journal of Food Sciences & Nutrition*.

[9] Minciuna I., Gallage S., Heikenwalder M., Zelber-Sagi S., Dufour J. F. (2023). Intermittent Fasting-The Future Treatment in NASH Patients?. *Hepatology*.

[10] Elortegui Pascual P., Rolands M. R., Eldridge A. L. (2023). A Meta-Analysis Comparing the Effectiveness of Alternate Day Fasting, the 5:2 Diet, and Time-Restricted Eating for Weight Loss. *Obesity*.

[11] Duregon E., Pomatto-Watson L. C. D. D., Bernier M., Price N. L., de Cabo R. (2021). Intermittent Fasting: From Calories to Time Restriction. *GeroScience*.

[12] Templeman I., Gonzalez J. T., Thompson D., Betts J. A. (2020). The Role of Intermittent Fasting and Meal Timing in Weight Management and Metabolic Health. *Proceedings of the Nutrition Society*.

[13] Guo L., Xi Y., Jin W. (2024). A 5:2 Intermittent Fasting Meal Replacement Diet and Glycemic Control for Adults With Diabetes: The EARLY Randomized Clinical Trial. *JAMA Network Open*.

[14] Gallage S., Ali A., Barragan Avila J. E. (2024). A 5:2 Intermittent Fasting Regimen Ameliorates NASH and Fibrosis and Blunts HCC Development via Hepatic PPARα and PCK1. *Cell Metabolism*.

[15] Mattson M. P., Longo V. D., Harvie M. (2017). Impact of Intermittent Fasting on Health and Disease Processes. *Ageing Research Reviews*.

[16] Varady K. A., Cienfuegos S., Ezpeleta M., Gabel K. (2021). Cardiometabolic Benefits of Intermittent Fasting. *Annual Review of Nutrition*.

[17] Strilbytska O., Klishch S., Storey K. B., Koliada A., Lushchak O. (2024). Intermittent Fasting and Longevity: From Animal Models to Implication for Humans. *Ageing Research Reviews*.

[18] Page M. J., McKenzie J. E., Bossuyt P. M. (2021). The PRISMA 2020 Statement: An Updated Guideline for Reporting Systematic Reviews. *BMJ*.

[19] Gotschall T. (2021). EndNote 20 Desktop Version. *Journal of the Medical Library Association: JMLA*.

[20] Barcot O., Ivanda M., Buljan I., Pieper D., Puljak L. (2021). Enhanced Access to Recommendations From the Cochrane Handbook for Improving Authors’ Judgments About Risk of Bias: A Randomized Controlled Trial. *Research Synthesis Methods*.

[21] Cumpston M. S., McKenzie J. E., Welch V. A., Brennan S. E. (2022). Strengthening Systematic Reviews in Public Health: Guidance in the *Cochrane Handbook For Systematic Reviews of Interventions*, 2nd Edition. *Journal of Public Health*.

[22] Oliveira-Cortez A., Rodrigues Ferreira I., Luíza Nunes Abreu C., de Oliveira Bosco Y., Kümmel Duarte C., Nogueira Cortez D. (2023). Incidence of the First Diabetic Foot Ulcer: A Systematic Review and Meta-Analysis. *Diabetes Research and Clinical Practice*.

[23] Shim S., Yoon B. H., Shin I. S., Bae J. M. (2017). Network Meta-Analysis: Application and Practice Using Stata. *Epidemiology and Health*.

[24] Carter S., Clifton P. M., Keogh J. B. (2016). The Effects of Intermittent Compared to Continuous Energy Restriction on Glycaemic Control in Type 2 Diabetes; a Pragmatic Pilot Trial. *Diabetes Research and Clinical Practice*.

[25] Carter S., Clifton P. M., Keogh J. B. (2018). Effect of Intermittent Compared With Continuous Energy Restricted Diet on Glycemic Control in Patients With Type 2 Diabetes: A Randomized Noninferiority Trial. *JAMA Network Open*.

[26] Conley M., Le Fevre L., Haywood C., Proietto J. (2018). Is Two Days of Intermittent Energy Restriction Per Week a Feasible Weight Loss Approach in Obese Males? A Randomised Pilot Study. *Nutrition and Dietetics*.

[27] Gray K. L., Clifton P. M., Keogh J. B. (2021). The Effect of Intermittent Energy Restriction on Weight Loss and Diabetes Risk Markers in Women With a History of Gestational Diabetes: A 12-Month Randomized Control Trial. *The American Journal of Clinical Nutrition*.

[28] Guo Y., Luo S., Ye Y., Yin S., Fan J., Xia M. (2021). Intermittent Fasting Improves Cardiometabolic Risk Factors and Alters Gut Microbiota in Metabolic Syndrome Patients. *Journal of Clinical Endocrinology and Metabolism*.

[29] Hajek P., Przulj D., Pesola F. (2021). A Randomised Controlled Trial of the 5:2 Diet. *PLoS One*.

[30] Harvie M., Wright C., Pegington M. (2013). The Effect of Intermittent Energy and Carbohydrate Restriction V. Daily Energy Restriction on Weight Loss and Metabolic Disease Risk Markers in Overweight Women. *British Journal of Nutrition*.

[31] Harvie M. N., Pegington M., Mattson M. P. (2011). The Effects of Intermittent or Continuous Energy Restriction on Weight Loss and Metabolic Disease Risk Markers: A Randomized Trial in Young Overweight Women. *International Journal of Obesity*.

[32] He C. J., Fei Y. P., Zhu C. Y. (2021). Effects of Intermittent Compared With Continuous Energy Restriction on Blood Pressure Control in Overweight and Obese Patients With Hypertension. *Frontiers in Cardiovascular Medicine*.

[33] Headland M. L., Clifton P. M., Keogh J. B. (2019). Effect of Intermittent Compared to Continuous Energy Restriction on Weight Loss and Weight Maintenance After 12 Months in Healthy Overweight or Obese Adults. *International Journal of Obesity*.

[34] Hirsh S. P., Pons M., Joyal S. V., Swick A. G. (2019). Avoiding Holiday Seasonal Weight Gain With Nutrient-Supported Intermittent Energy Restriction: A Pilot Study. *Journal of Nutrition Sciences*.

[35] Kord Varkaneh H., Salehi Sahlabadi A., Găman M. A. (2022). Effects of the 5:2 Intermittent Fasting Diet on Non-Alcoholic Fatty Liver Disease: A Randomized Controlled Trial. *Frontiers in Nutrition*.

[36] Panizza C. E., Lim U., Yonemori K. M. (2019). Effects of Intermittent Energy Restriction Combined With a Mediterranean Diet on Reducing Visceral Adiposity: A Randomized Active Comparator Pilot Study. *Nutrients*.

[37] Pinto A. M., Bordoli C., Buckner L. P. (2020). Intermittent Energy Restriction Is Comparable to Continuous Energy Restriction for Cardiometabolic Health in Adults With Central Obesity: A Randomized Controlled Trial; the Met-IER Study. *Clinical Nutrition*.

[38] Schübel R., Nattenmüller J., Sookthai D. (2018). Effects of Intermittent and Continuous Calorie Restriction on Body Weight and Metabolism over 50 Wk: A Randomized Controlled Trial. *The American Journal of Clinical Nutrition*.

[39] Sundfør T. M., Svendsen M., Tonstad S. (2018). Effect of Intermittent Versus Continuous Energy Restriction on Weight Loss, Maintenance and Cardiometabolic Risk: A Randomized 1-Year Trial. *Nutrition, Metabolism, and Cardiovascular Diseases*.

[40] Witjaksono F., Prafiantini E., Rahmawati A. (2022). Effect of Intermittent Fasting 5:2 on Body Composition and Nutritional Intake Among Employees With Obesity in Jakarta: A Randomized Clinical Trial. *BMC Research Notes*.

[41] Fudla H., Mudjihartini N., Khusun H. (2021). Effect of Four Weeks of 5:2 Intermittent Fasting on Energy Intake and Body Mass Index Among Obese Male Students Aged 18-25. *Obesity Medicine*.

[42] Xu R., Cao Y. X., Chen Y. T., Jia Y. Q. (2022). Differential Effects of Intermittent Energy Restriction vs. Continuous Energy Restriction Combined High-Intensity Interval Training on Overweight/Obese Adults: A Randomized Controlled Trial. *Frontiers in Nutrition*.

[43] Keenan S., Cooke M. B., Chen W. S., Wu S., Belski R. (2022). The Effects of Intermittent Fasting and Continuous Energy Restriction with Exercise on Cardiometabolic Biomarkers, Dietary Compliance, and Perceived Hunger and Mood: Secondary Outcomes of a Randomised, Controlled Trial. *Nutrients*.

[44] Guyatt G. H., Oxman A. D., Vist G. E. (2008). GRADE: an Emerging Consensus on Rating Quality of Evidence and Strength of Recommendations. *BMJ*.

[45] Niu S., Liu Y., Li D. (2023). Effect of Indocyanine Green Near-Infrared Light Imaging Technique Guided Lymph Node Dissection on Short-Term Clinical Efficacy of Minimally Invasive Radical Gastric Cancer Surgery: A Meta-Analysis. *Frontiers in Oncology*.

[46] Blüher M. (2019). Obesity: Global Epidemiology and Pathogenesis. *Nature Reviews Endocrinology*.

[47] Who Expert Consultation (2004). Appropriate Body-Mass Index for Asian Populations and its Implications for Policy and Intervention Strategies. *Lancet (London, England)*.

[48] Chen K., Shen Z., Gu W. (2023). Prevalence of Obesity and Associated Complications in China: A Cross-Sectional, Real-World Study in 15.8 Million Adults. *Diabetes, Obesity and Metabolism*.

[49] Kalra S., Kapoor N., Verma M. (2023). Defining and Diagnosing Obesity in India: A Call for Advocacy and Action. *Journal of Obesity*.

[50] Busetto L., Dicker D., Frühbeck G. (2024). A New Framework for the Diagnosis, Staging and Management of Obesity in Adults. *Nature Medicine*.

[51] Tam J., Fukumura D., Jain R. K. (2009). A Mathematical Model of Murine Metabolic Regulation by Leptin: Energy Balance and Defense of a Stable Body Weight. *Cell Metabolism*.

[52] van Galen K. A., Booij J., Schrantee A. (2021). The Response to Prolonged Fasting in Hypothalamic Serotonin Transporter Availability Is Blunted in Obesity. *Metabolism*.

[53] Zhang X., Ha S., Lau H. C., Yu J. (2023). Excess Body Weight: Novel Insights Into Its Roles in Obesity Comorbidities. *Seminars in Cancer Biology*.

[54] Wajchenberg B. L. (2000). Subcutaneous and Visceral Adipose Tissue: Their Relation to the Metabolic Syndrome. *Endocrine Reviews*.

[55] Kersten S. (2023). The Impact of Fasting on Adipose Tissue Metabolism. *Biochimica et Biophysica Acta, Molecular and Cell Biology of Lipids*.

[56] Zang B. Y., He L. X., Xue L. (2022). Intermittent Fasting: Potential Bridge of Obesity and Diabetes to Health?. *Nutrients*.

[57] Morales-Suarez-Varela M., Collado Sánchez E., Peraita-Costa I., Llopis-Morales A., Soriano J. M. (2021). Intermittent Fasting and the Possible Benefits in Obesity, Diabetes, and Multiple Sclerosis: A Systematic Review of Randomized Clinical Trials. *Nutrients*.

[58] Vasim I., Majeed C. N., DeBoer M. D. (2022). Intermittent Fasting and Metabolic Health. *Nutrients*.

[59] Joaquim L., Faria A., Loureiro H., Matafome P. (2022). Benefits, Mechanisms, and Risks of Intermittent Fasting in Metabolic Syndrome and Type 2 Diabetes. *Journal of Physiology & Biochemistry*.

[60] Asimakidou E., Saipuljumri E. N., Lo C. H., Zeng J. (2025). Role of Metabolic Dysfunction and Inflammation Along the Liver-Brain Axis in Animal Models With Obesity-Induced Neurodegeneration. *Neural Regeneration Research*.

[61] Albrahim T., Alangry R., Alotaibi R., Almandil L., Alburikan S. (2023). Effects of Regular Exercise and Intermittent Fasting on Neurotransmitters, Inflammation, Oxidative Stress, and Brain-Derived Neurotrophic Factor in Cortex of Ovariectomized Rats. *Nutrients*.

[62] Popa A. D., Niță O., Gherasim A. (2023). A Scoping Review of the Relationship between Intermittent Fasting and the Human Gut Microbiota: Current Knowledge and Future Directions. *Nutrients*.

[63] Zhao L., Hutchison A. T., Wittert G. A. (2020). Intermittent Fasting Does Not Uniformly Impact Genes Involved in Circadian Regulation in Women with Obesity. *Obesity*.

[64] Daas M. C., de Roos N. M. (2021). Intermittent Fasting Contributes to Aligned Circadian Rhythms through Interactions with the Gut Microbiome. *Beneficial Microbes*.

[65] Hawley J. A., Sassone-Corsi P., Zierath J. R. (2020). Chrono-nutrition for the Prevention and Treatment of Obesity and Type 2 Diabetes: from Mice to Men. *Diabetologia*.

[66] Patikorn C., Roubal K., Veettil S. K. (2021). Intermittent Fasting and Obesity-Related Health Outcomes: An Umbrella Review of Meta-Analyses of Randomized Clinical Trials. *JAMA Network Open*.

[67] Schroor M. M., Joris P. J., Plat J., Mensink R. P. (2024). Effects of Intermittent Energy Restriction Compared with Those of Continuous Energy Restriction on Body Composition and Cardiometabolic Risk Markers: A Systematic Review and Meta-Analysis of Randomized Controlled Trials in Adults. *Advances in Nutrition*.

