# Supplementary material for: Effect of the 5:2 Diet on Weight Loss and Cardiovascular Disease Risk Factors in Overweight and/or Obesity: A Systematic Review and Meta-Analysis
Source: Int J Endocrinol. 2025 Feb 24;2025:6658512. doi: 10.1155/ije/6658512 (PMC11876533; doi:10.1155/ije/6658512)
Supplement: Supporting Information — Additional supporting information can be found online in the Supporting Information section. [file 6658512.f1.docx]

Effect of the 5:2 Diet on Weight Loss and Cardiovascular Disease Risk Factors in Overweight and/or Obesity: A Systematic Review and Meta-Analysis

Supplementary Material

# Supplementary Data

FIGURE 1 Funnel plots of blood lipids…………………………………………..………4

FIGURE 2 Sensitivity plots of blood lipids………………………..………………..……4

FIGURE 3 Funnel plots of blood pressure and heart rate………………………..……….5

FIGURE 4 Sensitivity plots of blood pressure and heart rate………………………….…5

FIGURE 5 Funnel plots of glucose metabolism……………………………..……..….…6

FIGURE 6 Sensitivity plots of glucose metabolism………………………..…..……..…6

FIGURE 7 Funnel plots of other secondary indicators……………………..…..……..…7

FIGURE 8 Sensitivity plots of other secondary indicators……..…………..…..……..…8

FIGURE 9 Forest map of body weight analysed in subgroups by geographic location…9

FIGURE 10 Forest map of body weight analysed in subgroups by treatment duration…10

FIGURE 11 Forest map of body weight analysed in subgroups by sample size...………11

FIGURE 12 Forest map of body weight analysed in subgroups by age…………………12

FIGURE 13 Forest map of body weight analysed in subgroups by gender…………...…13

FIGURE 14 Forest map of body mass index analysed in subgroups by geographic location...14

FIGURE 15 Forest map of body mass index analysed in subgroups by treatment duration….15

FIGURE 16 Forest map of body mass index analysed in subgroups by sample size…………15

FIGURE 17 Forest map of body mass index analysed in subgroups by age…………….……16

FIGURE 18 Forest map of body mass index analysed in subgroups by gender…………...…16

FIGURE 19 Forest map of total cholesterol analysed in subgroups by geographic location…17

FIGURE 20 Forest map of total cholesterol analysed in subgroups by treatment duration…..17

FIGURE 21 Forest map of total cholesterol analysed in subgroups by sample size………18

FIGURE 22 Forest map of total cholesterol analysed in subgroups by age…………….…18

FIGURE 23 Forest map of total cholesterol analysed in subgroups by gender……………19

FIGURE 24 Forest map of triglycerides analysed in subgroups by geographic location…19

FIGURE 25 Forest map of triglycerides analysed in subgroups by treatment duration…..20

FIGURE 26 Forest map of triglycerides analysed in subgroups by sample size…………20

FIGURE 27 Forest map of triglycerides analysed in subgroups by age……………….…21

FIGURE 28 Forest map of triglycerides analysed in subgroups by gender………………21

FIGURE 29 Forest map of low-density lipoprotein analysed in subgroups by geographic location………………………………………………….. …………………………..…...22

FIGURE 30 Forest map of low-density lipoprotein analysed in subgroups by treatment duration………………………………………………….. …………………………..…..23

FIGURE 31 Forest map of low-density lipoprotein analysed in subgroups by sample size……………………………………………………….. ………………………………23

FIGURE 32 Forest map of low-density lipoprotein analysed in subgroups by age………23

FIGURE 33 Forest map of low-density lipoprotein analysed in subgroups by gender……24

FIGURE 34 Forest map of high-density lipoprotein analysed in subgroups by geographic location………………………………………………….. …………………………..……25

FIGURE 35 Forest map of high-density lipoprotein analysed in subgroups by treatment duration………………………………………………….. …………………………..……26

FIGURE 36 Forest map of high-density lipoprotein analysed in subgroups by sample size……………………………………………………….. …………………………..……26

FIGURE 37 Forest map of high-density lipoprotein analysed in subgroups by age……….27

FIGURE 38 Forest map of high-density lipoprotein analysed in subgroups by gender……27

FIGURE 39 Forest map of diastolic blood pressure analysed in subgroups by geographic location………………………………………………….. …………………………..……28

FIGURE 40 Forest map of diastolic blood pressure analysed in subgroups by treatment duration……………………………………………….. …………………………..………28

FIGURE 41 Forest map of diastolic blood pressure analysed in subgroups by sample size……………………………………………………….. ……………………………..28

FIGURE 42 Forest map of diastolic blood pressure analysed in subgroups by age……..29

FIGURE 43 Forest map of diastolic blood pressure analysed in subgroups by gender….29

FIGURE 44 Forest map of insulin analysed in subgroups by geographic location…...…30

FIGURE 45 Forest map of insulin analysed in subgroups by treatment duration……….30

FIGURE 46 Forest map of insulin analysed in subgroups by sample size…………….…31

FIGURE 47 Forest map of insulin analysed in subgroups by age……………………..…31

FIGURE 48 Forest map of insulin analysed in subgroups by gender………………….…32

FIGURE 49 Forest map of fat mass analysed in subgroups by geographic location……..32

FIGURE 50 Forest map of fat mass analysed in subgroups by treatment duration………33

FIGURE 51 Forest map of fat mass analysed in subgroups by sample size………..……33

FIGURE 52 Forest map of fat mass analysed in subgroups by age………………………34

FIGURE 53 Forest map of fat mass analysed in subgroups by gender………………..…34

TABLE 1 PRISMA checklist……………………………………………………….……35

TABLE 2 Search strategy in PubMed……………………………...……………..….…..39

TABLE 3 GRADE quality of evidence evaluation form…………...……………..…......41

# Supplementary Figures and TABLEs

## Supplementary Figure

##
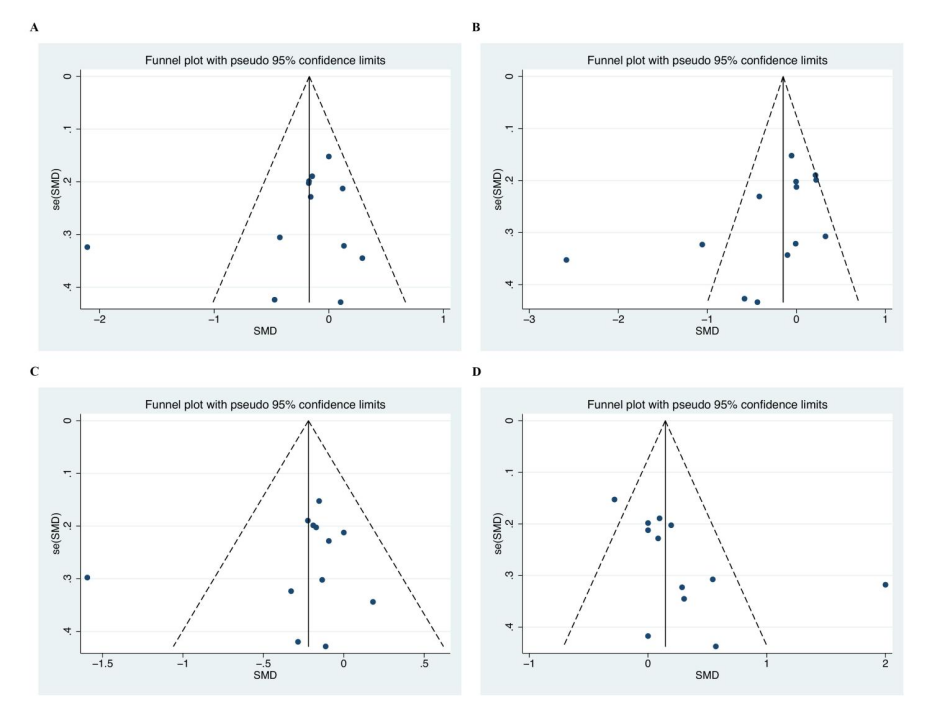


**Supplementary Figure 1**. Funnel plots of blood lipids. (A) Total cholesterol (TC); (B) Triglyceride (TG); (C) Low density lipoprotein (LDL); (D) High density lipoprotein (HDL).


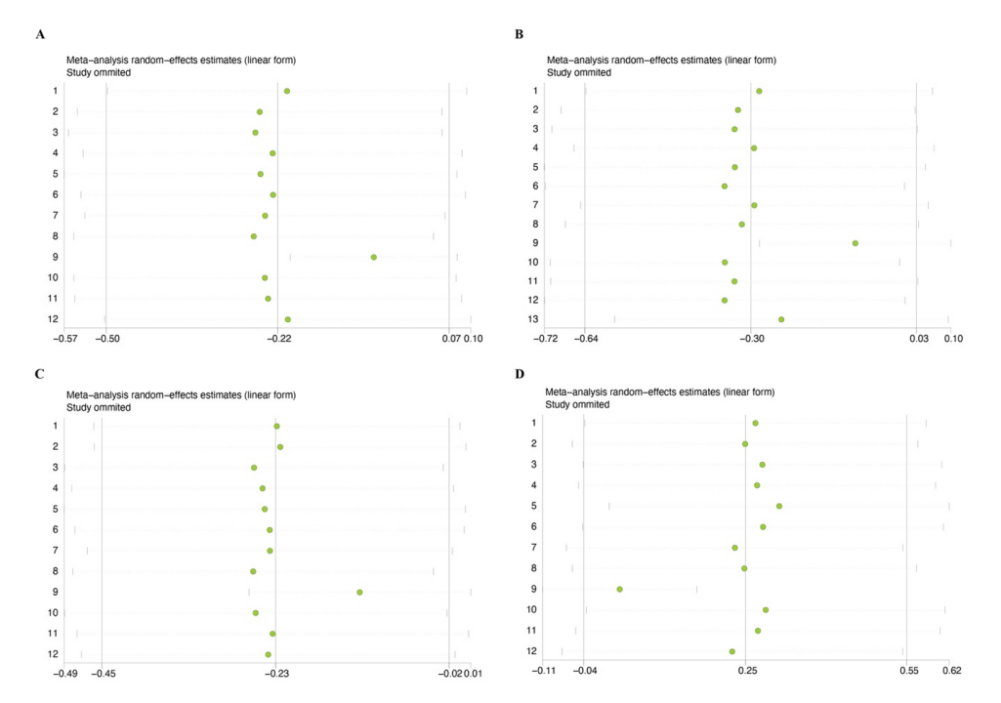


**Supplementary Figure 2.** Sensitivity plots of blood lipids. (A) Total cholesterol (TC); (B) Triglyceride (TG); (C) Low density lipoprotein (LDL); (D) High density lipoprotein (HDL).


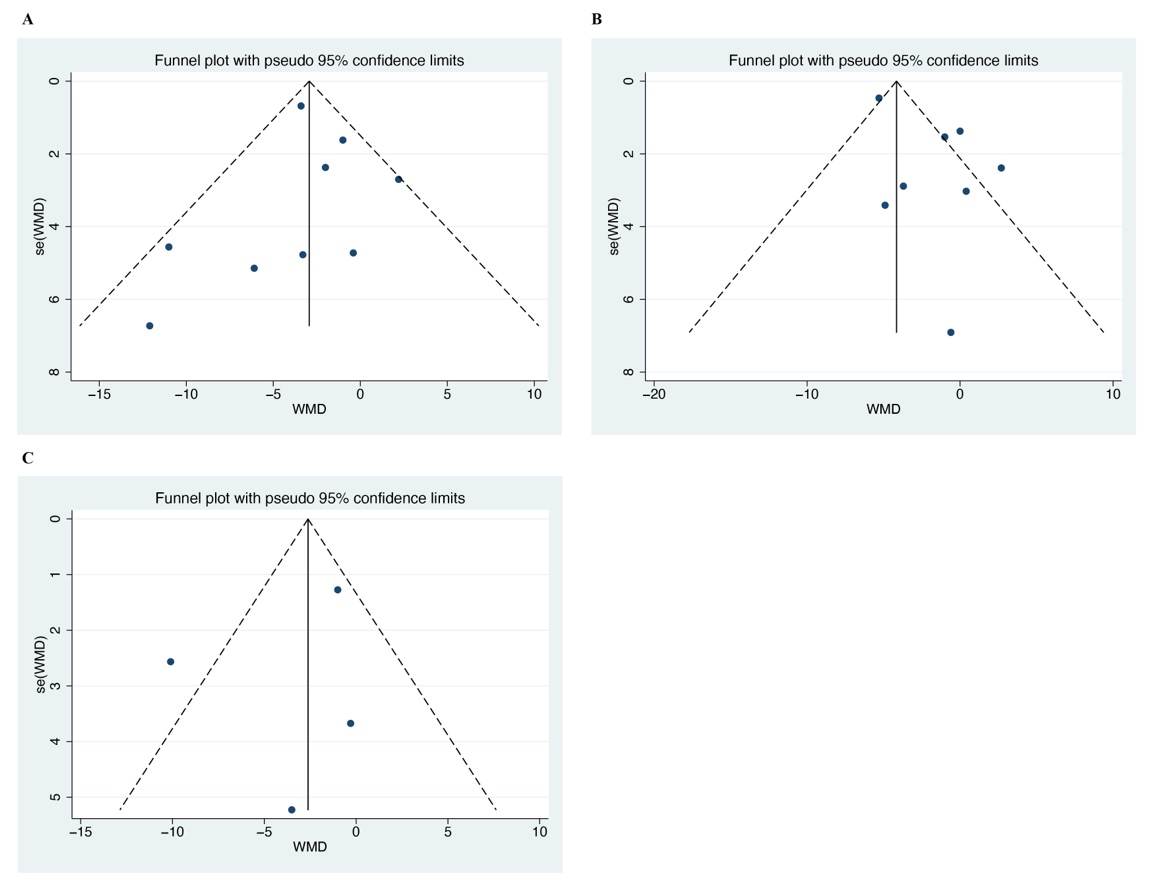


**Supplementary Figure 3.** Funnel plots of blood pressure. (A) Systolic blood pressure (SBP); (B) Diastolic blood pressure (DBP); (C) Heart rate.


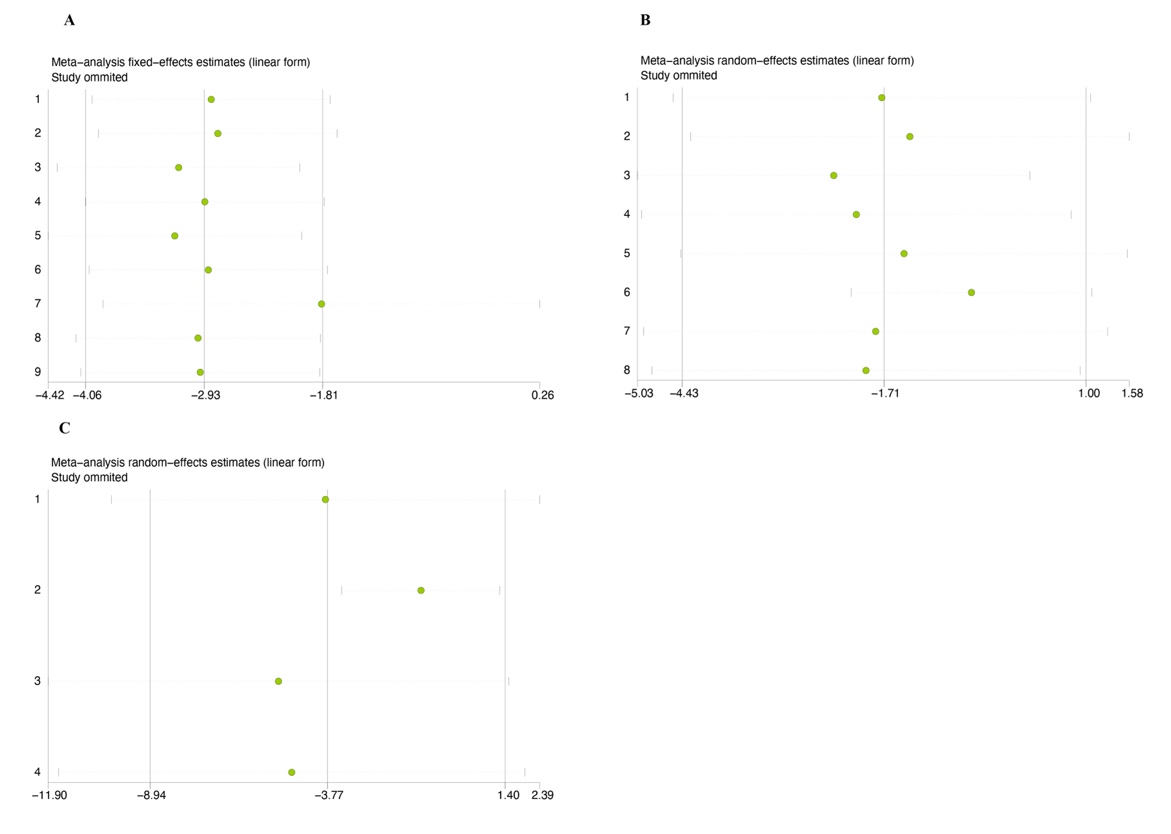


**Supplementary Figure 4.** Sensitivity plots of blood pressure. (A) Systolic blood pressure (SBP); (B) Diastolic blood pressure (DBP); (C) Heart rate.


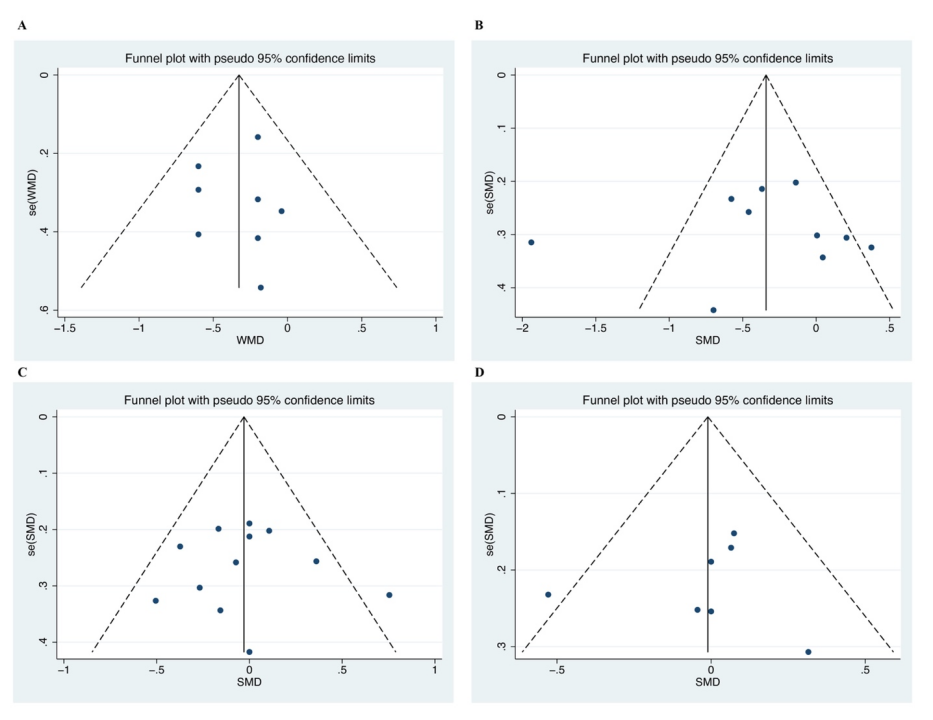


**Supplementary Figure 5.** Funnel plots of glucose metabolism. (A) HOMA-IR; (B) Insulin; (C) FBG; (D) HbA1c.


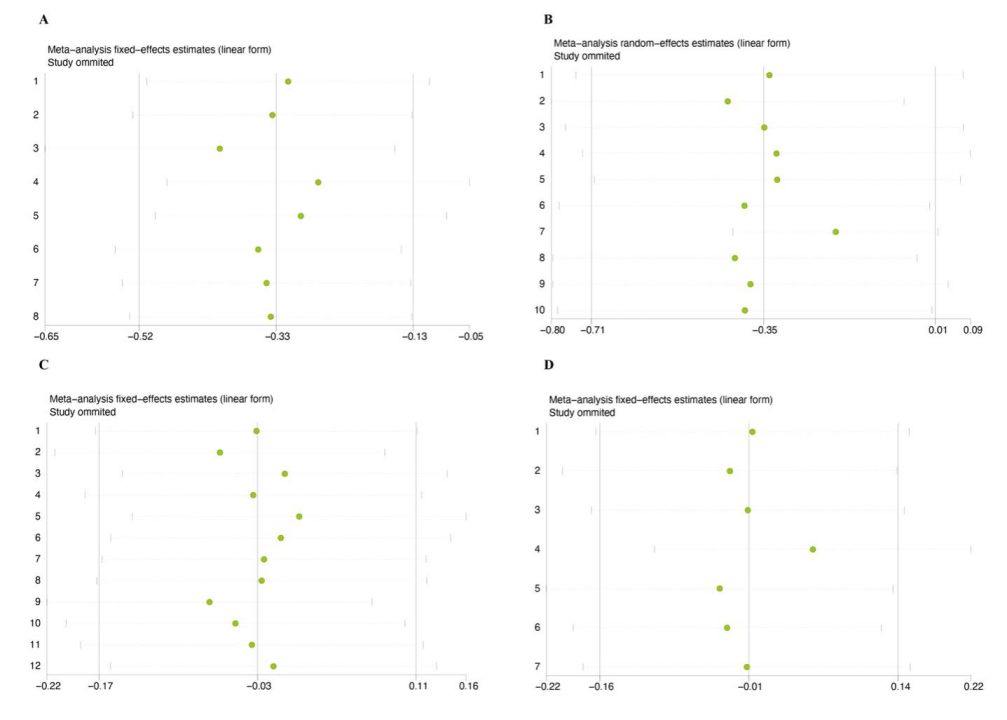


**Supplementary Figure 6.** Sensitivity plots of glucose metabolism. (A) HOMA-IR; (B) Insulin; (C) FBG; (D) HbA1c.


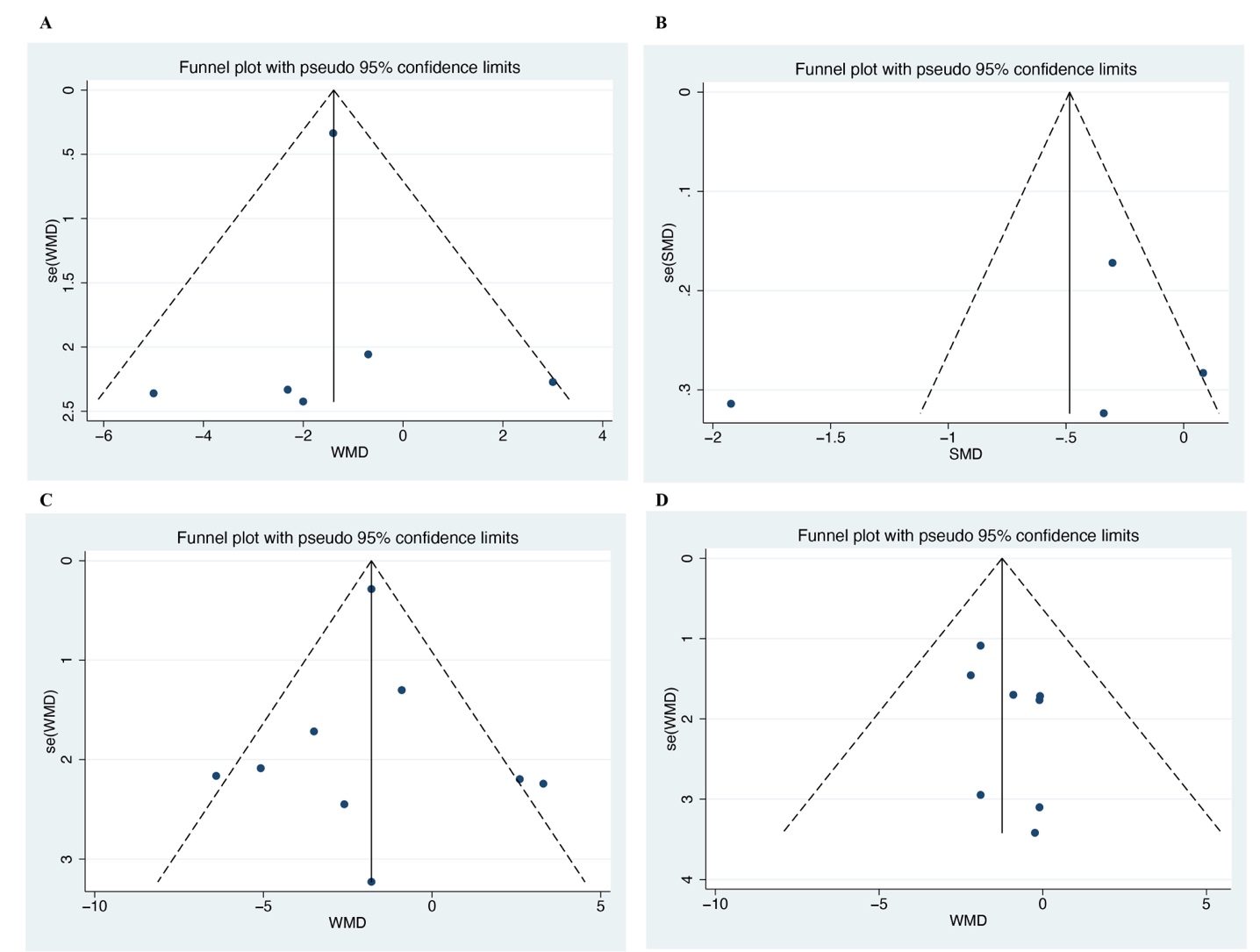


**Supplementary Figure 7.** Funnel plots of other secondary indicators. (A) Hip circumference; (B) Visceral fat; (C) Fat mass; (D) Fat-free mass.


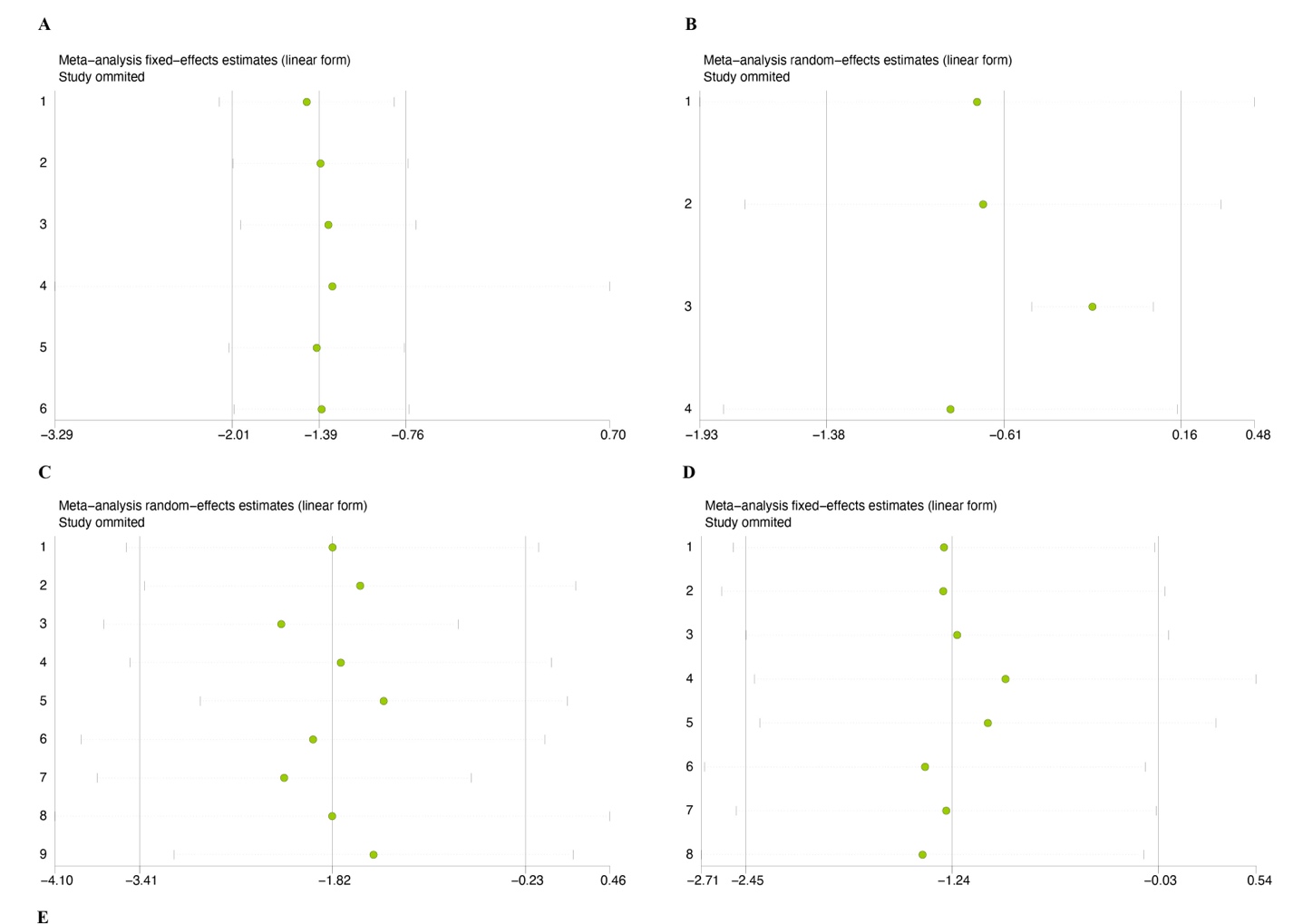


**Supplementary Figure 8.** Sensitivity plots of other secondary indicators. (A) Hip circumference; (B) Visceral fat; (C) Fat mass; (D) Fat-free mass.


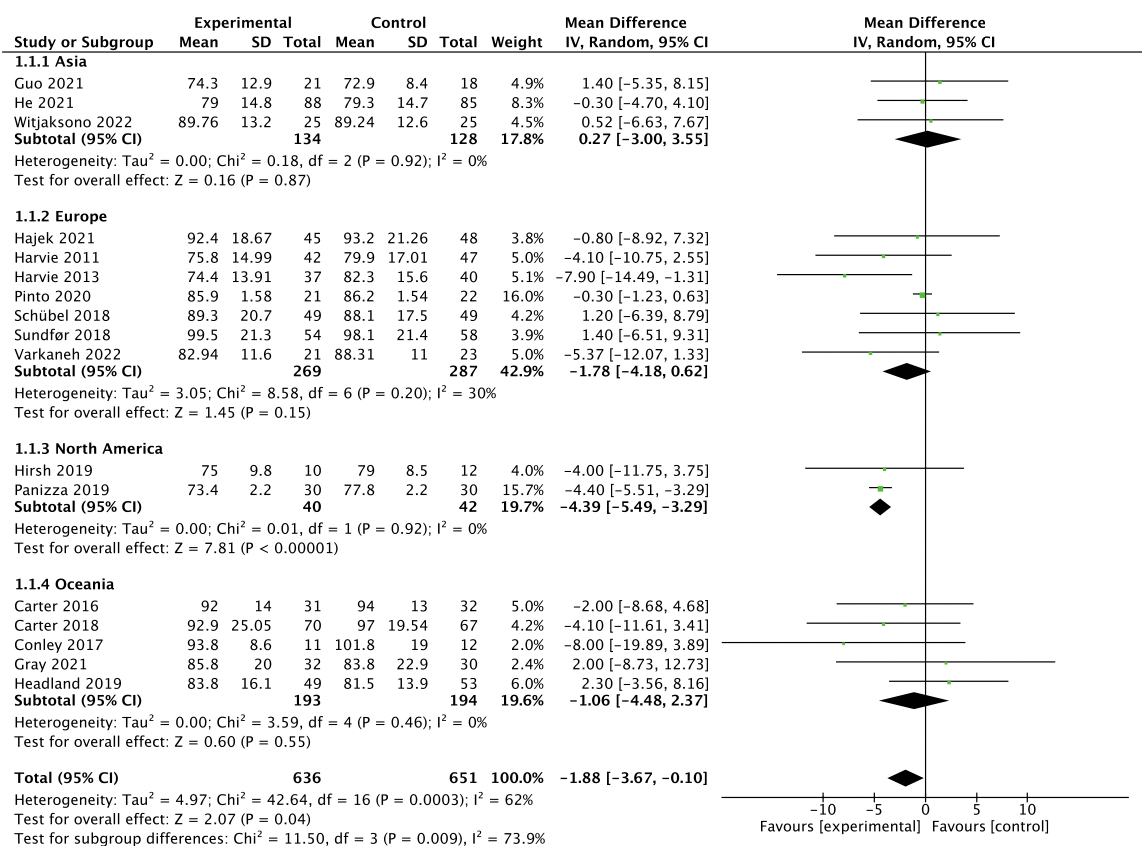


**Supplementary Figure 9.** Forest map of body weight analysed in subgroups by geographic location.


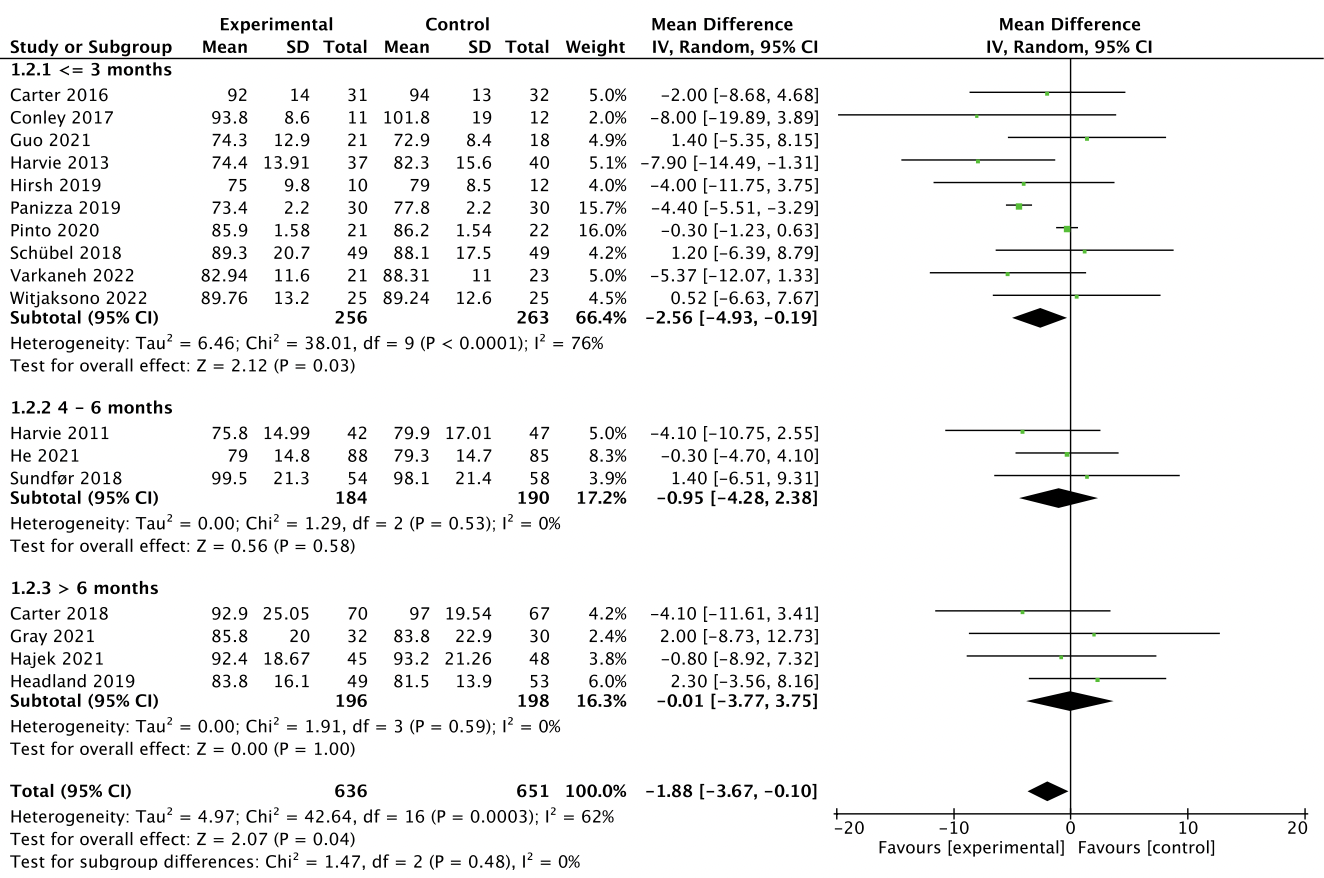


**Supplementary Figure 10.** Forest map of body weight analysed in subgroups by treatment duration.


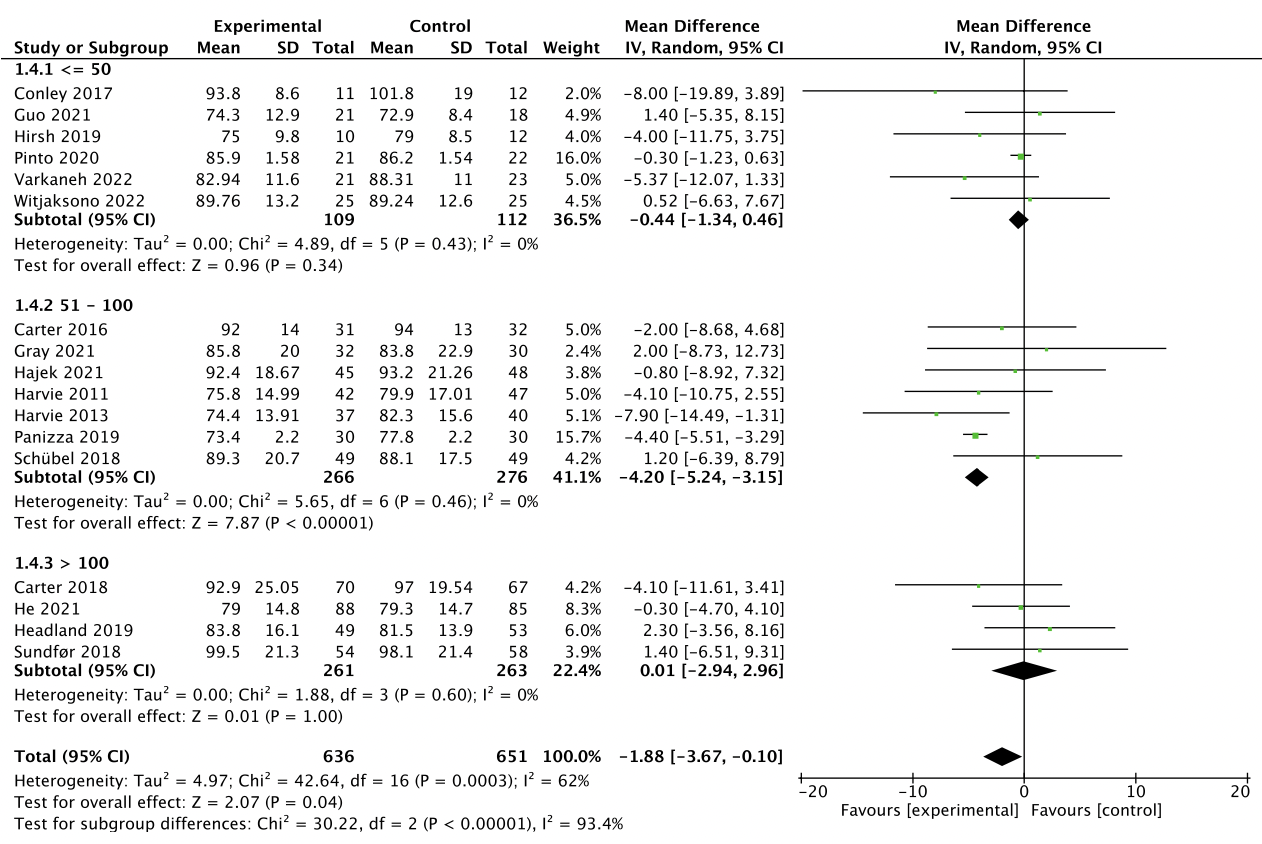


**Supplementary Figure 11.** Forest map of body weight analysed in subgroups by sample size.


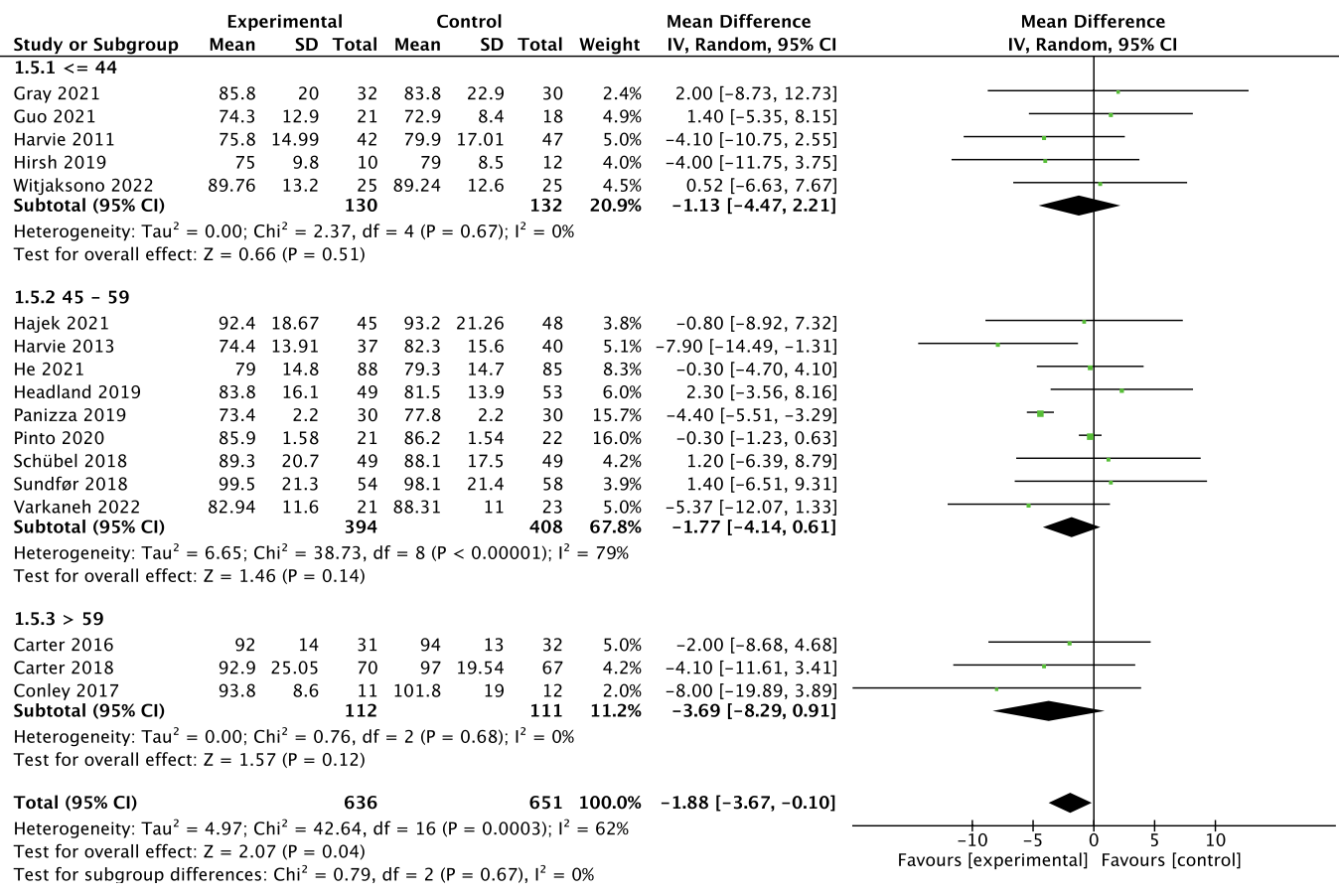


**Supplementary Figure 12.** Forest map of body weight analysed in subgroups by age.


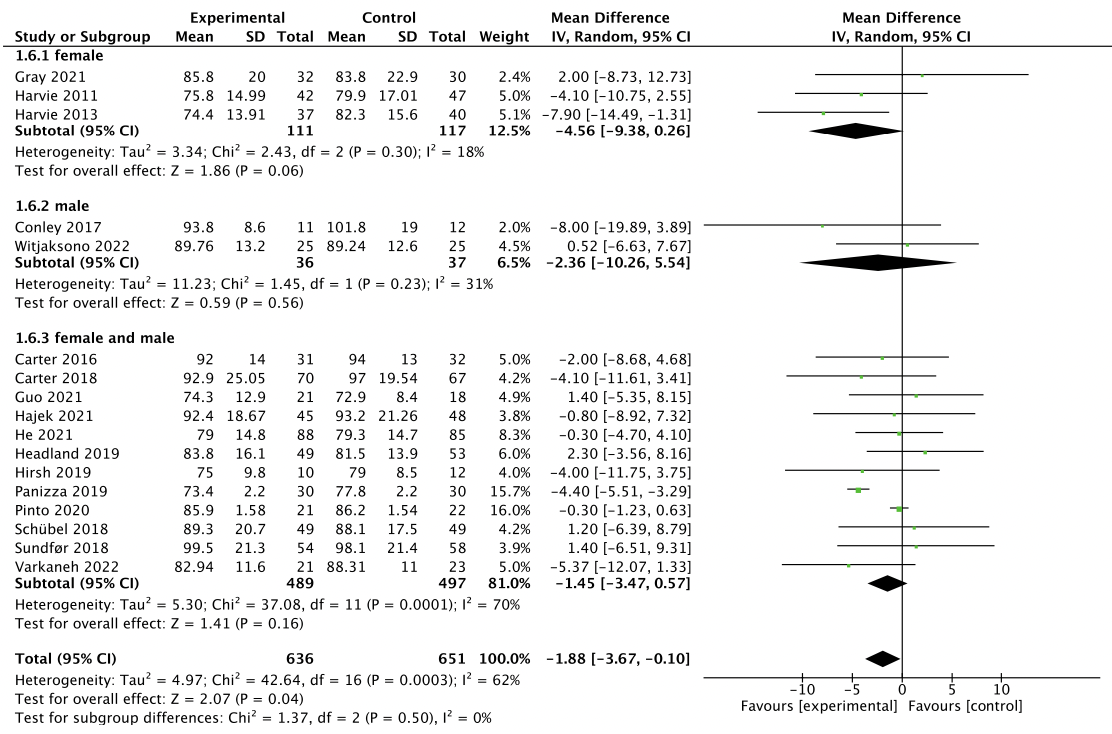


**Supplementary Figure 13.** Forest map of body weight analysed in subgroups by gender.


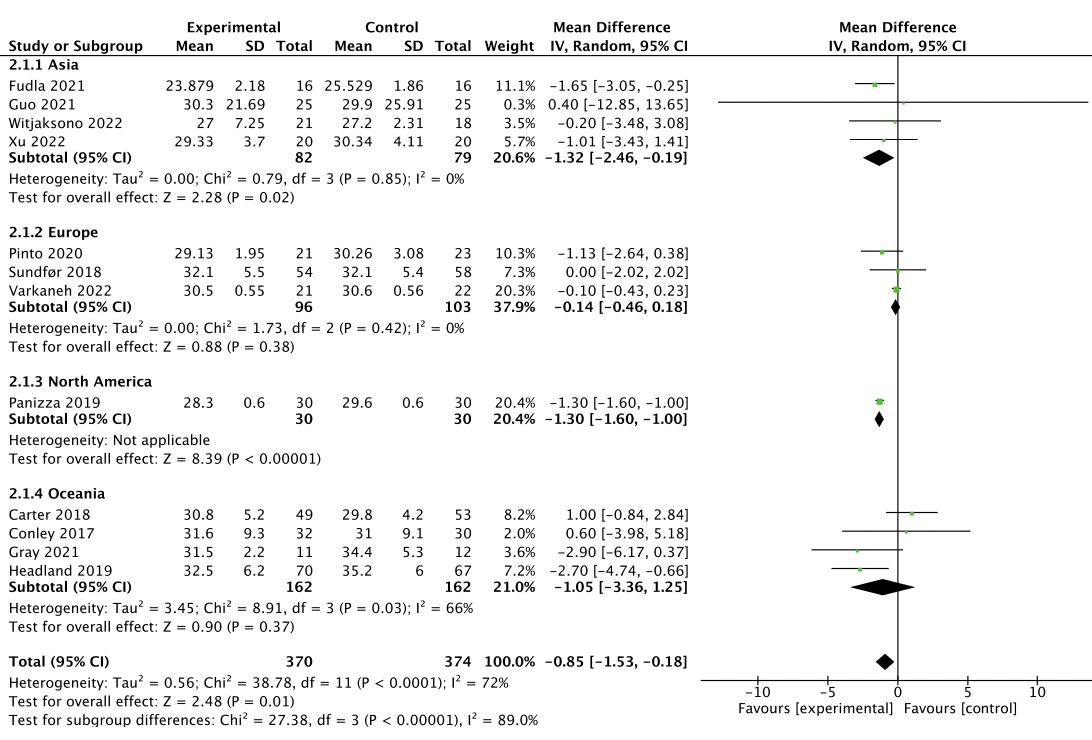


**Supplementary Figure 14.** Forest map of body mass index analysed in subgroups by geographic location.


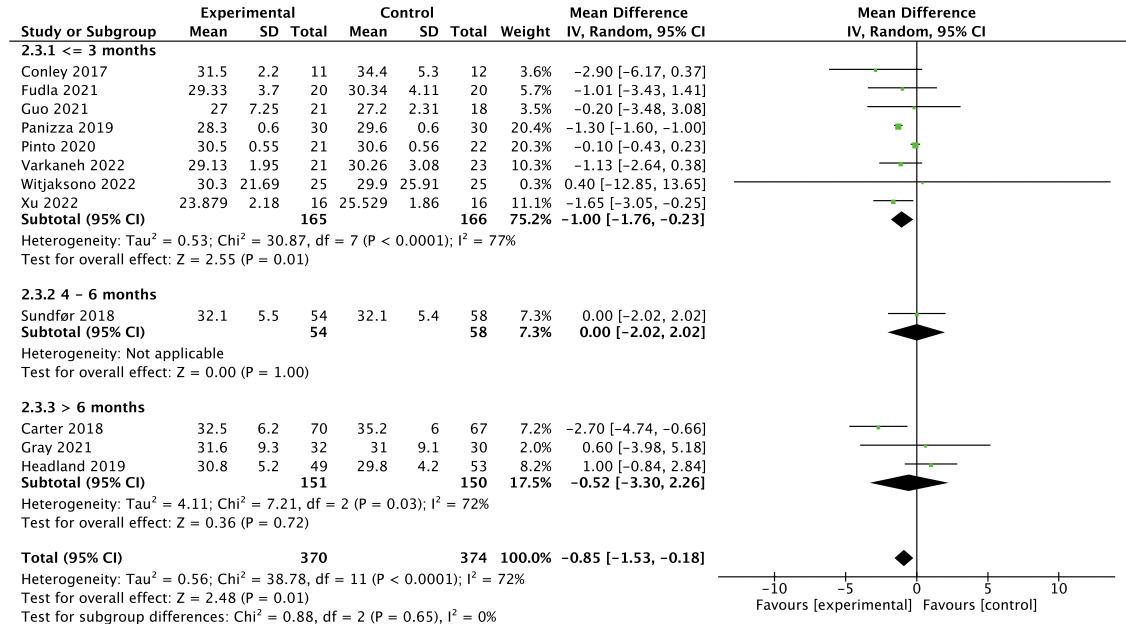


**Supplementary Figure 15.** Forest map of body mass index analysed in subgroups by treatment duration.


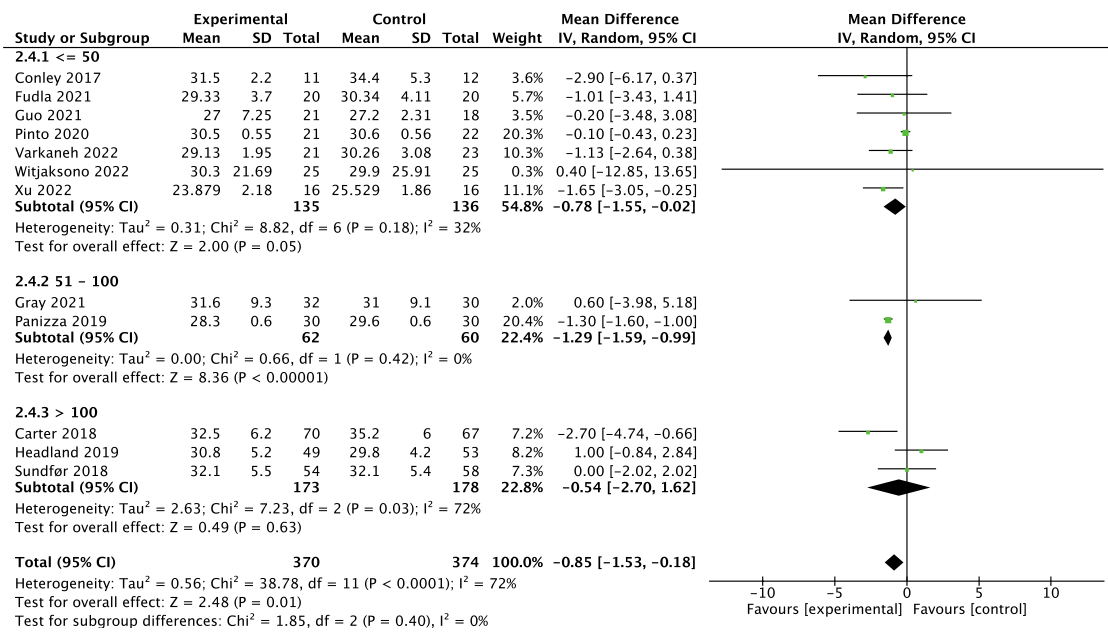


**Supplementary Figure 16.** Forest map of body mass index analysed in subgroups by sample size.


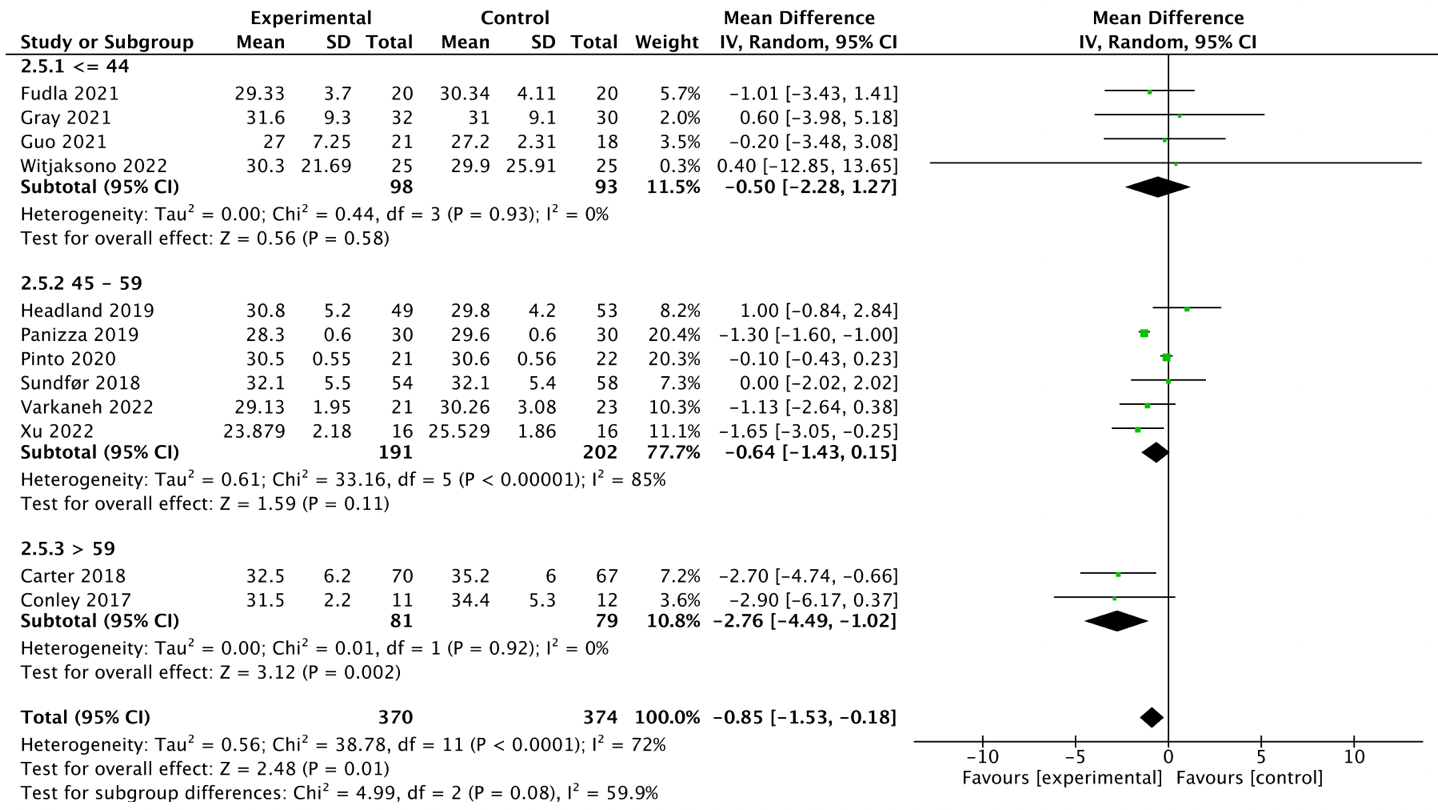


**Supplementary Figure 17.** Forest map of body mass index analysed in subgroups by age.


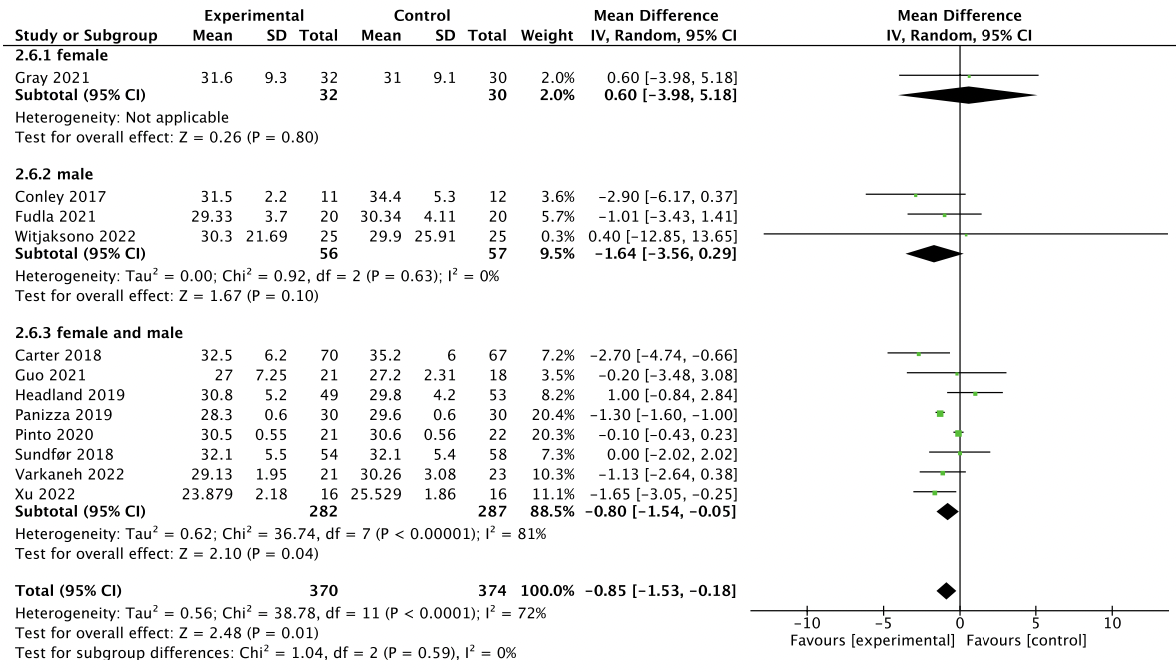


**Supplementary Figure 18.** Forest map of body mass index analysed in subgroups by gender.


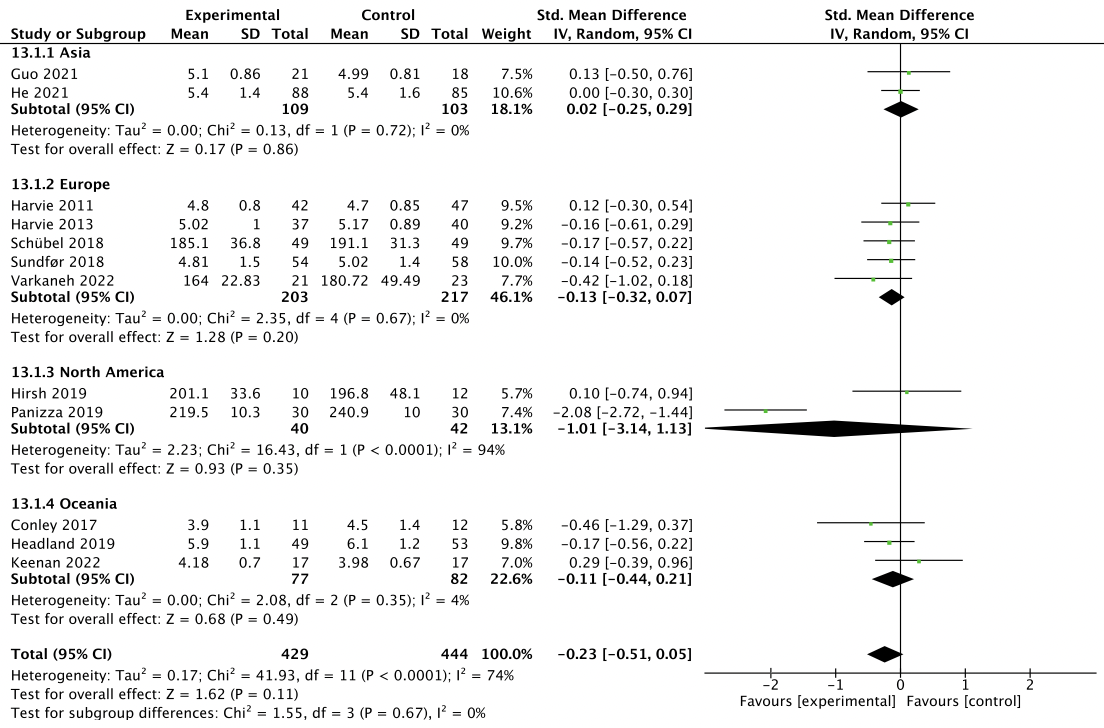


**Supplementary Figure 19.** Forest map of total cholesterol analysed in subgroups by geographic location.


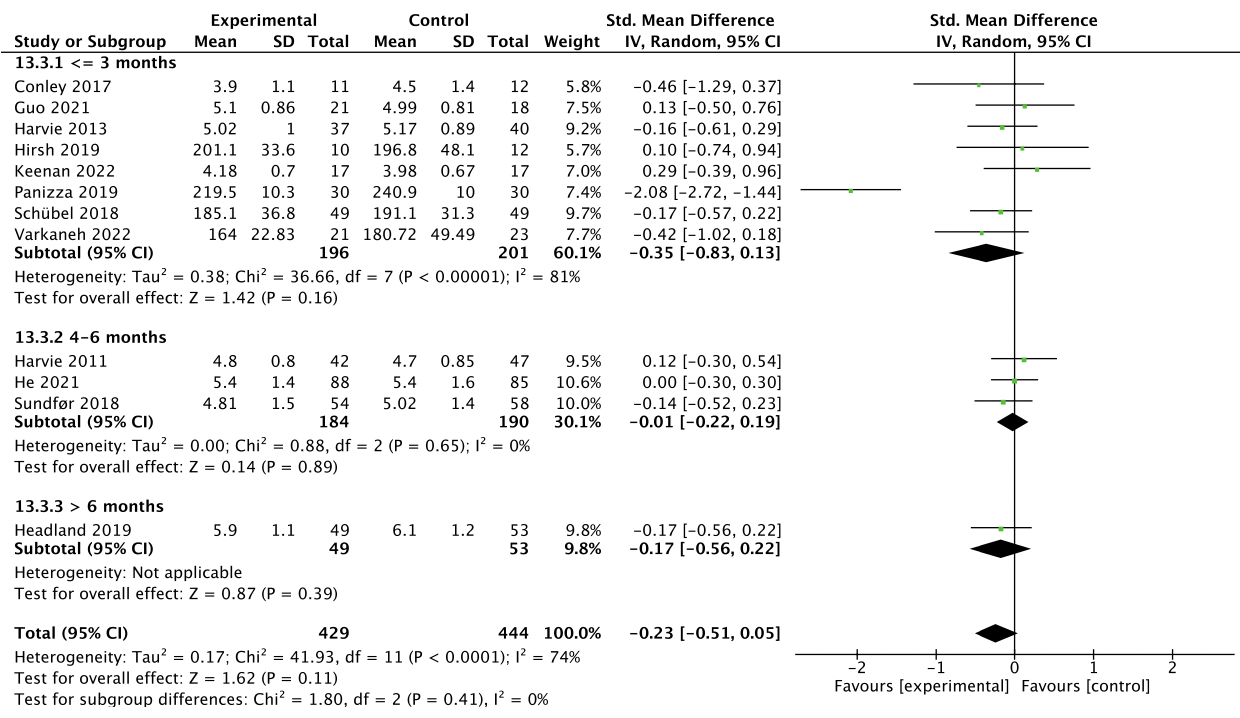


**Supplementary Figure 20.** Forest map of total cholesterol analysed in subgroups by treatment duration.


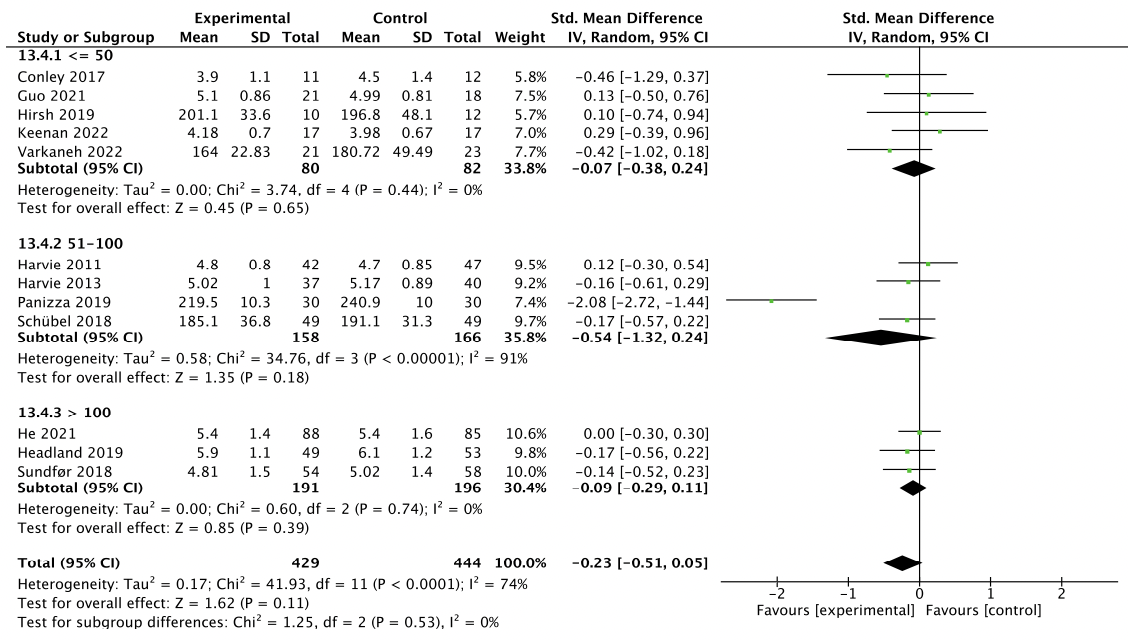


**Supplementary Figure 21.** Forest map of total cholesterol analysed in subgroups by sample size.


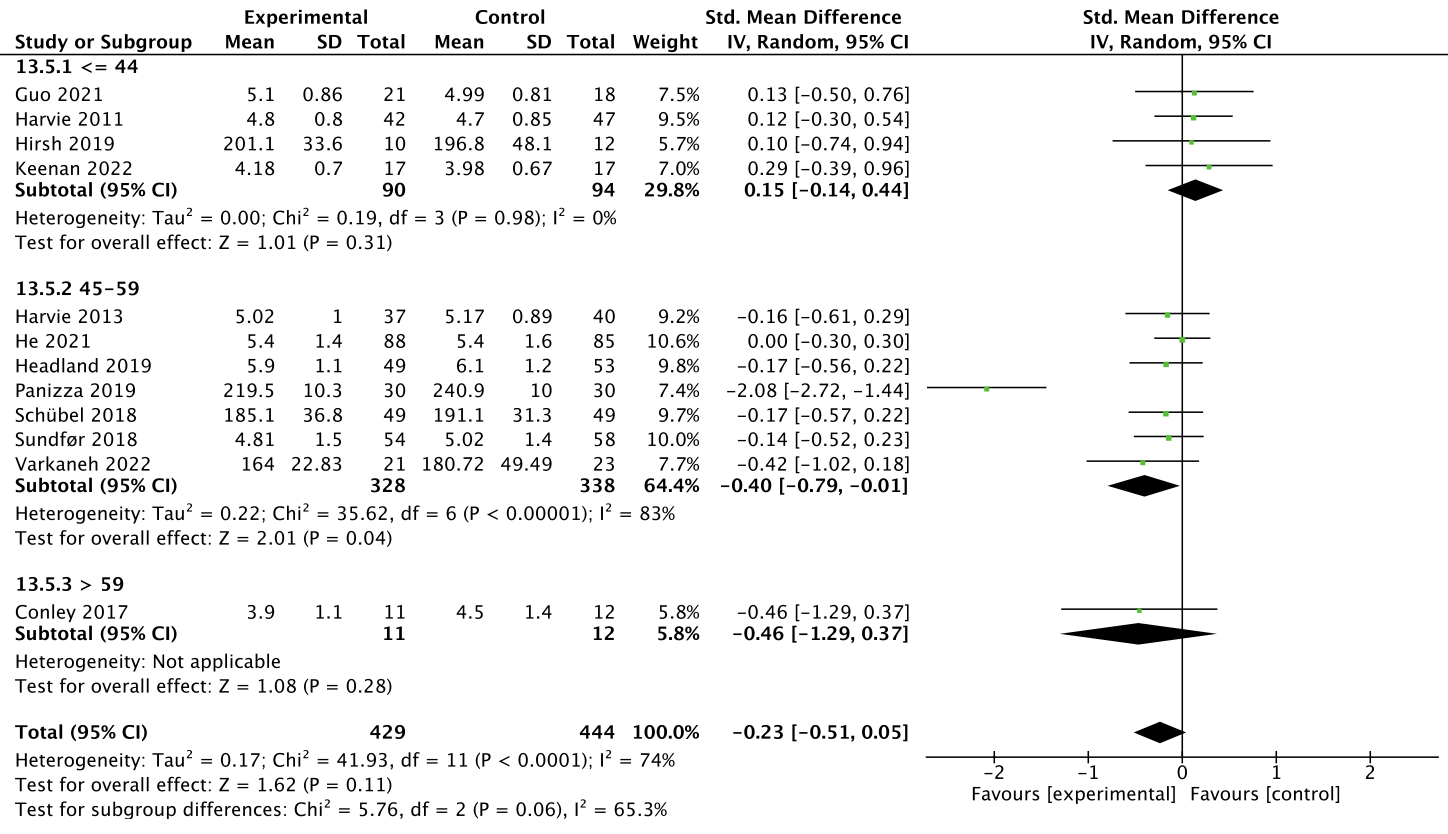


**Supplementary Figure 22.** Forest map of total cholesterol analysed in subgroups by age.


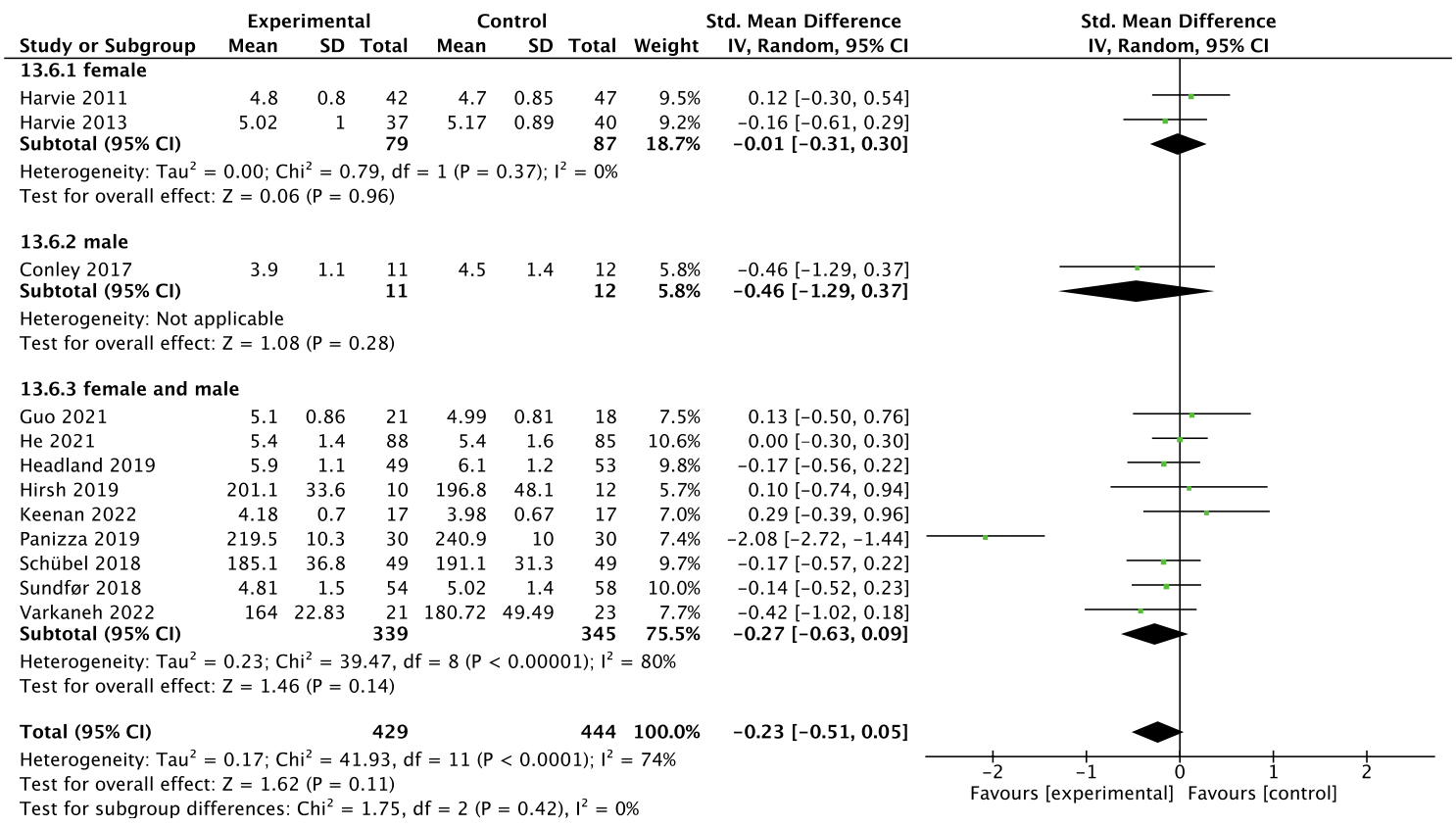


**Supplementary Figure 23.** Forest map of total cholesterol analysed in subgroups by gender.


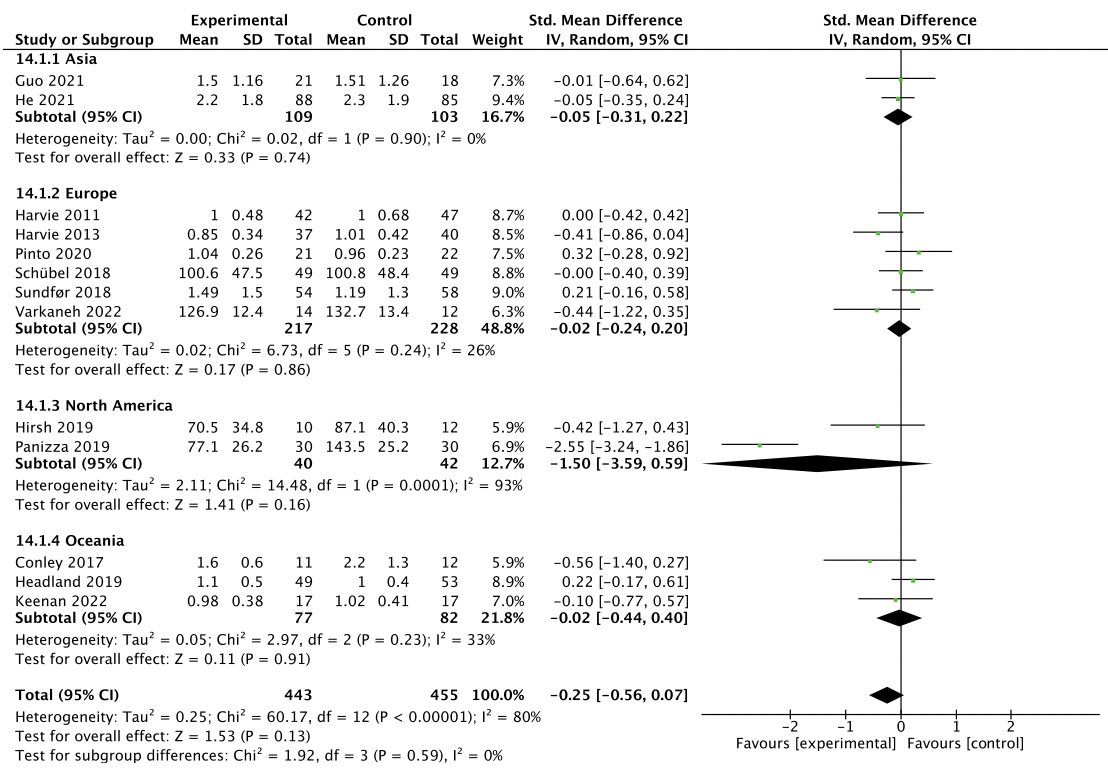


**Supplementary Figure 24.** Forest map of triglycerides analysed in subgroups by geographic location.


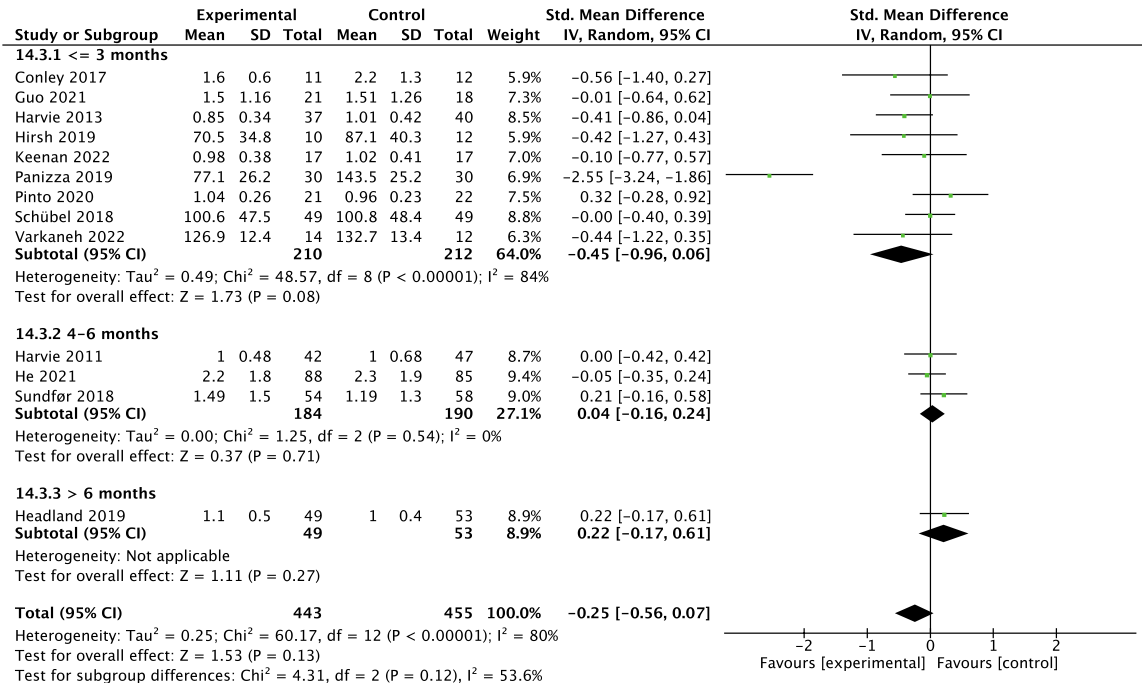


**Supplementary Figure 25.** Forest map of triglycerides analysed in subgroups by treatment duration.


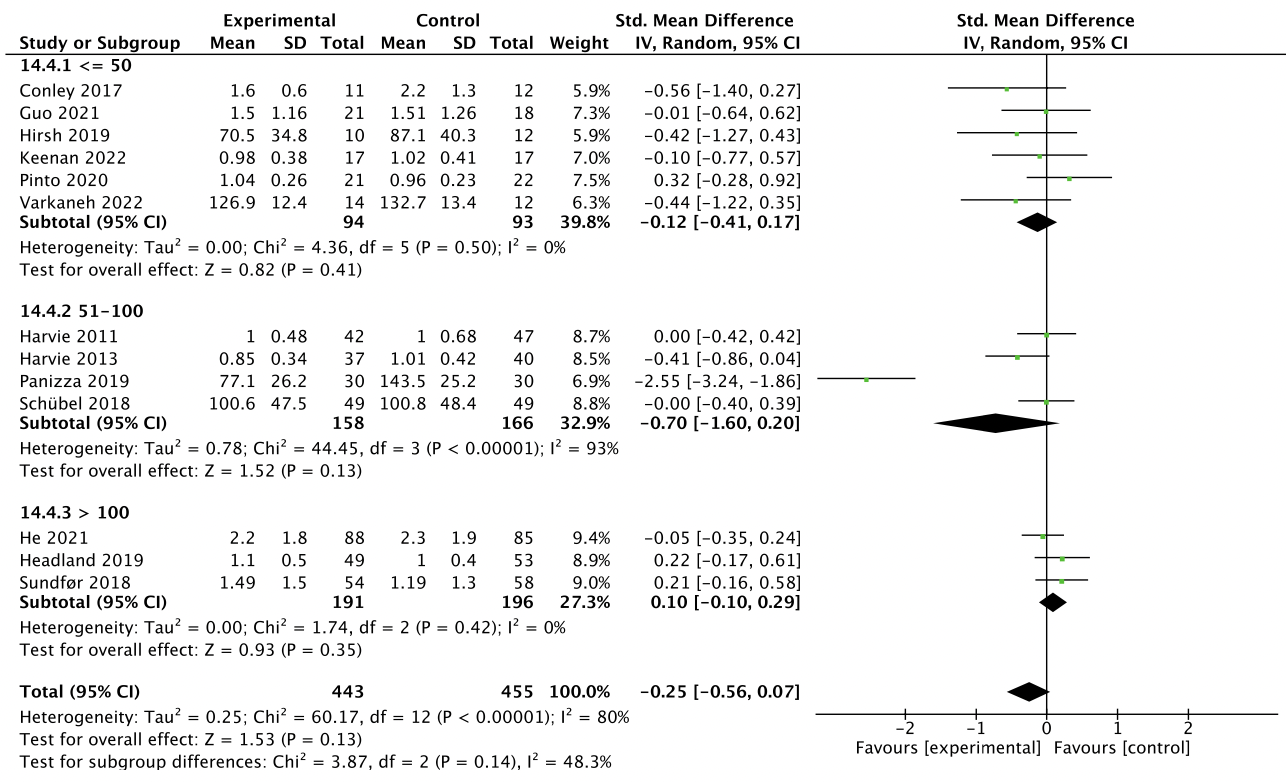


**Supplementary Figure 26.** Forest map of triglycerides analysed in subgroups by sample size.


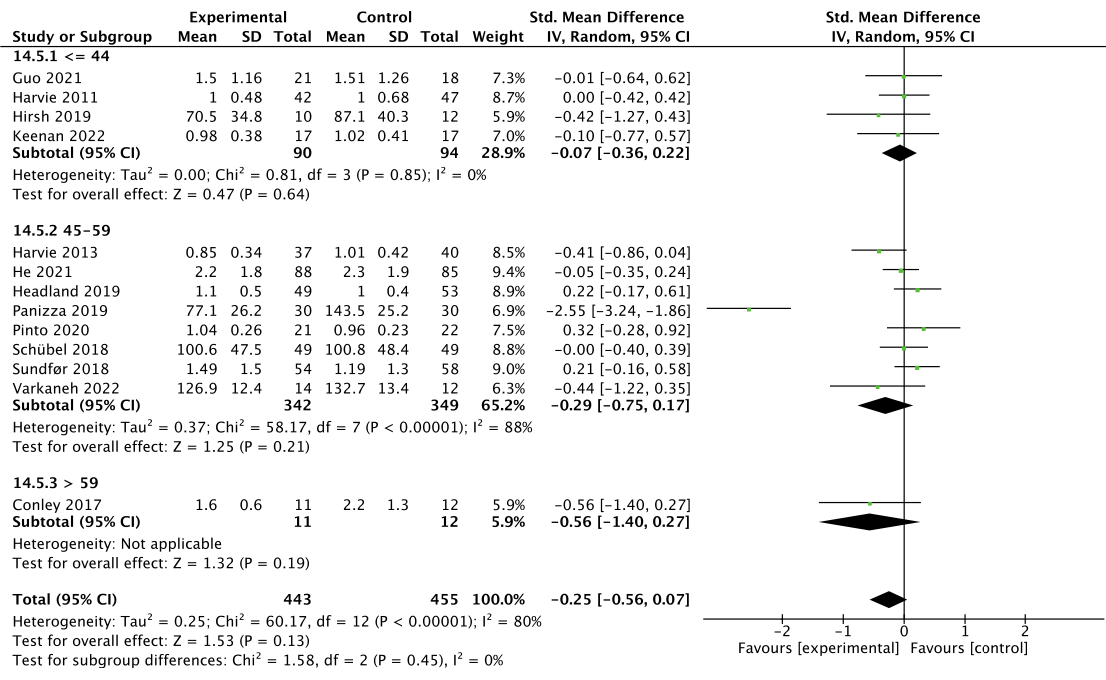


**Supplementary Figure 27.** Forest map of triglycerides analysed in subgroups by age.


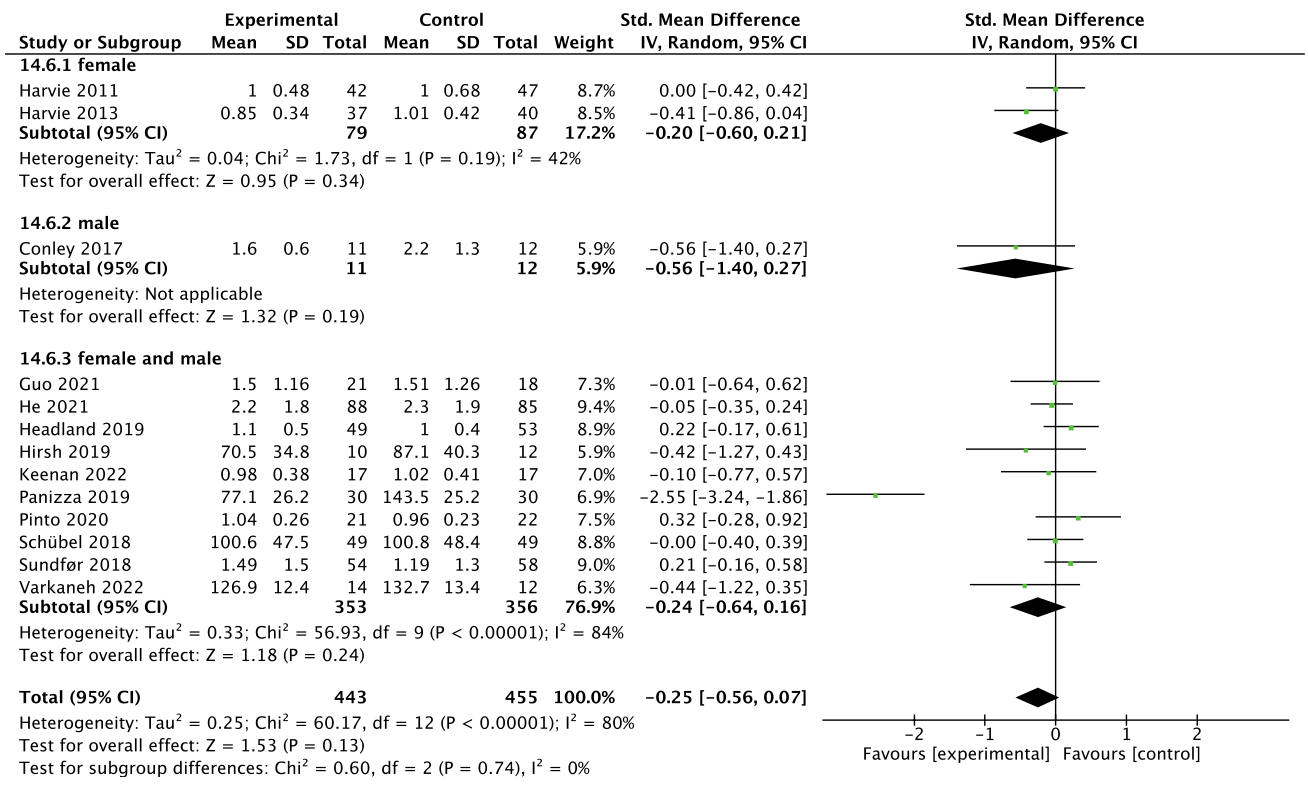


**Supplementary Figure 28.** Forest map of triglycerides analysed in subgroups by gender.

**
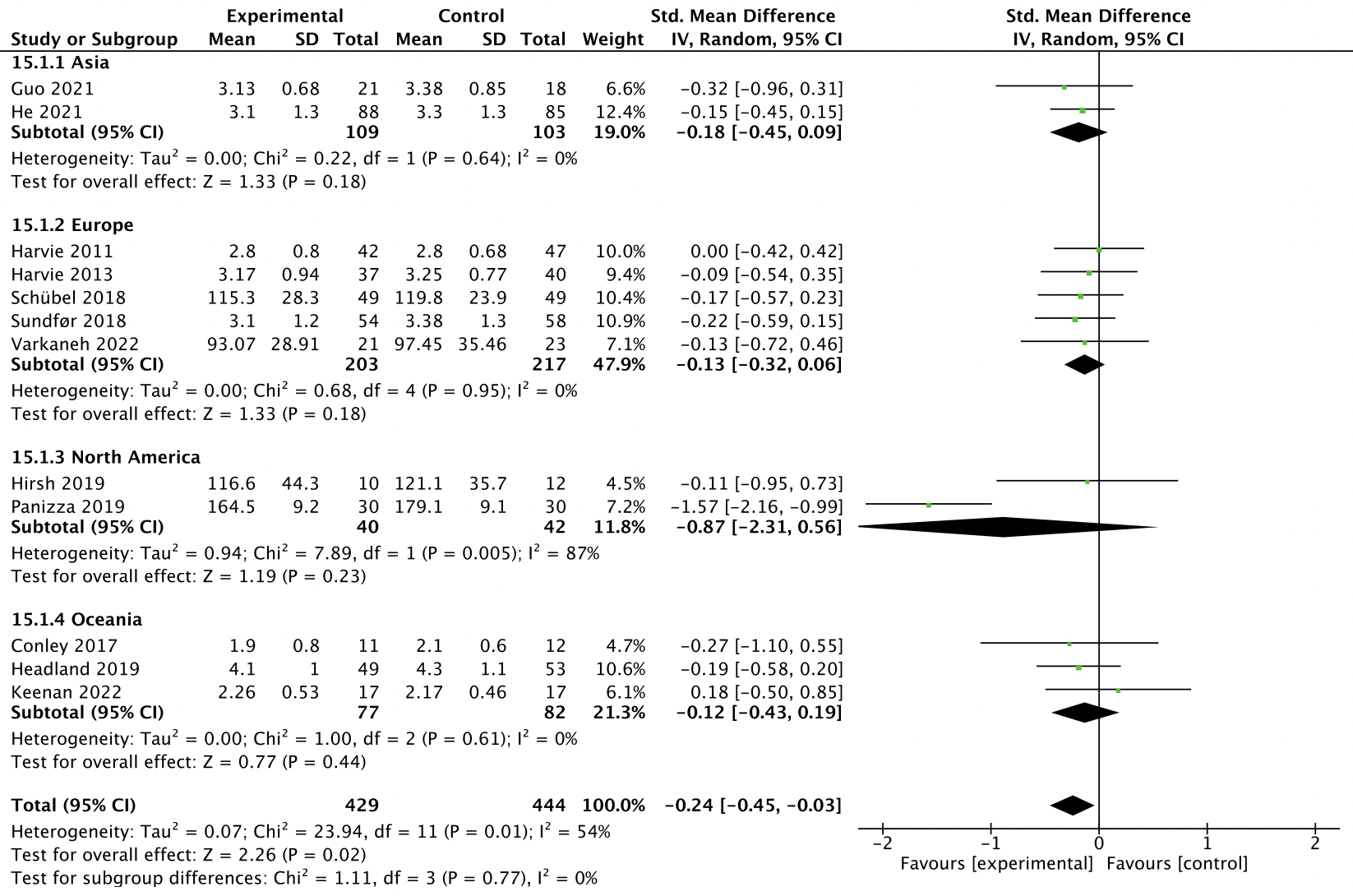
**

**Supplementary Figure 29.** Forest map of low-density lipoprotein analysed in subgroups by geographic location.


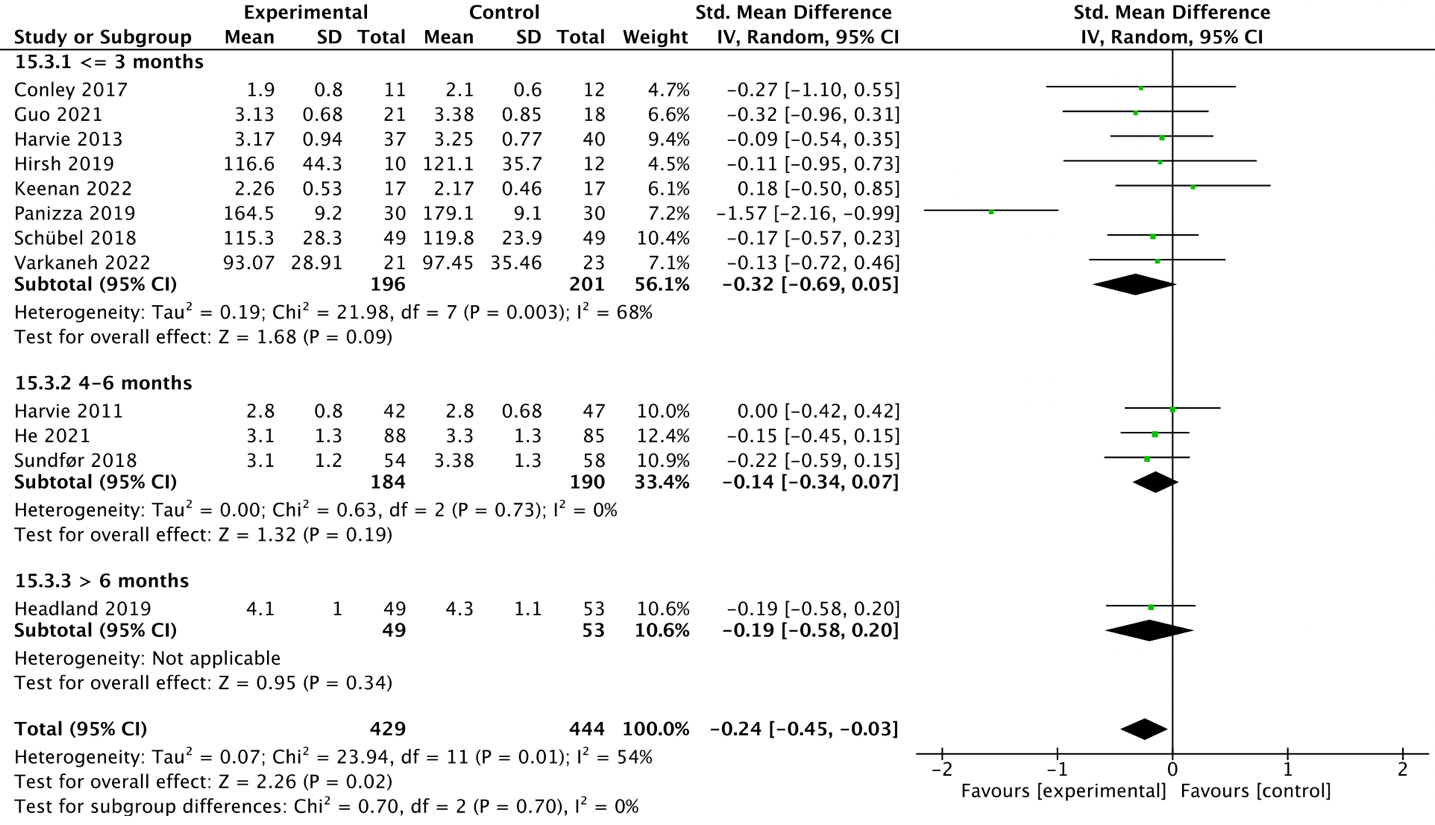


**Supplementary Figure 30.** Forest map of low-density lipoprotein analysed in subgroups by treatment duration.


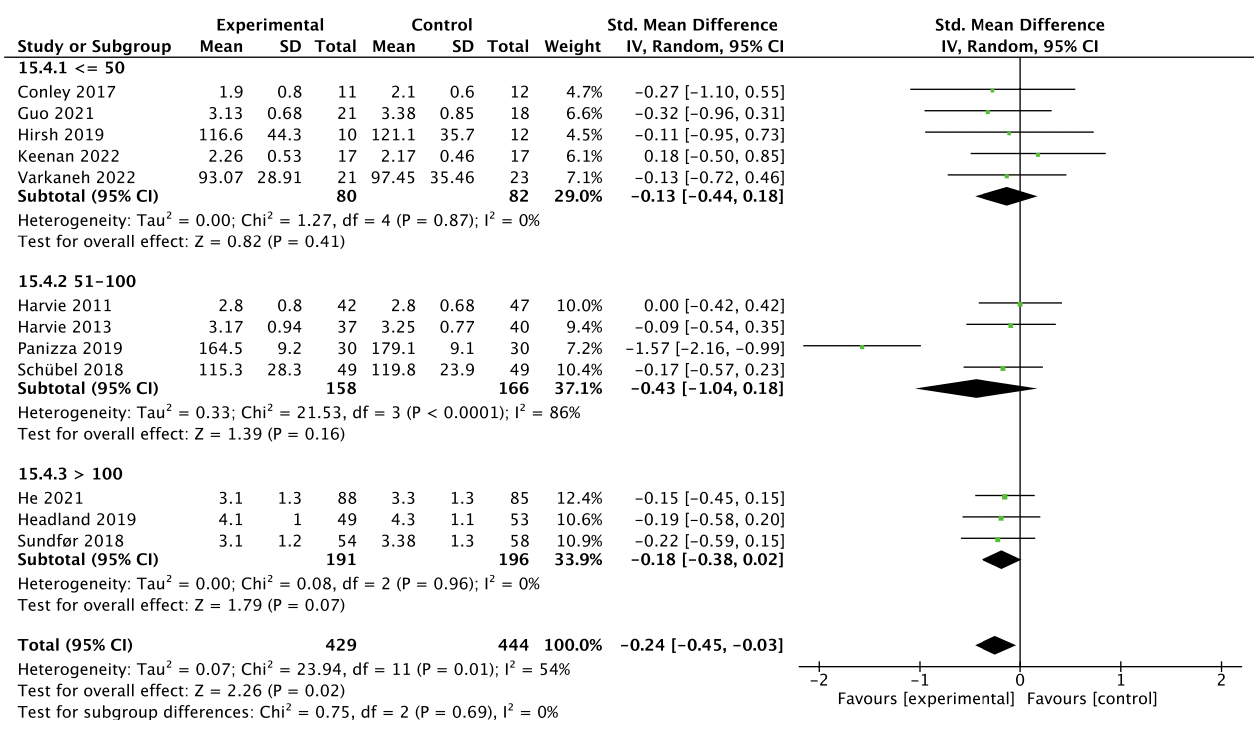


**Supplementary Figure 31.** Forest map of low-density lipoprotein analysed in subgroups by treatment duration.


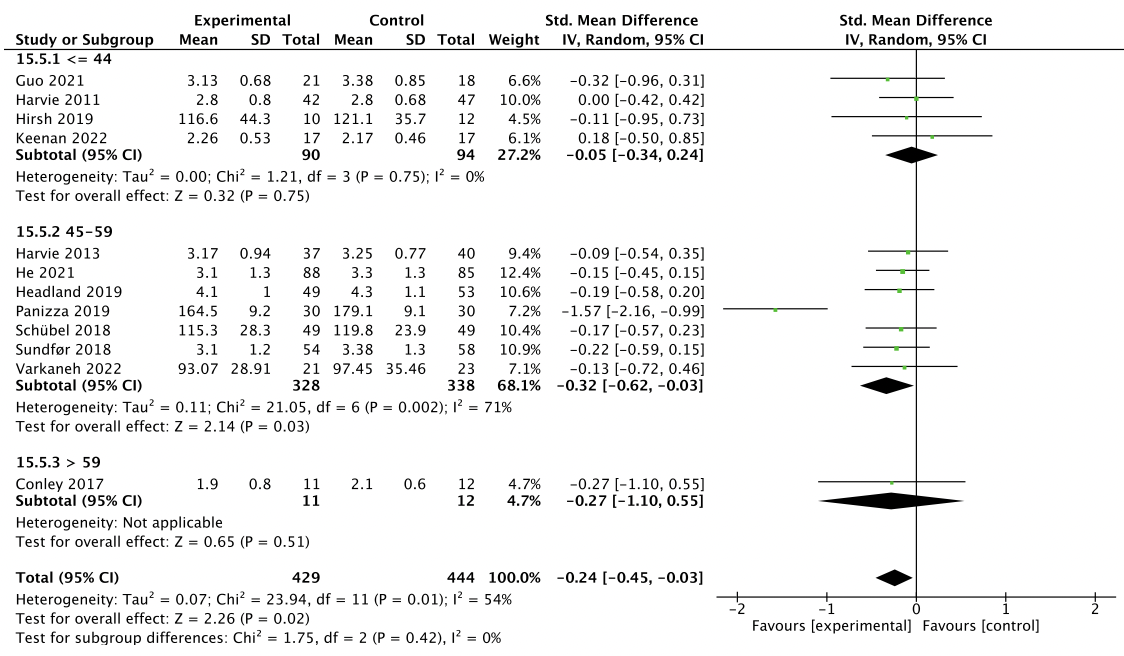


**Supplementary Figure 32.** Forest map of low-density lipoprotein analysed in subgroups by age.


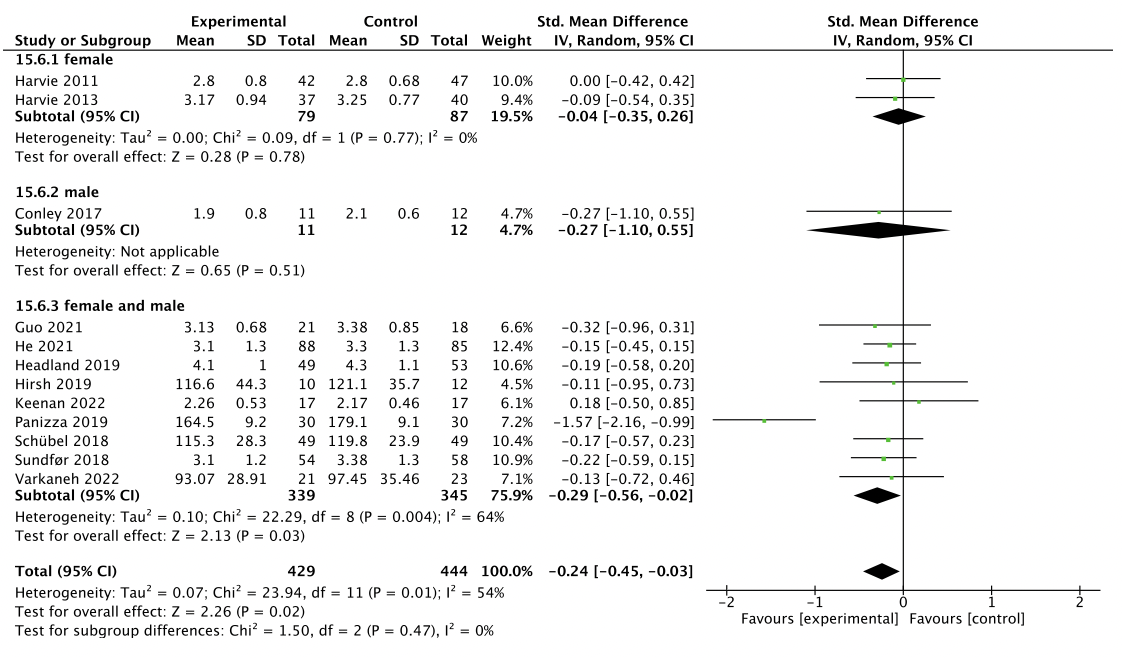


**Supplementary Figure 33.** Forest map of low-density lipoprotein analysed in subgroups by gender.


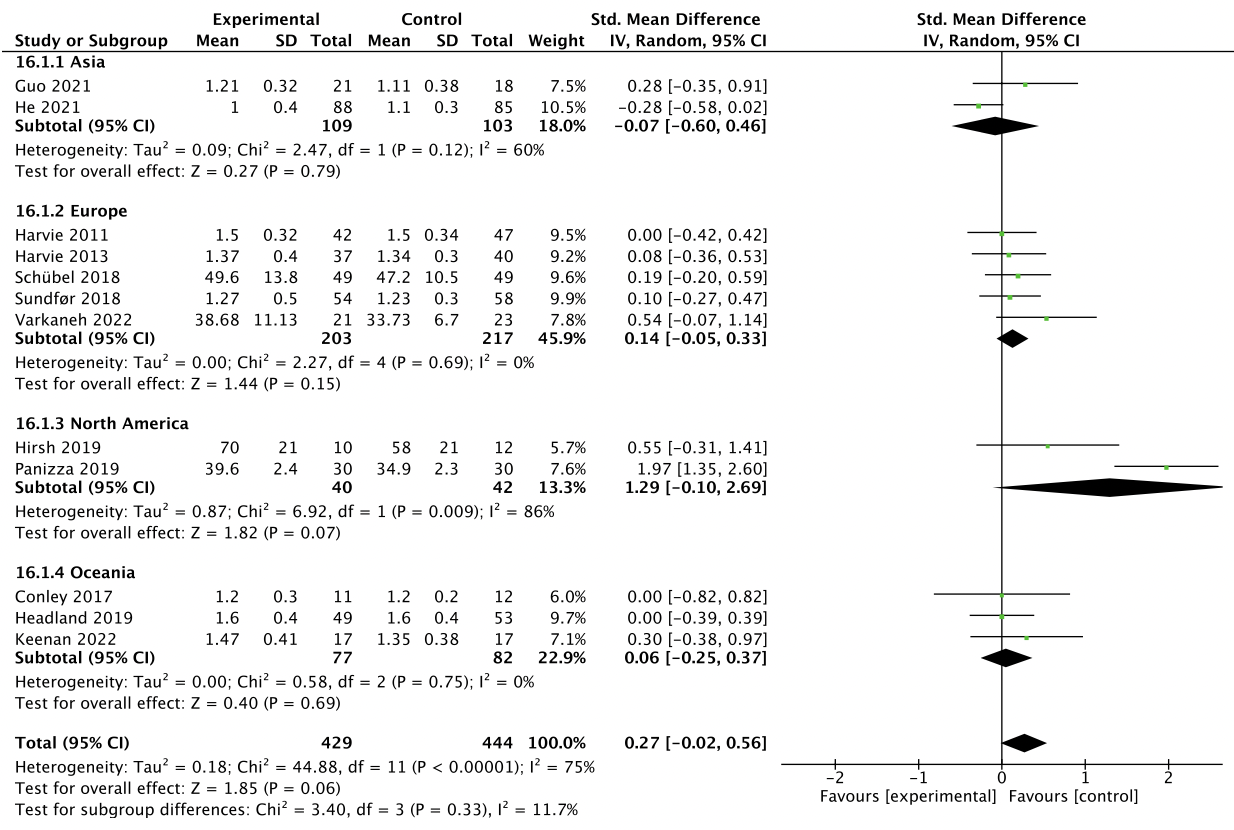


**Supplementary Figure 34.** Forest map of high-density lipoprotein analysed in subgroups by geographic location.


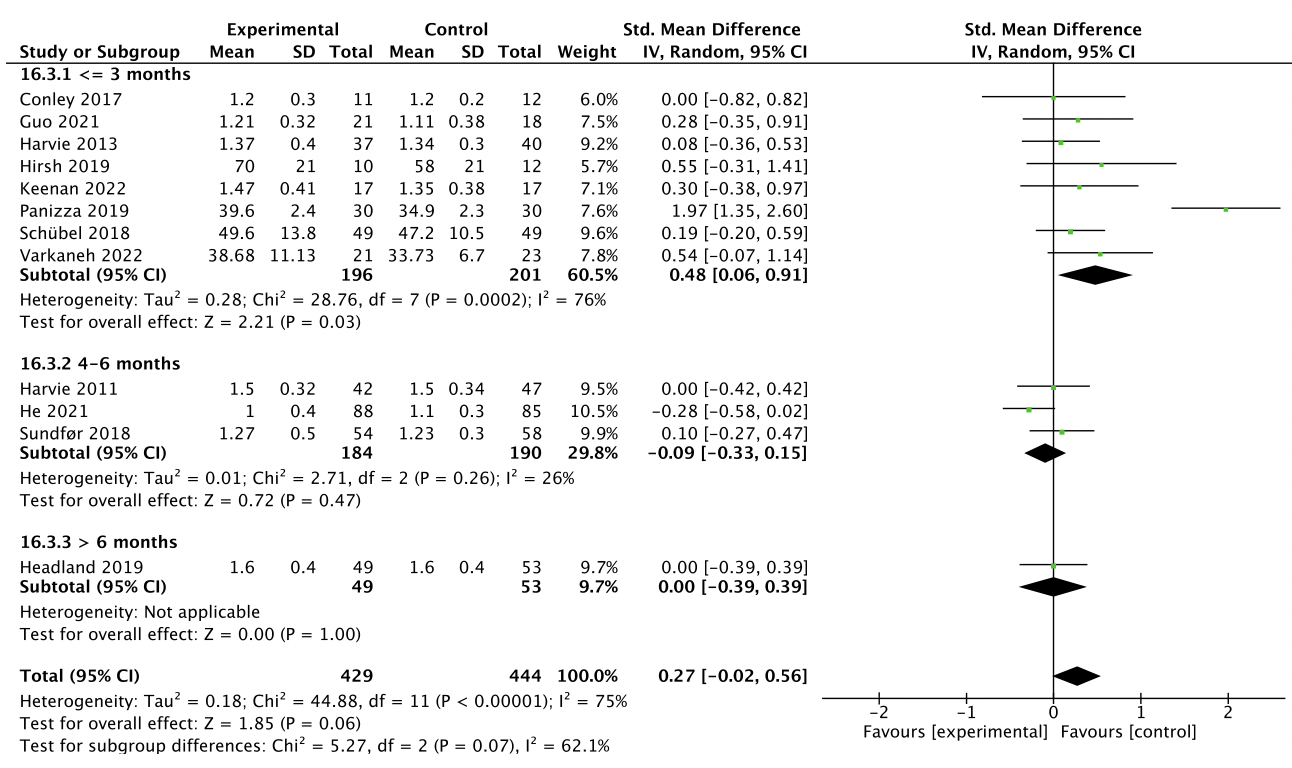


**Supplementary Figure 35.** Forest map of high-density lipoprotein analysed in subgroups by treatment duration.


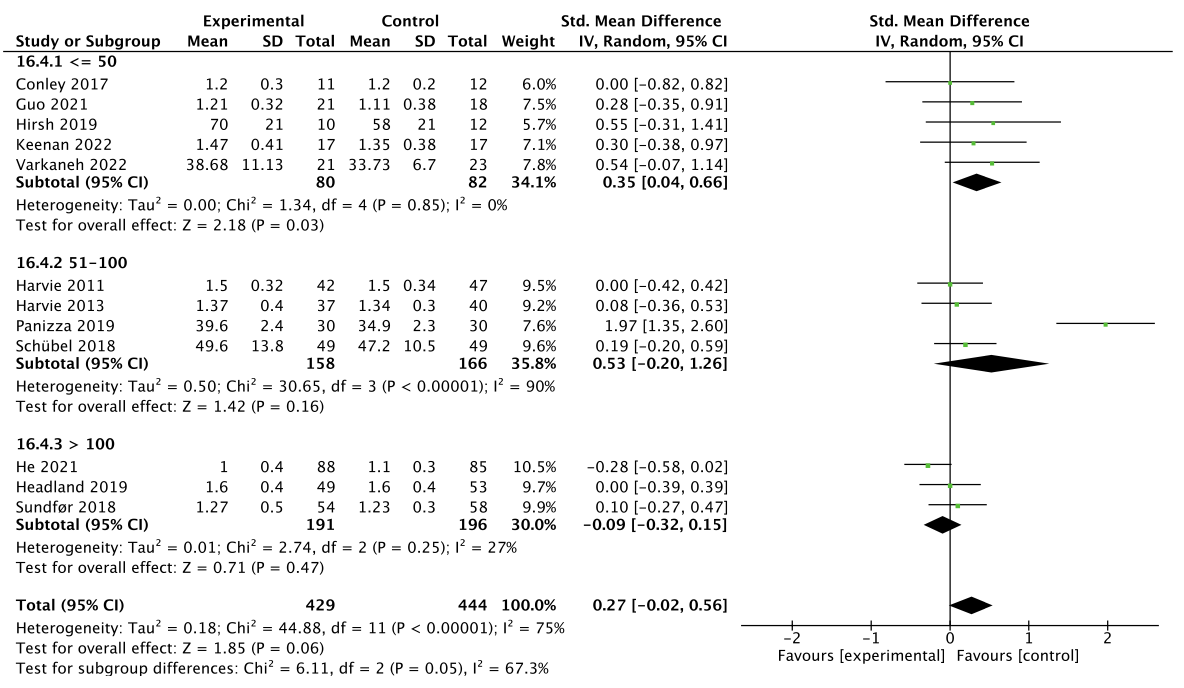


**Supplementary Figure 36.** Forest map of high-density lipoprotein analysed in subgroups by sample size.


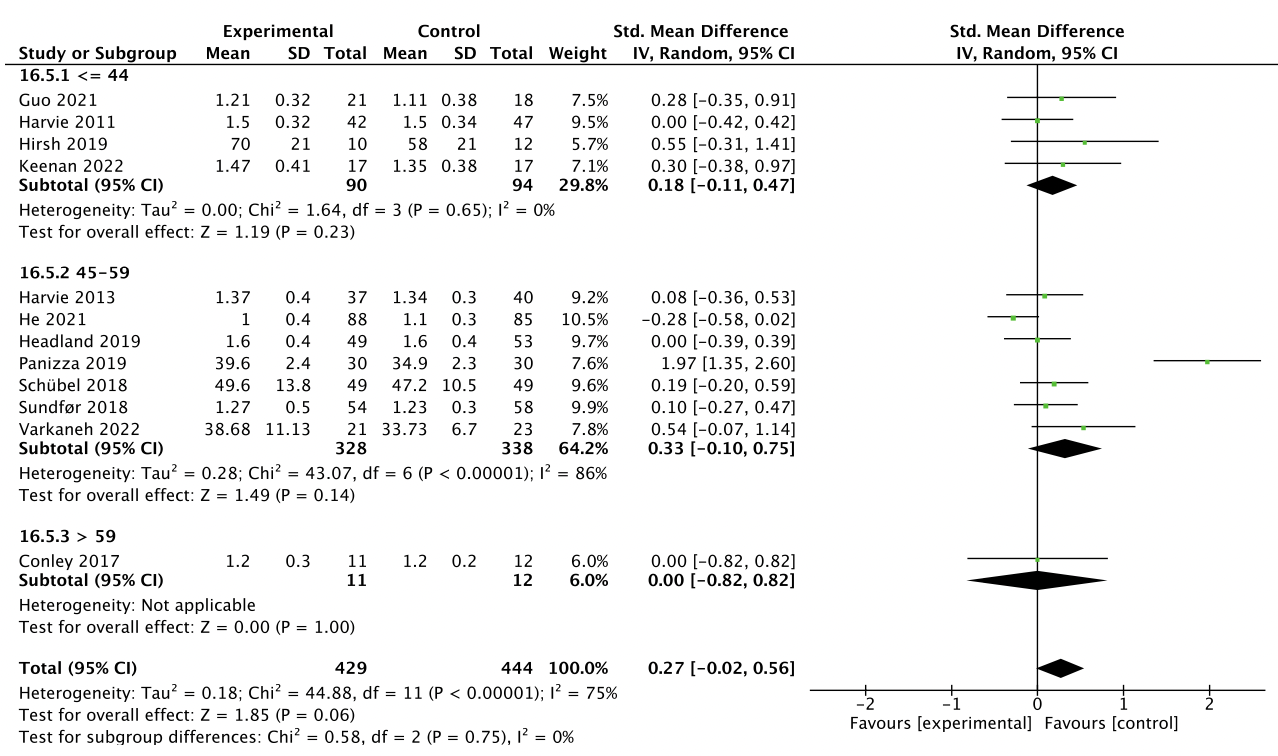


**Supplementary Figure 37.** Forest map of high-density lipoprotein analysed in subgroups by age.


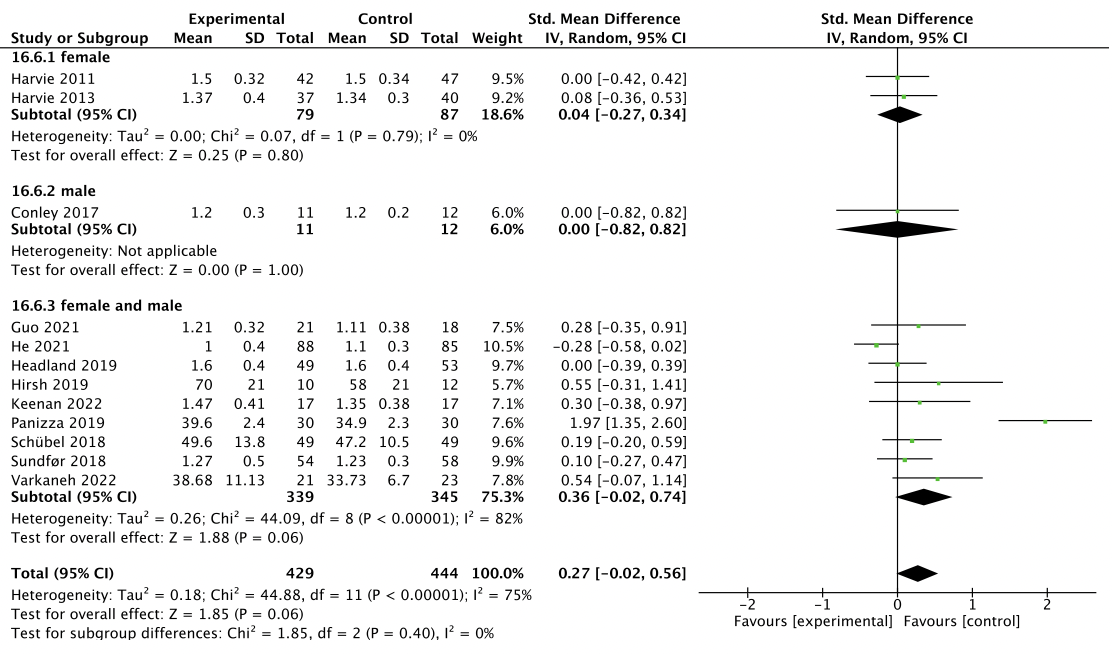


**Supplementary Figure 38.** Forest map of high-density lipoprotein analysed in subgroups by gender.


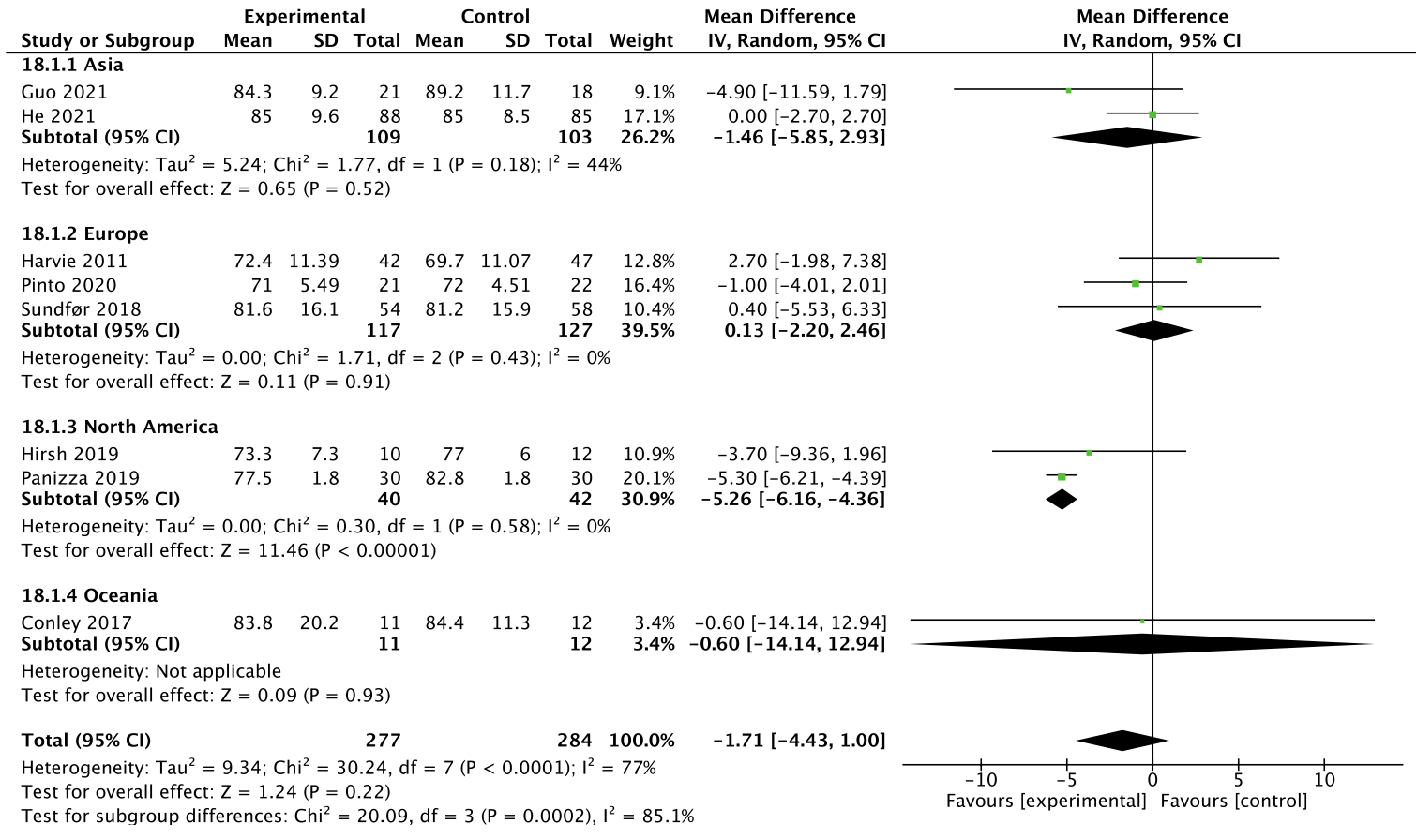


**Supplementary Figure 39.** Forest map of diastolic blood pressure analysed in subgroups by geographic location.


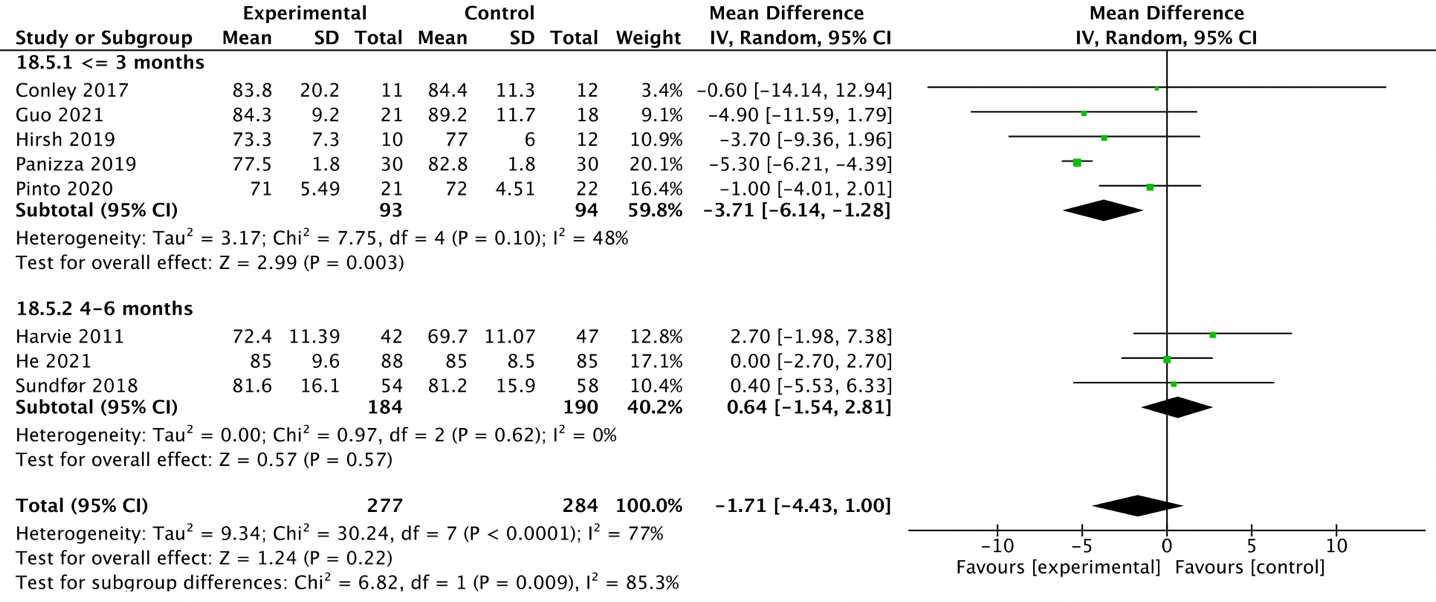


**Supplementary Figure 40.** Forest map of diastolic blood pressure analysed in subgroups by treatment duration.


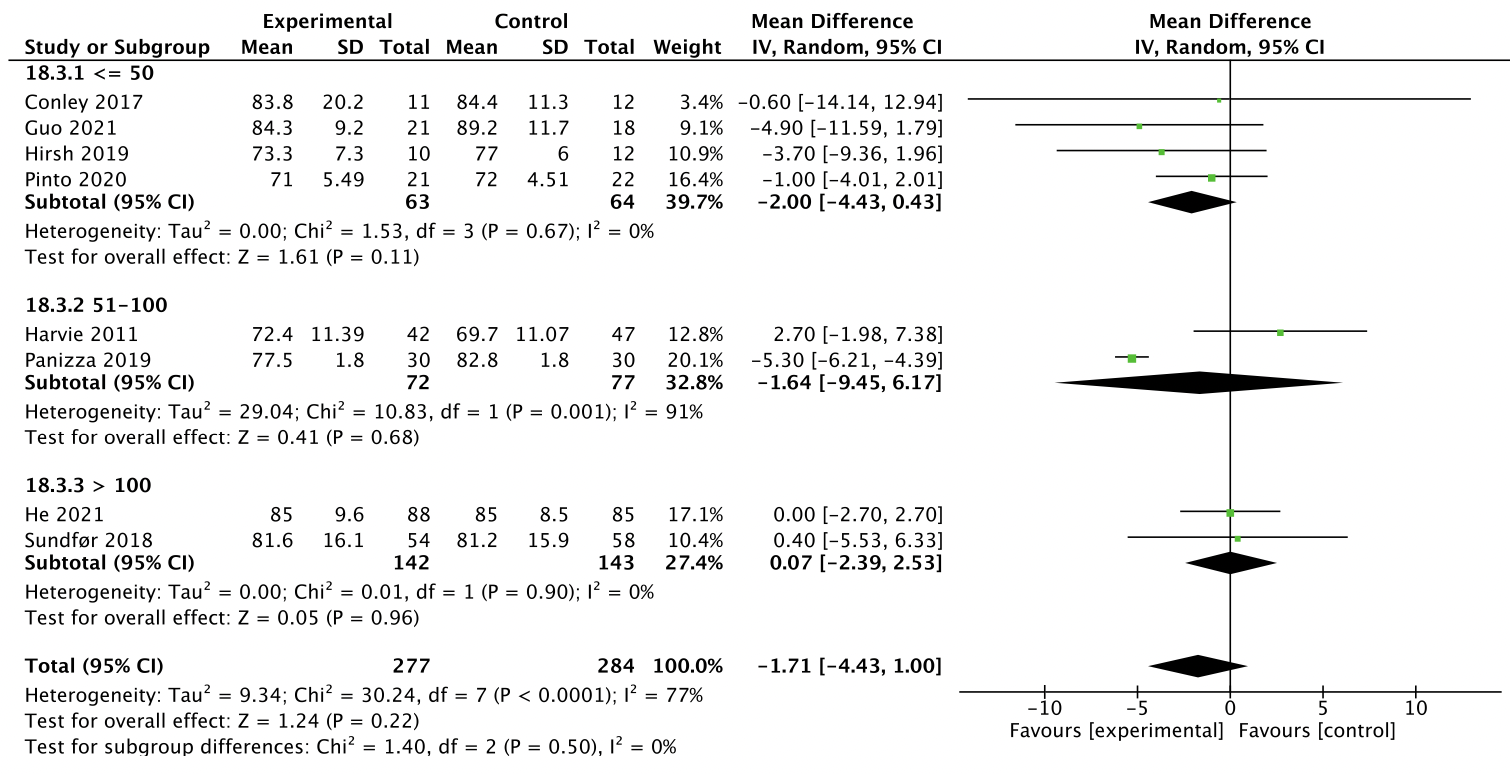


**Supplementary Figure 41.** Forest map of diastolic blood pressure analysed in subgroups by sample size.


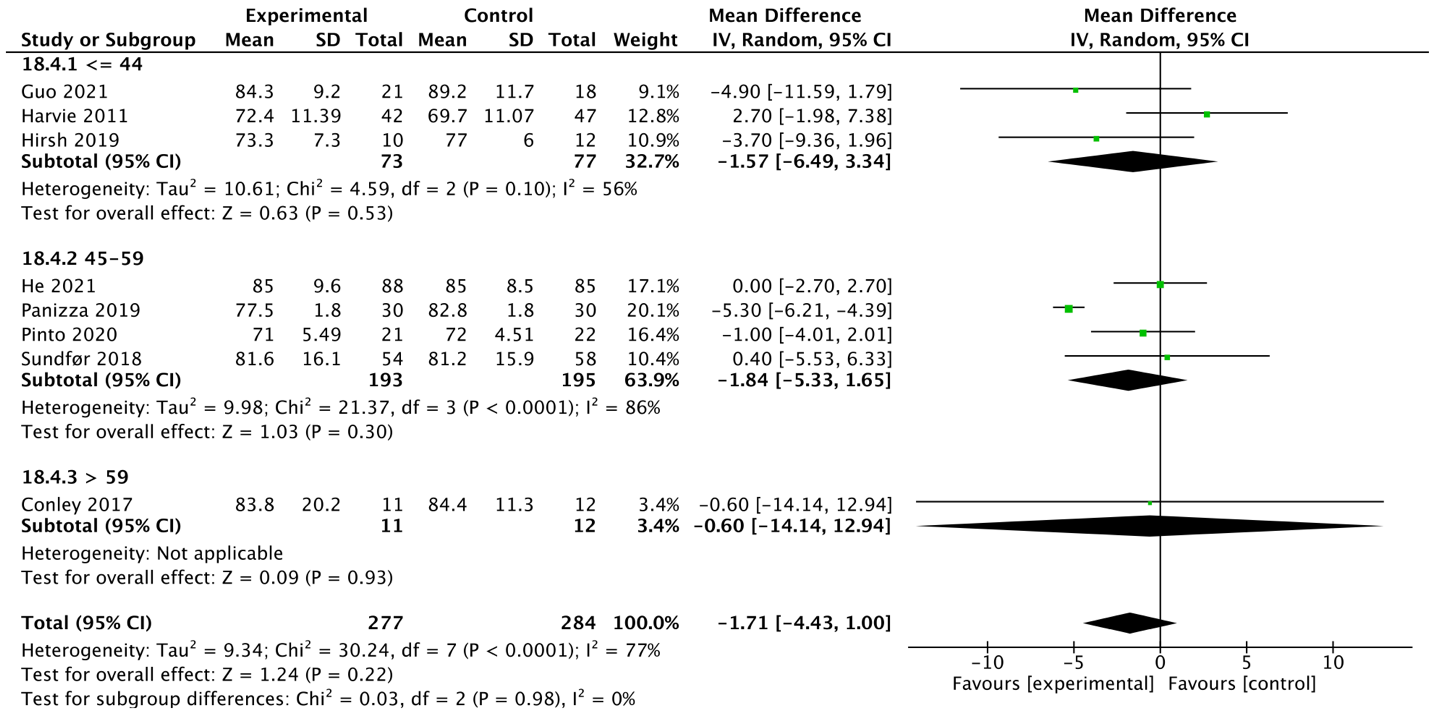


**Supplementary Figure 42.** Forest map of diastolic blood pressure analysed in subgroups by age.


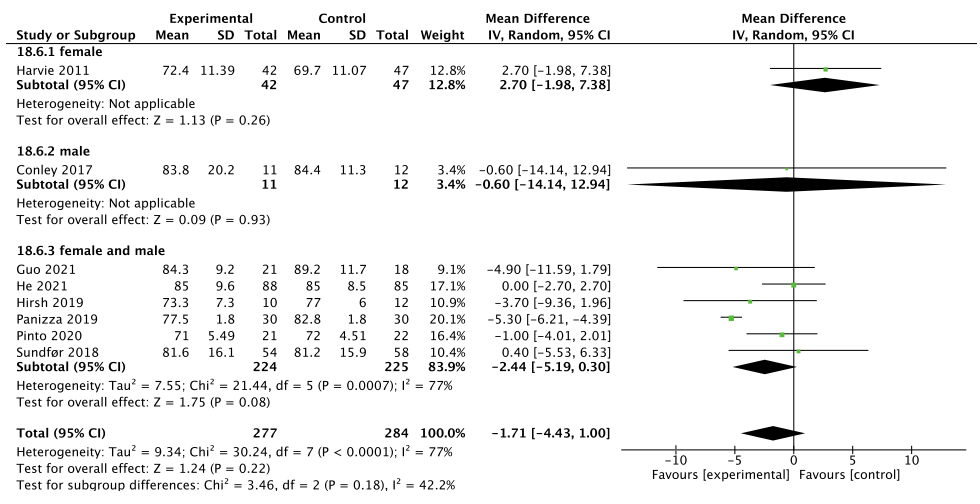


**Supplementary Figure 43.** Forest map of diastolic blood pressure analysed in subgroups by gender.


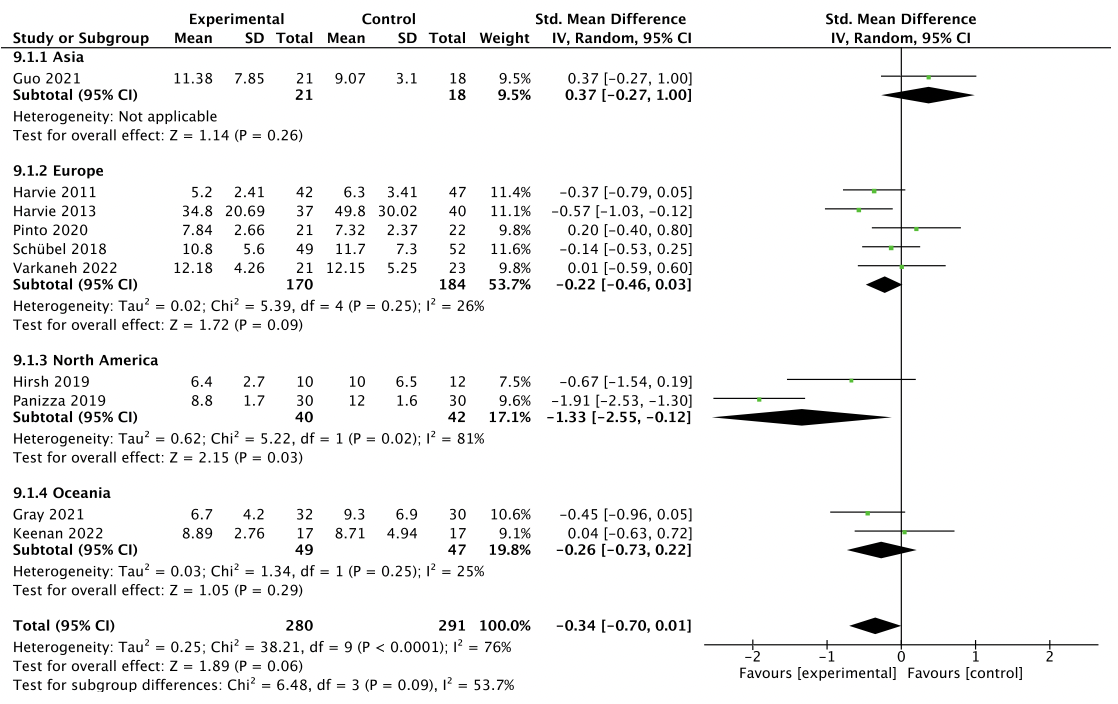


**Supplementary Figure 44.** Forest map of insulin analysed in subgroups by geographic location.


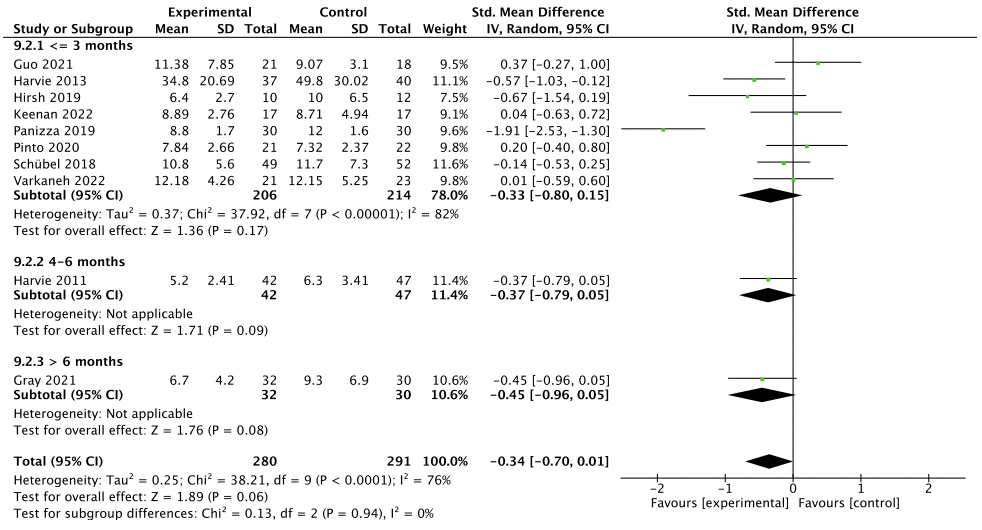


**Supplementary Figure 45.** Forest map of insulin analysed in subgroups by treatment duration.


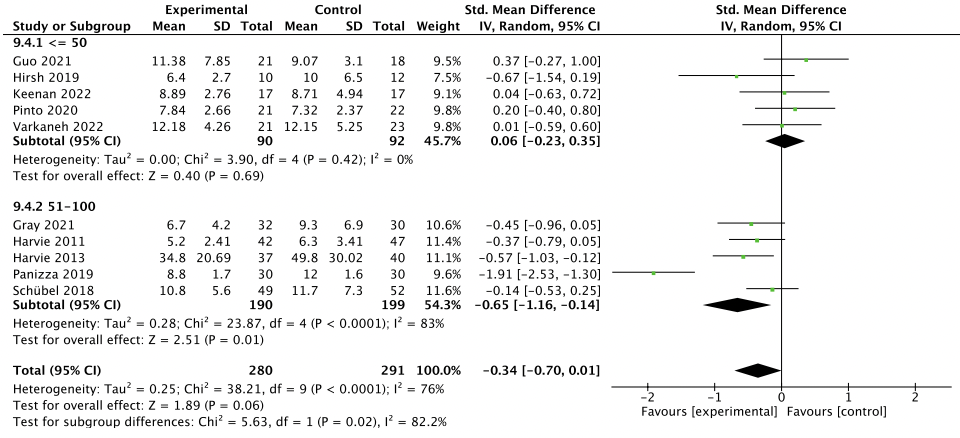


**Supplementary Figure 46.** Forest map of insulin analysed in subgroups by sample size.


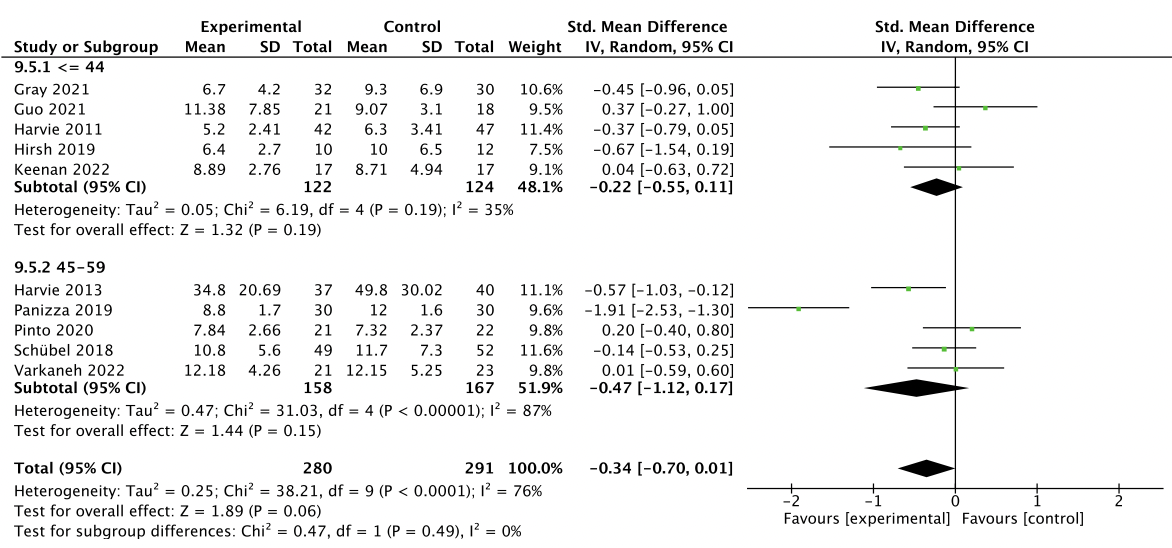


**Supplementary Figure 47.** Forest map of insulin analysed in subgroups by age.


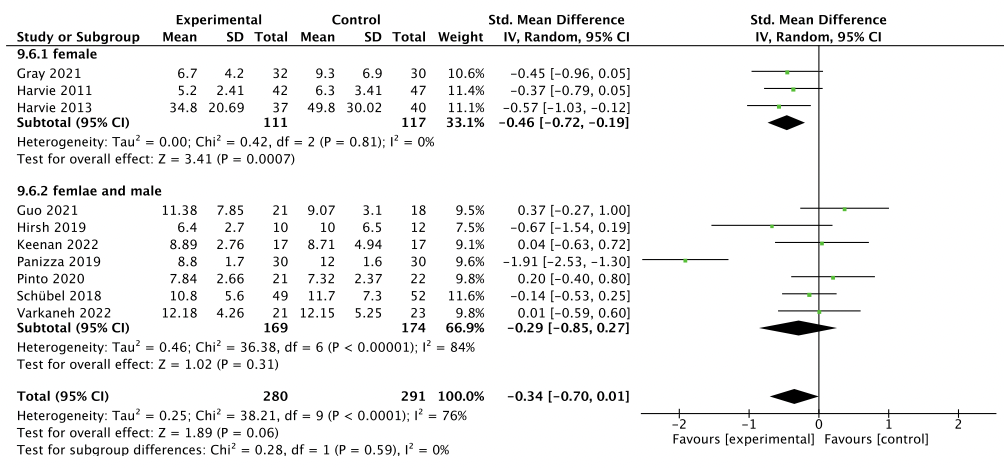


**Supplementary Figure 48.** Forest map of insulin analysed in subgroups by gender.


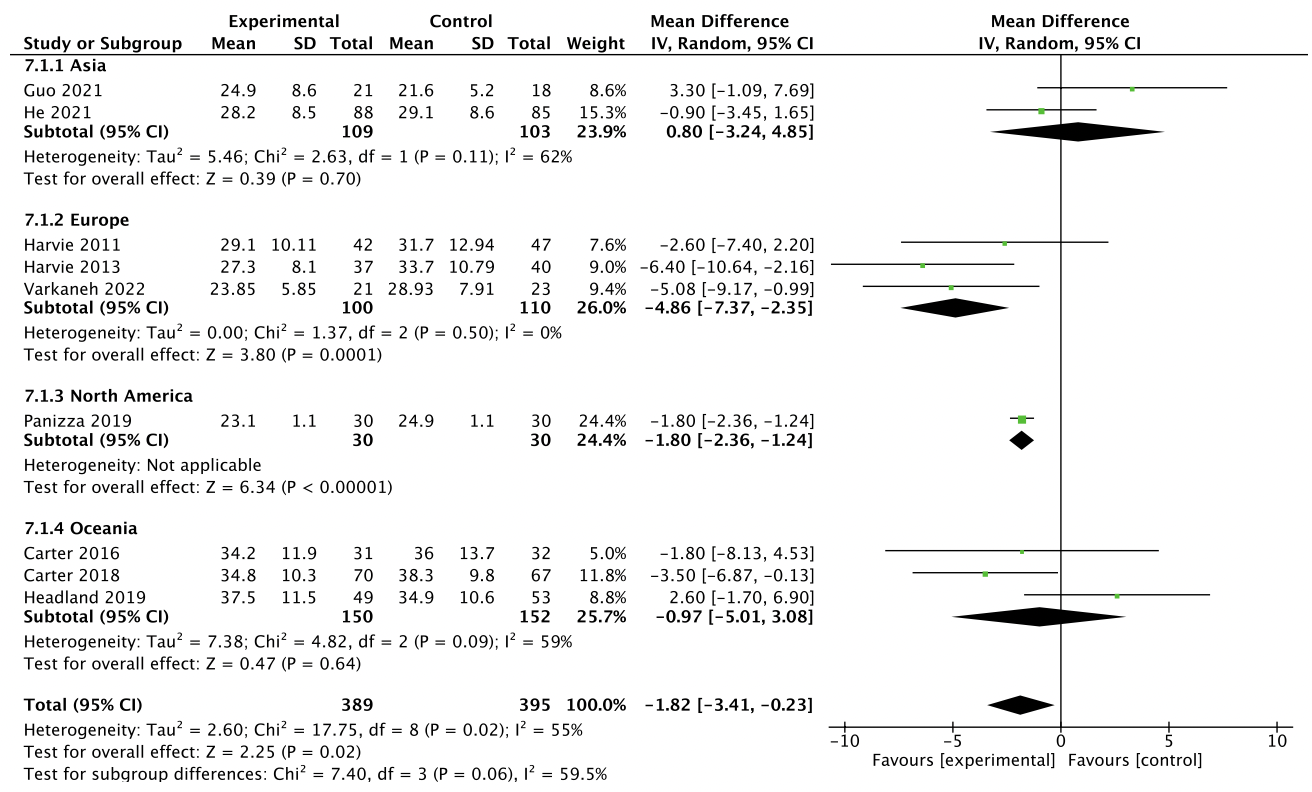


**Supplementary Figure 49.** Forest map of fat mass analysed in subgroups by geographic location.


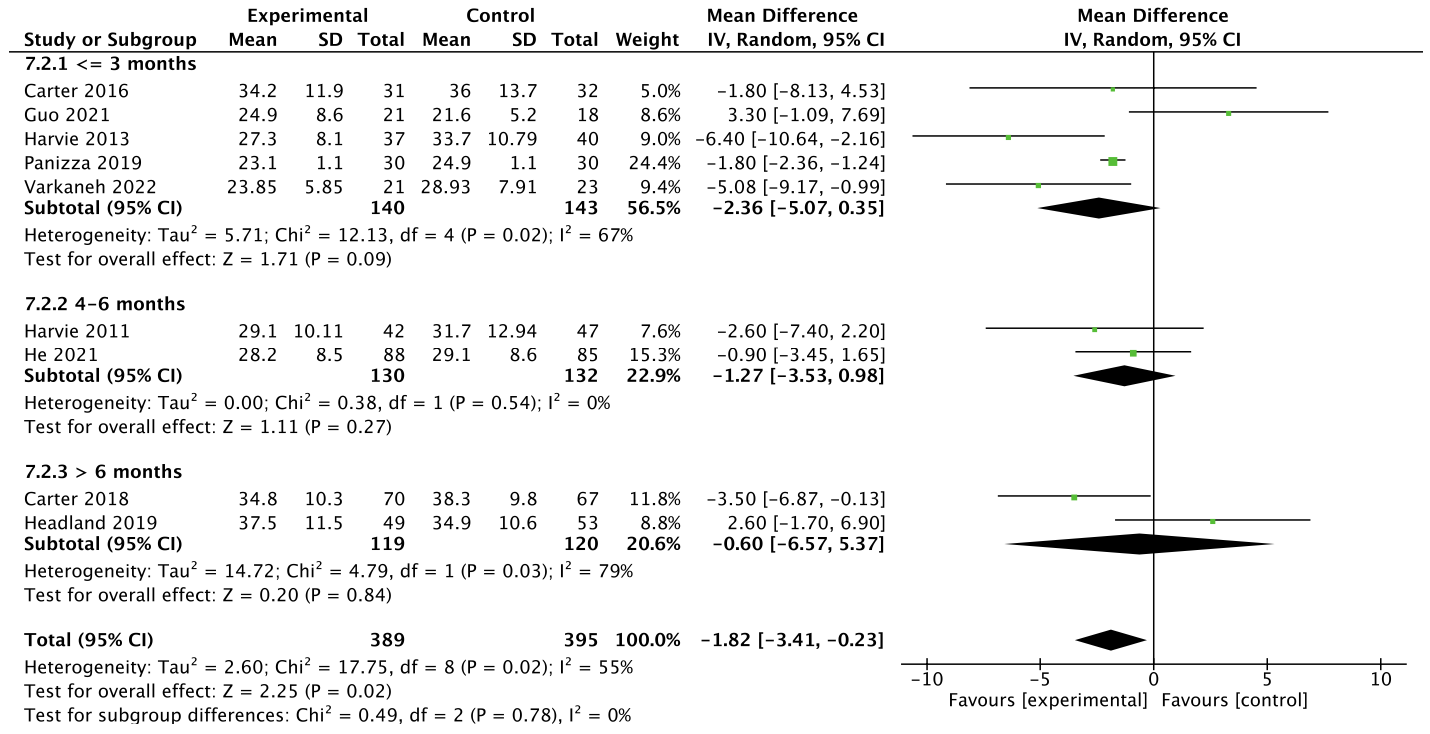


**Supplementary Figure 50.** Forest map of fat mass analysed in subgroups by treatment duration.


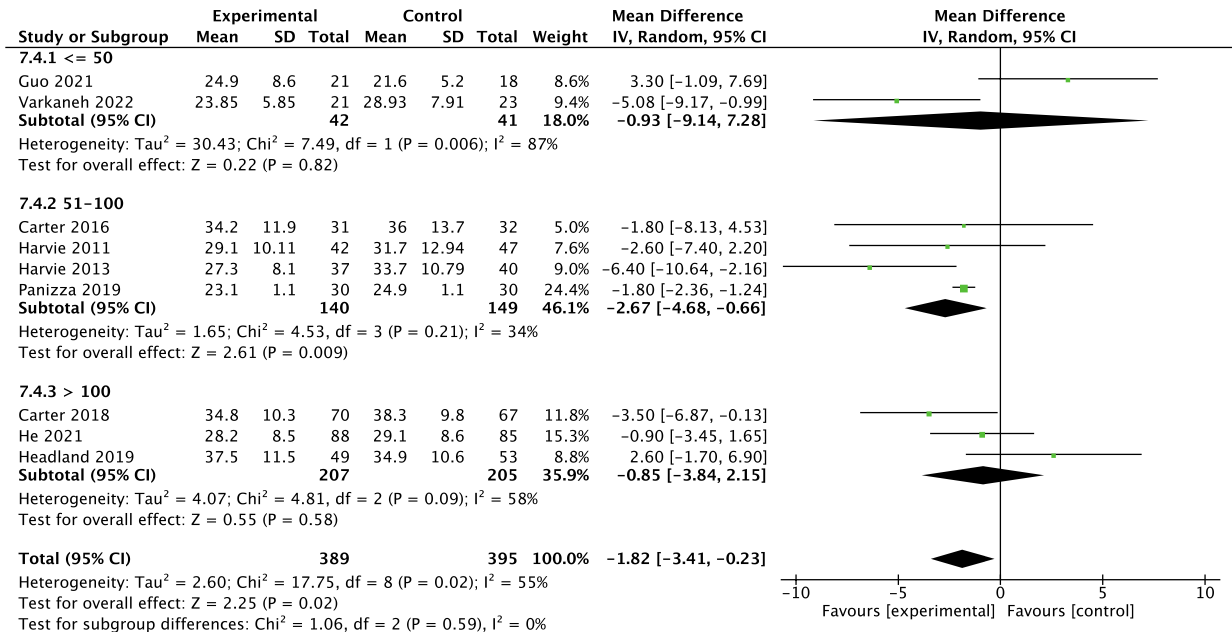


**Supplementary Figure 51.** Forest map of fat mass analysed in subgroups by sample size.


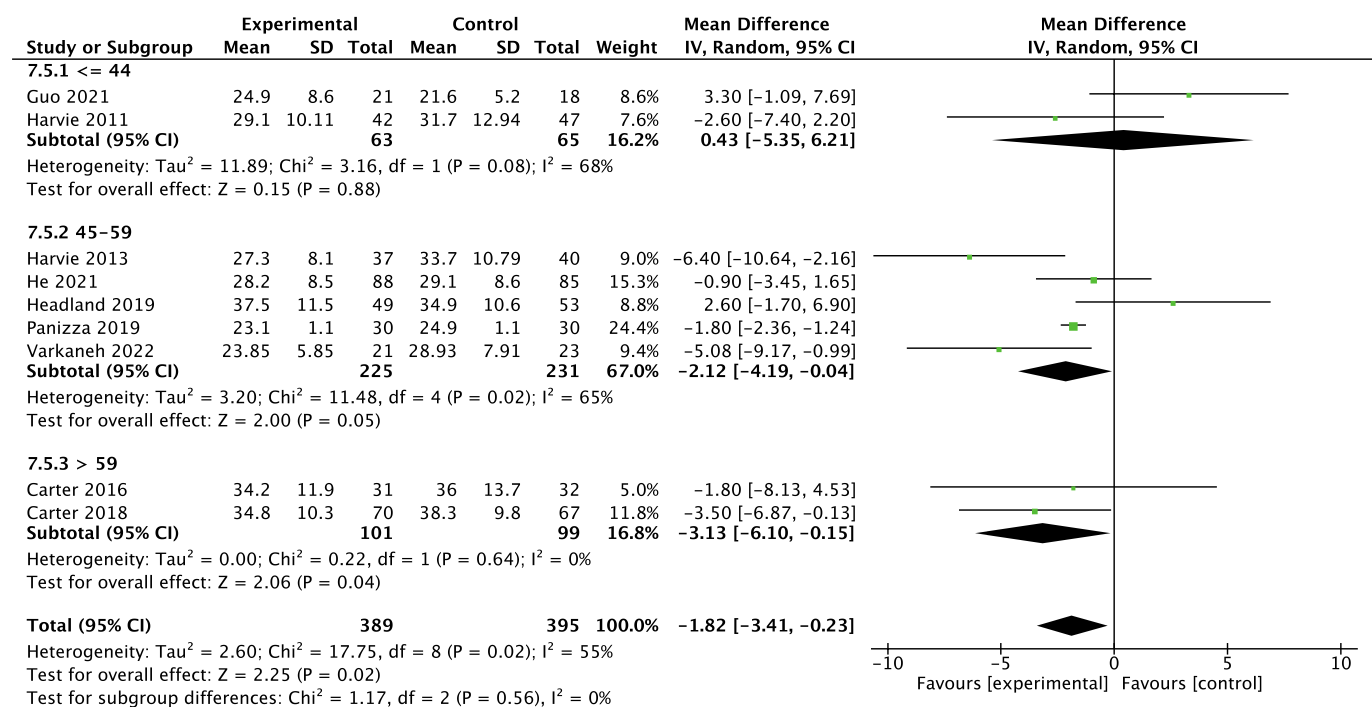


**Supplementary Figure 52.** Forest map of fat mass analysed in subgroups by age.


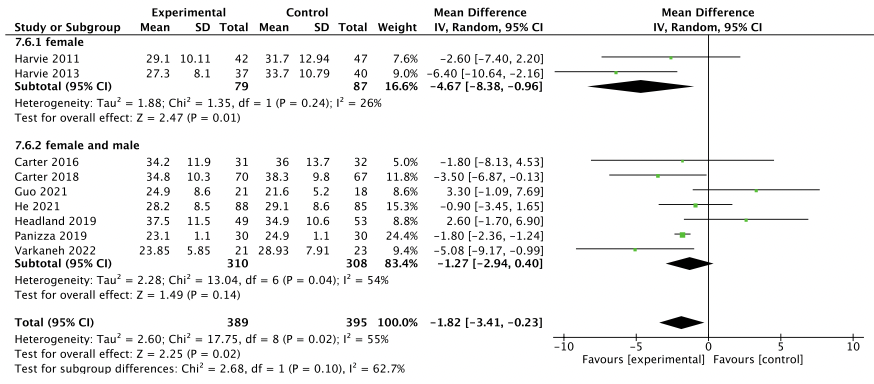


**Supplementary Figure 53.** Forest map of fat mass analysed in subgroups by gender.

## Supplementary TABLEs

TABLE 1 PRISMA checklist

| **Section and Topic** | **Item #** | **Checklist item** | **Location where item is reported** |
| --- | --- | --- | --- |
| **TITLE** | | |  |
| Title | 1 | Identify the report as a systematic review. | 1 |
| **ABSTRACT** | | |  |
| Abstract | 2 | See the PRISMA 2020 for Abstracts checklist. | 1 |
| **INTRODUCTION** | | |  |
| Rationale | 3 | Describe the rationale for the review in the context of existing knowledge. | 1-2 |
| Objectives | 4 | Provide an explicit statement of the objective(s) or question(s) the review addresses. | 1-2 |
| **METHODS** | | |  |
| Eligibility criteria | 5 | Specify the inclusion and exclusion criteria for the review and how studies were grouped for the syntheses. | 2 |
| Information sources | 6 | Specify all databases, registers, websites, organisations, reference lists and other sources searched or consulted to identify studies. Specify the date when each source was last searched or consulted. | 2 |
| Search strategy | 7 | Present the full search strategies for all databases, registers and websites, including any filters and limits used. | 2 |
| Selection process | 8 | Specify the methods used to decide whether a study met the inclusion criteria of the review, including how many reviewers screened each record and each report retrieved, whether they worked independently, and if applicable, details of automation tools used in the process. | 2 |
| Data collection process | 9 | Specify the methods used to collect data from reports, including how many reviewers collected data from each report, whether they worked independently, any processes for obtaining or confirming data from study investigators, and if applicable, details of automation tools used in the process. | 2 |
| Data items | 10a | List and define all outcomes for which data were sought. Specify whether all results that were compatible with each outcome domain in each study were sought (e.g. for all measures, time points, analyses), and if not, the methods used to decide which results to collect. | 2 |
|  | 10b | List and define all other variables for which data were sought (e.g. participant and intervention characteristics, funding sources). Describe any assumptions made about any missing or unclear information. | 2 |
| Study risk of bias assessment | 11 | Specify the methods used to assess risk of bias in the included studies, including details of the tool(s) used, how many reviewers assessed each study and whether they worked independently, and if applicable, details of automation tools used in the process. | 2 |
| Effect measures | 12 | Specify for each outcome the effect measure(s) (e.g. risk ratio, mean difference) used in the synthesis or presentation of results. | 2 |
| Synthesis methods | 13a | Describe the processes used to decide which studies were eligible for each synthesis (e.g. tabulating the study intervention characteristics and comparing against the planned groups for each synthesis (item #5)). | 2 |
|  | 13b | Describe any methods required to prepare the data for presentation or synthesis, such as handling of missing summary statistics, or data conversions. | 2 |
|  | 13c | Describe any methods used to tabulate or visually display results of individual studies and syntheses. | 2 |
|  | 13d | Describe any methods used to synthesize results and provide a rationale for the choice(s). If meta-analysis was performed, describe the model(s), method(s) to identify the presence and extent of statistical heterogeneity, and software package(s) used. | 2 |
|  | 13e | Describe any methods used to explore possible causes of heterogeneity among study results (e.g. subgroup analysis, meta-regression). | 2 |
|  | 13f | Describe any sensitivity analyses conducted to assess robustness of the synthesized results. | 2 |
| Reporting bias assessment | 14 | Describe any methods used to assess risk of bias due to missing results in a synthesis (arising from reporting biases). | 2 |
| Certainty assessment | 15 | Describe any methods used to assess certainty (or confidence) in the body of evidence for an outcome. | 2 |
| **RESULTS** | | |  |
| Study selection | 16a | Describe the results of the search and selection process, from the number of records identified in the search to the number of studies included in the review, ideally using a flow diagram. | 3 |
|  | 16b | Cite studies that might appear to meet the inclusion criteria, but which were excluded, and explain why they were excluded. | 3 |
| Study characteristics | 17 | Cite each included study and present its characteristics. | 3 |
| Risk of bias in studies | 18 | Present assessments of risk of bias for each included study. | 3 |
| Results of individual studies | 19 | For all outcomes, present, for each study: (a) summary statistics for each group (where appropriate) and (b) an effect estimate and its precision (e.g. confidence/credible interval), ideally using structured tables or plots. | 3 |
| Results of syntheses | 20a | For each synthesis, briefly summarise the characteristics and risk of bias among contributing studies. | 3-6 |
|  | 20b | Present results of all statistical syntheses conducted. If meta-analysis was done, present for each the summary estimate and its precision (e.g. confidence/credible interval) and measures of statistical heterogeneity. If comparing groups, describe the direction of the effect. | 3-6 |
|  | 20c | Present results of all investigations of possible causes of heterogeneity among study results. | 3-6 |
|  | 20d | Present results of all sensitivity analyses conducted to assess the robustness of the synthesized results. | 3-6 |
| Reporting biases | 21 | Present assessments of risk of bias due to missing results (arising from reporting biases) for each synthesis assessed. | 3-7 |
| Certainty of evidence | 22 | Present assessments of certainty (or confidence) in the body of evidence for each outcome assessed. | 3-7 |
| **DISCUSSION** | | |  |
| Discussion | 23a | Provide a general interpretation of the results in the context of other evidence. | 7 |
|  | 23b | Discuss any limitations of the evidence included in the review. | 8 |
|  | 23c | Discuss any limitations of the review processes used. | 8 |
|  | 23d | Discuss implications of the results for practice, policy, and future research. | 7-8 |
| **OTHER INFORMATION** | | |  |
| Registration and protocol | 24a | Provide registration information for the review, including register name and registration number, or state that the review was not registered. | 2 |
|  | 24b | Indicate where the review protocol can be accessed, or state that a protocol was not prepared. | 2 |
|  | 24c | Describe and explain any amendments to information provided at registration or in the protocol. | 2 |
| Support | 25 | Describe sources of financial or non-financial support for the review, and the role of the funders or sponsors in the review. | 9 |
| Competing interests | 26 | Declare any competing interests of review authors. | 9 |
| Availability of data, code and other materials | 27 | Report which of the following are publicly available and where they can be found: template data collection forms; data extracted from included studies; data used for all analyses; analytic code; any other materials used in the review. | 9 |

TABLE 2 Search strategy in PubMed

| Search number | Query |
| --- | --- |
| #1 | "Overweight"[Mesh] |
| #2 | "Obesity"[Mesh] |
| #3 | “overweight”[Title/Abstract] OR “obesity”[Title/Abstract] OR “obese”[Title/Abstract] |
| #4 | #1 OR #2 OR #3  "Overweight"[Title/Abstract] OR "Obesity"[Title/Abstract] OR "obese"[Title/Abstract] OR "Obesity"[MeSH Terms] OR "Overweight"[MeSH Terms] |
| #5 | "Intermittent Fasting"[MeSH Terms] |
| #6 | "intermittent fasting"[Title/Abstract] OR "fasting intermittent"[Title/Abstract] OR "meal skipping"[Title/Abstract] OR "skipping meal"[Title/Abstract] OR "time restricted fasting"[Title/Abstract] OR "fasting time restricted"[Title/Abstract] OR "time restricted eating"[Title/Abstract] OR "eating time restricted"[Title/Abstract] OR "time restricted feeding"[Title/Abstract] OR "feeding time restricted"[Title/Abstract] OR "time restricted feedings"[Title/Abstract] OR "2 days week"[Title/Abstract] |
| #7 | #5 OR #6  ("Intermittent Fasting"[MeSH Terms]) OR ("intermittent fasting"[Title/Abstract] OR "fasting intermittent"[Title/Abstract] OR "meal skipping"[Title/Abstract] OR "skipping meal"[Title/Abstract] OR "time restricted fasting"[Title/Abstract] OR "fasting time restricted"[Title/Abstract] OR "time restricted eating"[Title/Abstract] OR "eating time restricted"[Title/Abstract] OR "time restricted feeding"[Title/Abstract] OR "feeding time restricted"[Title/Abstract] OR "time restricted feedings"[Title/Abstract] OR "2 days week"[Title/Abstract]) |
| #8 | #4 AND #7  ("Overweight"[Title/Abstract] OR "Obesity"[Title/Abstract] OR "obese"[Title/Abstract] OR "Obesity"[MeSH Terms] OR "Overweight"[MeSH Terms]) AND ("Intermittent Fasting"[MeSH Terms]) OR ("intermittent fasting"[Title/Abstract] OR "fasting intermittent"[Title/Abstract] OR "meal skipping"[Title/Abstract] OR "skipping meal"[Title/Abstract] OR "time restricted fasting"[Title/Abstract] OR "fasting time restricted"[Title/Abstract] OR "time restricted eating"[Title/Abstract] OR "eating time restricted"[Title/Abstract] OR "time restricted feeding"[Title/Abstract] OR "feeding time restricted"[Title/Abstract] OR "time restricted feedings"[Title/Abstract] OR "2 days week"[Title/Abstract]) |
| #9 | "Randomized Controlled Trial"[Publication Type] |
| #10 | "random"[Title/Abstract] OR "Randomized"[Title/Abstract] OR "control trial"[Title/Abstract] OR "randomization test"[Title/Abstract] OR "randomised trials"[Title/Abstract] OR "randomized trial"[Title/Abstract] OR "randomised"[Title/Abstract] OR "randomized controlled trial"[Title/Abstract] |
| #11 | #9 OR #10  "Randomized Controlled Trial"[Publication Type] OR "random"[Title/Abstract] OR "Randomized"[Title/Abstract] OR "control trial"[Title/Abstract] OR "randomization test"[Title/Abstract] OR "randomised trials"[Title/Abstract] OR "randomized trial"[Title/Abstract] OR "randomised"[Title/Abstract] OR "Randomized Controlled Trial"[Title/Abstract] |
| #12 | #8 AND # 11  (("Overweight"[Title/Abstract] OR "Obesity"[Title/Abstract] OR "obese"[Title/Abstract] OR "Obesity"[MeSH Terms] OR "Overweight"[MeSH Terms]) AND ("Intermittent Fasting"[MeSH Terms]) OR ("intermittent fasting"[Title/Abstract] OR "fasting intermittent"[Title/Abstract] OR "meal skipping"[Title/Abstract] OR "skipping meal"[Title/Abstract] OR "time restricted fasting"[Title/Abstract] OR "fasting time restricted"[Title/Abstract] OR "time restricted eating"[Title/Abstract] OR "eating time restricted"[Title/Abstract] OR "time restricted feeding"[Title/Abstract] OR "feeding time restricted"[Title/Abstract] OR "time restricted feedings"[Title/Abstract] OR "2 days week"[Title/Abstract])) AND ("Randomized Controlled Trial"[Publication Type] OR "random"[Title/Abstract] OR "Randomized"[Title/Abstract] OR "control trial"[Title/Abstract] OR "randomization test"[Title/Abstract] OR "randomised trials"[Title/Abstract] OR "randomized trial"[Title/Abstract] OR "randomised"[Title/Abstract] OR "Randomized Controlled Trial"[Title/Abstract]) |

TABLE 3 GRADE quality of evidence evaluation form

| Indicators/  Number of studies | Quality evaluation | | | | | Sample size (T/C) | Effect size (95% CI) | Level of evidence |
| --- | --- | --- | --- | --- | --- | --- | --- | --- |
|  | Study limitations | Inconsistency | Indirectness | Imprecision | Reporting bias |  |  |  |
| Body weight/17 | a | b | none | none | none | 636/651 | -1.88 (-3.67, -0.10) | Low |
| BMI/12 | a | b | none | none | none | 374/370 | -0.85 (-1.52, -0.17) | Low |
| Waist circumference/9 | a | none | none | none | none | 253/266 | -2.77 (-3.48, -2.07) | Moderate |
| Body fat percentage/8 | a | none | none | none | none | 323/324 | -0.77 (-1.29, -0.26) | Moderate |
| Total cholesterol/12 | a | b | none | none | none | 429/444 | -0.23 (-0.51, 0.05) | Low |
| Triglycerides/13 | a | b | none | none | none | 443/455 | -0.25 (-0.56, 0.07) | Low |
| Low-density lipoprotein/12 | a | b | none | none | none | 429/444 | -0.24 (-0.45, -0.03) | Low |
| High-density lipoprotein/12 | a | b | none | none | none | 429/444 | 0.27 (-0.02, 0.56) | Low |
| Systolic blood pressure/9 | a | none | none | none | none | 314/324 | -2.93 (-4.06, -1.81) | Moderate |
| Diastolic blood pressure/8 | a | b | none | none | none | 277/284 | -1.71 (-4.43, 1.00) | Low |
| HOMA-IR/8 | a | none | none | none | none | 229/239 | -0.33 (-0.52, -0.13) | Moderate |
| Insulin/10 | a | b | none | none | none | 280/288 | -0.34 (-0.70, 0.01) | Low |
| FBG/12 | a | none | none | none | none | 384/399 | -0.03 (-0.17, 0.11) | Moderate |
| HbA1c/7 | a | none | none | none | none | 333/334 | -0.01 (-0.16, 0.14) | Moderate |
| Hip circumference/6 | a | none | none | none | none | 200/209 | -1.39 (-2.01, -0.76) | Moderate |
| Visceral fat/4 | a | b | none | none | none | 146/140 | -0.60 (-1.36, 0.16) | Low |
| Fat mass/9 | a | b | none | none | none | 389/395 | -1.82 (-3.41, -0.23) | Low |
| Fat-free mass/9 | a | none | none | none | c | 326/335 | -2.17 (-2.90, -1.44) | Low |
| Heart rate/4 | a | b | none | none | none | 106/110 | -3.77 (-8.94, 1.40) | Low |

Note: a: missing blinding, inadequate allocation concealment; b: I^2^ > 50%; c: publication bias; T: test group; C: control group.
